# Drosophila ClC‐a is required in glia of the stem cell niche for proper neurogenesis and wiring of neural circuits

**DOI:** 10.1002/glia.23691

**Published:** 2019-09-03

**Authors:** Haritz Plazaola‐Sasieta, Qi Zhu, Héctor Gaitán‐Peñas, Martín Rios, Raúl Estévez, Marta Morey

**Affiliations:** ^1^ Departament de Genètica, Microbiologia i Estadística, Facultat de Biologia Universitat de Barcelona Barcelona Spain; ^2^ Departament de Ciencies Fisiològiques, Genes, Disease and Therapy Program IDIBELL‐Institute of Neurosciences Universitat de Barcelona, L'Hospitalet de Llobregat Barcelona Spain; ^3^ Centro de Investigación Biomédica en Red de Enfermedades Raras (CIBERER) Instituto de Salud Carlos III Madrid Spain; ^4^ Institut de Biomedicina de la Universitat de Barcelona (IBUB) Programa de Biologia Integrativa Barcelona Spain

**Keywords:** axon guidance, ClC‐a chloride channel, glia, neural circuit assembly, neurogenesis, stem cell niche

## Abstract

Glial cells form part of the neural stem cell niche and express a wide variety of ion channels; however, the contribution of these channels to nervous system development is poorly understood. We explored the function of the *Drosophila ClC‐a* chloride channel, since its mammalian ortholog *CLCN2* is expressed in glial cells, and defective channel function results in leukodystrophies, which in humans are accompanied by cognitive impairment. We found that *ClC‐a* was expressed in the niche in cortex glia, which are closely associated with neurogenic tissues. Characterization of loss‐of‐function *ClC‐a* mutants revealed that these animals had smaller brains and widespread wiring defects. We showed that *ClC‐a* is required in cortex glia for neurogenesis in neuroepithelia and neuroblasts, and identified defects in a neuroblast lineage that generates guidepost glial cells essential for photoreceptor axon guidance. We propose that glia‐mediated ionic homeostasis could nonautonomously affect neurogenesis, and consequently, the correct assembly of neural circuits.

## Main Points

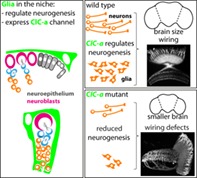

- ClC‐a chloride channel is expressed in glia of the stem cell niche and regulates neurogenesis in a non‐autonomous fashion.
- ClC‐a mutants have reduced neurogenesis, and consequently, smaller brains.
- ClC‐a mutants also display wiring defects.

## 1 INTRODUCTION

The remarkable proliferative capacity of stem cells requires tight regulation to ensure generation of the appropriate amount of cells and tissue homeostasis during development. This regulation is controlled not only by stem cell‐intrinsic programs, but also by extrinsic cues from the surrounding cellular niche. In vertebrate and invertebrate nervous systems, glia form part of the niche for neural stem/progenitor cells (Bjornsson, Apostolopoulou, Tian, & Temple, 2015; Ruddy & Morshead, 2018). In both systems, the effect of glia on neurogenic tissues has mainly been related to the secretion of factors that regulate the maintenance, proliferation, and differentiation of stem and progenitor cells.

One of the findings that changed the earlier view of glia as simply a passive structural element was the observation that glial cells expressed a wide variety of ion channels and neurotransmitter receptors (Barres, 1991; Barres, Chun, & Corey, 1990). Although the physiological roles of several of these ion channels in glia have been described both in normal and pathological states of the mature nervous system (Black & Waxman, 2013; Nwaobi, Cuddapah, Patterson, Randolph, & Olsen, 2016; Olsen et al., 2015; Pappalardo, Black, & Waxman, 2016; Verkhrastsky & Steinhauser, 2000), the contribution of glial ion channel functions specifically in the niche during nervous system development remains poorly understood.

Among the ion channels expressed in glia, the vertebrate plasma‐membrane ClC‐2 chloride channel has been proposed as one of the channels involved in K^+^ buffering, a key ionic homeostasis process in which glia are involved (Jentsch & Pusch, 2018; H. Wang et al., 2017). In the mature nervous system, increased neural activity leads to an increase in extracellular K^+^, which can alter neuronal excitability. To lower the concentration of K^+^, astrocytes take up the ion and distribute it to distant sites via the astrocytic syncytia. Uptake of K^+^ occurs concomitantly with uptake of Cl^−^ and water, producing transient astrocyte swelling (Bellot‐Saez, Kékesi, Morley, & Buskila, 2017). Based on its expression in astrocytic glia, the ClC‐2 channel has been proposed as one of the channels that might participate in this Cl^−^ uptake (Blanz et al., 2007; Hoegg‐Beiler et al., 2014; Sirisi et al., 2017). Mutations in *CLCN2*, which codes for ClC‐2, are responsible for leukoencephalopathy with ataxia (LKPAT) (Depienne et al., 2013) and ClC‐2 has been related to megalencephalic leukoencephalopathy with subcortical cysts (MLC) (Hoegg‐Beiler et al., 2014; Jeworutzki et al., 2012; Sirisi et al., 2017). Both conditions are characterized by vacuolization of white matter and edema, most probably as a consequence of impaired K^+^ buffering, but patients can also present learning disabilities and mild to moderate intellectual impairment. The finding that ClC‐2 is expressed during development in glial precursors and is required for their differentiation (Hou et al., 2018), together with the fact that intellectual impairment can arise from connectivity defects, suggests that this channel may have additional functions during neural development. To explore this possibility, we leveraged the functional parallelisms between vertebrate and *Drosophila* glia (Chotard & Salecker, 2004; Corty & Freeman, 2013; Freeman & Doherty, 2006) and used the fly, where neurogenesis has been extensively studied, the niche is simpler than in vertebrates, and the *ClC‐a* gene codes for the fly homolog of the vertebrate ClC‐2 chloride channel.

The fly central nervous system contains three structures: the central brain (CB), the ventral nerve cord (VNC), and the optic lobe (OL). The CB and VNC are generated by neural stem cells called neuroblasts that delaminate from the neuroectoderm during embryonic development and give rise to larval and adult brain through two rounds of neurogenesis (Doe, 2008). The OL originates from a group of neuroepithelial cells that proliferates and separates into two crescent shaped primordia, the outer and inner proliferation centers (OPC and IPC), which produce neuroblasts and precursor cells of the different visual processing centers (Apitz & Salecker, 2014). In addition, the OL has been extensively used as a model to explore neural circuit assembly (Plazaola‐Sasieta, Fernández‐Pineda, Zhu, & Morey, 2017), primarily because the modular nature and stereotyped development of the fly eye enable easy detection of wiring defects in photoreceptors and other visual system neurons.

The cellular components in the fly niche comprise the neurogenic cells themselves (neuroepithelia and/or neuroblasts and precursor cells), the newly generated neurons, and three types of glia. Of these latter, the perineural and subperineural glia are components of the blood brain barrier (BBB) that respond to systemic nutritional cues and signal to neuroblasts to proliferate (Chell & Brand, 2010; Kanai et al., 2018; Perez‐Gomez, Slovakova, Rives‐Quinto, Krejci, & Carmena, 2013; Sousa‐Nunes, Yee, & Gould, 2011; Spéder & Brand, 2014). Cortex glia are large cells that lie beneath the subperineural glia. Nutritional cues and neuroblast signals alike induce cortex glia remodeling to encase neuroblasts and their immediate progeny in a chamber and older neurons individually (Read, 2018; Spéder & Brand, 2018). This close association protects neuroblasts from oxidative stress and nutritional restriction (Bailey et al., 2015; Cheng et al., 2011), and is essential for neuronal survival (Coutinho‐Budd, Sheehan, & Freeman, 2017; Dumstrei, Wang, & Hartenstein, 2003; Pereanu, Shy, & Hartenstein, 2005; Read, 2018; Spéder & Brand, 2018). In the OL, a distinct subtype of cortex glia that expresses miRNA mir‐8 (surface‐associated cortex glia) is in direct contact with the OPC (Morante, Vallejo, Desplan, & Dominguez, 2013). These glial cells send signals that regulate expansion of the neuroepithelium and timely transition from neuroepithelium to neuroblast (Morante et al., 2013). Connectivity is also influenced by glial cells in the visual system, where different types control photoreceptor axon pathfinding and targeting (Chotard & Salecker, 2008; Poeck, Fischer, Gunning, Zipursky, & Salecker, 2001).

The electrophysiological properties of *Drosophila* ClC‐a are very similar to those of its mammalian counterpart (Flores, Niemeyer, Sepúlveda, & Cid, 2009; Jeworutzki et al., 2012). In addition, both ClC‐2 and ClC‐a are most abundant in epithelia and the brain. ClC‐2 has been shown to play a role in transepithelial transport in enterocytes (Catalán, Niemeyer, Cid, & Sepúlveda, 2004). Similarly, *ClC‐a* is also expressed in the epithelia of the fly digestive system, and is involved in transepithelial transport in stellate cells of the Malpighian tubules, the fly secretory system (Cabrero et al., 2014; Denholm et al., 2013). In the vertebrate brain, besides glia, ClC‐2 is also expressed in inhibitory neurons, where it regulates neuronal excitability (Földy, Lee, Morgan, & Soltesz, 2010; Ratte & Prescott, 2011; Rinke, Artmann, & Stein, 2010). We were interested in the observation that *ClC‐a* mRNA is expressed in glia in the embryonic fly nervous system (Kearney, Wheeler, Estes, Parente, & Crews, 2004; Tomancak et al., 2002, 2007) and is highly expressed throughout development of the nervous system (Celniker et al., 2009; Rose, Derst, Wanischeck, Marinc, & Walther, 2007), which indicates a possible role of the channel in nervous system development.

In this study, we analyzed the expression pattern of *Drosophila ClC‐a* in the brain, characterized the first loss‐of‐function mutant alleles of this chloride channel and investigated their effects on development of the nervous system. We found that *ClC‐a* is expressed in several types of glia and uncovered a role for this channel in the niche. Its expression in cortex glia, which are in close contact with OPC and IPC neuroepithelial cells and neuroblasts, was necessary for the proper neurogenesis in these cell types, as well as for neuron survival. One of the secondary consequences of reduced neurogenesis was the significantly limited production of guidepost glial cells, which gave rise to nonautonomous neural circuit assembly phenotypes in photoreceptors. Both neurogenic and connectivity defects could be rescued by glial‐specific expression of the rat ClC‐2 vertebrate channel. We propose that the expression of ion channels in the glial niche can shape the development of the nervous system, controlling the number of glia and neurons generated, as well as the connectivity of the latter.

## 2 METHODS

### Genetics

Flies were grown in standard medium at 25°C except for RNAi experiments, which were performed at 29°C. All genotypes analyzed are specified in the Supplementary Information.

Stocks used to characterize *ClC‐a* expression and phenotype were: *MiMIC 05423* (Bloomington Drosophila Stock Center, BDSC 43680), *05423* ^*ClC‐a‐GAL4*^ (BDSC 66801), *MiMIC 14*,*007* (BDSC 59247), *Df(3R)PS2* (BDSC 37742), *mir8‐GAL4* (DGRC 104917), *R54H02‐GAL4* (BDSC 45784), *wrapper932i‐LexA*, *wrapper932i‐GAL80* (Coutinho‐Budd et al., 2017), *repo‐GAL4* on II (Lee & Jones, 2005), *repo‐GAL4* on III (BDSC 7415), UAS‐Dcr2 (Vienna Drosophila Resource Center, VDRC 60009), *UAS‐ClC‐a‐RNAi* (VDRC 110394), *UAS‐ClC‐a* and *UAS‐ClCN2* (this study), *UAS‐slit‐RNAi* (VDRC 108853), *slit* ^*dui*^ (BDSC 9284), *Slit‐GFP* (BDSC 64472), and R9D11‐tdtom (BDSC 35847). Additional stocks used in Supplementary Figures were: *ClC‐a‐GFP* (BDSC 59296), *slit‐lacZ* (*Slit* ^*05428*^ *)* (BDSC 12189), *Rh1GAL4* (BDSC 68385), *Rh4EGFP* (BDSC 7462), *Rh6‐lacZ* (BDSC 8117), *GMR‐GAL4* (BDSC 1104), *R43H01‐LexA* (BDSC 47931) and *R25A01‐GAL4* (BDSC 49102), *gcm‐lacZ* (BDSC 5445), *EGUF/hid FRT82B* (BDSC 5253).

To label membranes and nucleus, we used *UAS‐mCD8‐GFP* (BDSC 5137), *UAS‐mCD8‐RFP.LG* (BDSC 27398), *UAS‐mCD8GFP*, *lexAop‐CD2‐RFP* (BDSC 67093), *UAS‐H2B‐RFP* (Mayer, Emery, Berdnik, Wirtz‐Peitz, & Knoblich, 2005), and *UAS‐H2B‐YFP* (Bellaïche, Gho, Kaltschmidt, Brand, & Schweisguth, 2001), as specified in the genotype list. In experiments where nuclear labeling was used for quantification, the same transgene was employed for control and mutant samples (Figure 5m, Supplementary Figure 6A).

To generate and label neurogenic tissue clones in control and *ClC‐a* mutant backgrounds (Figures 4d,e and 9j‐o), the following stocks were crossed: *hs‐FLP*, *tub‐Gal80*, *FRT19A; tub‐GAL4*, *UAS‐mCD8‐GFP/CyODfYFP; 14*,*007/+* to *FRT19A; +; +* and *hs‐FLP*, *tub‐Gal80*, *FRT19A; tub‐GAL4*, *UAS‐mCD8‐GFP/CyODfYFP; 14*,*007/+* to *FRT19A; +; Df(3R)PS2/TM6B*. Three‐hour egg lays were maintained at 25°C and clone induction was performed at the late L1/early L2 transition in a 37°C water bath. The heat shock protocol for neuroepithelium and neuroblast clones was 30 min and 3 hrs respectively. Brains were dissected 48 hrs after clone induction.

For lineage‐tracing experiments (Figure 6, Supplementary Figure 10D‐G), we used G‐TRACE (*UAS‐RedStinger*, *UAS‐FLP*, *Ubi‐FRT‐stop‐FRT‐Stinger*, BDSC 28280 (Evans et al., 2009) combined with specific *GAL4* drivers.

To perform the surface associated cortex glia and cortex glia‐specific rescue experiments (Figures 4j,k and 8b,c), we devised an intersectional genetic strategy to generate a specific driver. In addition to surface‐associated cortex glia on the OPC, *mir8‐GAL4*, labels cortex glia and neurons in the brain, as well as other cells in the animal. To restrict *mir‐8* expression exclusively to surface‐associated cortex glia and cortex glia, we combined the following transgenes: *repo‐FLP6.2* (Stork, Sheehan, Tasdemir‐Yilmaz, & Freeman, 2014), *tub > GAL80 >* (BDSC 38879), and *mir8‐GAL4*. In this combination, GAL4 is only expressed in cortex glia since the GAL80 repressor has only been flipped out in this cell type but persists in non‐glial *mir‐8* expressing cells (Supplementary Figure 9). For the sake of simplicity, we refer to this combination as the *mir‐8* ^*cxg*^ driver.

### 2.1 DNA constructs

For *UAS‐ClC‐a* and *UAS‐CLCN2* transgenes, we used the Gateway cloning system (Invitrogene) and cloned their respective cDNAs, to which a 3xFLAG tag had been previously added in the C‐terminus, into the ΦC31 integrase compatible pBID‐UASC‐G plasmid (Addgene plasmid # 35202, a gift from Brian McCabe [J. W. Wang, Beck, & McCabe, 2012]). The FLAG tag does not alter the electrophysiological properties of these channels. For the *ClC‐a* construct, we used the isoform C (a gift from P. Cid) since its electrophysiological properties have already been studied in *Xenopus* oocytes and HEK‐293 cells (Flores, Niemeyer, Sepúlveda, & Cid, 2009; Jeworutzki et al., 2012), and it is known to be expressed in *Drosophila* head and body (Flores et al., 2009). The final constructs were injected into the *attp40* (25C6) landing site on the second chromosome.

### 2.2 Immunohistochemistry

Fly brains were dissected in Schneider medium and fixed in 4% PFA in PBL (75 mM lysine, 37 mM sodium phosphate buffer, pH 7.4) for 25 min. After fixation, the tissue was washed in PBS with 0.5%Triton‐X‐100 (PBST) and blocked with PBST with 10% normal goat serum. Primary and secondary antibody incubations were performed in PBST and 10% normal goat serum, typically overnight at 4°C. The following primary antibodies were used for immunohistochemistry: mouse anti‐Chaoptin (1:50, 24B10, Developmental Studies Hybridoma Bank, DSHB), rat anti‐DE‐cadherin (1:50, DCAD2, DSHB), mouse anti‐Repo (1:50, 8D12, DSHB), rabbit anti‐ClC‐a (1:100 this study, see Supplementary Information, and 1:100 a gift from J. Dow), guinea pig anti‐Deadpan (1:2000, gift from A. Carmena), rat anti‐Lethal of Scute (1:5000, gift from A. Brand), rabbit anti‐Mira (1:500, gift from C. González), chicken anti‐GFP (1:800, ab13970, Abcam, Cambridge, UK), rabbit anti‐RFP (1:200, 632,496, Clontech, Mountain View, CA), mouse anti‐β‐galactosidase (1:1000, Z3783, Promega, Madison, WI), rabbit anti‐β‐galactosidase (1:1000, 0855976, Cappel, Malvern, PA), rabbit anti‐Dcp‐1 (1:200, Asp216, Cell Signaling Technology, Danvers, MA), mouse anti‐Prospero (1:10, MR1A, DSHB), mouse anti‐Cut (1:250, 2B10, DSHB) and mouse anti‐Boss (1:100, gift from H. Kremer). Alexa Fluor 488, 568, and 647 secondary antibodies raised in rabbit, mouse, rat, guinea pig, or chicken (Life Technologies, Carlsbad, CA) were used at 1:250 concentration. Nuclei were labeled using TOPRO‐3 (1:1000, T3605, Life Technologies). Brains were mounted for confocal microscopy in Vectashield (Vector Laboratories, Burlingame, CA). Samples were analyzed with Leica TCS SPE and Zeiss LSM880 confocal microscopes.

### 2.3 Photoreceptor phenotype classification

We classified brains as having a strong, medium, or weak photoreceptor phenotype based on the OL that out of the two had the most severe phenotype. If not the same, the two OLs tended to be in consecutive categories (i.e., strong/medium, medium/weak, weak/no phenotype). For experiments involving photoreceptor phenotypes, we always analyzed at least 17 brains.

### 2.4 Measurements and quantifications

To assess adult OL and CB size, we took two confocal images of each brain in the appropriate orientations to measure the antero‐posterior and dorso‐ventral axis of each structure at their widest part. We multiplied those two measurements to obtain a relative value in arbitrary units (a.u). The number of brains analyzed ranged from 6 to 44 for OL (to obtain fully independent measurements, only one OL per brain was quantified), and from 3 to 22 for CB when assessing phenotype (Figures 2c,l,m and 4h,i). In rescue experiments (Figures 2d and 4j,k), the number of brains analyzed ranged from 12 to 32.

To assess brain size at different larval stages (Figure 4g), the diameter of one larval brain hemisphere per animal was measured in the antero‐posterior axis. The *n* ranged from 23 to 37 animals analyzed.

To measure neuroepithelia volume, the tissue was stained with anti‐E‐cadherin antibody and manually segmented using the “SURFACE” tool included in Imaris software. This tool provides the volume in μm^3^ of the surfaces generated (Figure 4b). The *n* for this experiment was between eight and nine brains.

To quantify the number of cells in OPC, IPC, and CB neuroblast clones (Figure 4d,e), we performed manual counting in confocal stacks. Cells in the clone were identified as TOPRO+ nuclei surrounded by labeled membrane. We counted as many clones as possible per brain provided that they were identifiable as individual clones. The number of clones analyzed was 52 (control) and 31(mutant) in the OPC and 18 (control and mutant) in the IPC, and the number of type I neuroblast clones was 39 (control) and 22 (mutant).

To assess cell death in developing OLs (Figure 4f), we manually counted Dcp‐1^+^/TOPRO ^+^ puncta per brain hemisphere. This value was divided by the hemisphere volume obtained through manual segmentation of the structure and using the “SURFACE” tool included in Imaris software. The *n* for this experiment was six brains.

To quantify the subset of medulla glia among glial cells in the chiasm region at different stages, we manually counted ClC‐a^+^/Repo^+^ nuclei (Figure 5m). The *n* for this experiment was five brains.

To quantify the DL1 lineages nuclear green G‐TRACE signal was manually counted in confocal stacks and glial cells identified by coexpression of the glial marker Repo (Figure 6g,h). The *n* for this quantification was between 11–13 clones for the control and 9–10 clones for the mutant.

To quantify mature INPs in the DL1 and DL2 lineages (Figure 6l), we manually counted Dpn^+^ positive nuclei surrounded by tdtom^+^ membranes of the *R9D11‐tdtomato* marker. To differentiate DL1 from DL2, we used *gcm‐lacZ*, which specifically labels the DL2 lineage (Supplementary Figure 10). The *n* for this experiment was between 11 and 12 brains.

To quantify the number of total glial cells in the future chiasm region in mid L3 (Figure 8b), we manually counted Repo^+^ nuclei in confocal stacks. The following criteria were used to limit the area where we performed the counting: distally, we avoided counting flattened nuclei characteristic of surface glia and big round nuclei characteristic of surface‐associated cortex glia; the proximal limit was set the signal gap generated by the presence of the IPC. The *n* for this experiment was between 5 and 8 brains.

### 2.5 Image processing

Fiji or Imaris 8.0 (*Bitplane*, South Windsor, CT) were used to process confocal data as specified in figure legends. Figures were assembled using Adobe Illustrator (Adobe, San Jose, CA).

### 2.6 Statistics

Statistical analysis was carried out using Prism 6 (GraphPad Software Inc, San Diego, CA). When data did not follow a normal distribution or resulted from a previous mathematical computation (i.e., ratio to volume), we used nonparametric tests. For group comparisons, we used Kruskal‐Wallis followed by planned pairwise comparisons with the Mann–Whitney post hoc test to obtain *P*‐values (Figures 2c,d,l,m; 4b,h,i; 5m; and 8b). For pairwise comparisons, we used Mann–Whitney's test (Figures 4d‐f,j,k and 6g,h,l; comparison of control and mutant DL1 and comparison of control and mutant DL2). For paired comparisons, we used the Wilcoxon matched‐pairs squad rank test (Figure 6l, DL1 to DL2 comparison inside controls and inside mutants).

Data are shown in box plots where the median is given between the first and third quartiles. Whiskers represent the maximum and minimum values of the data. When all data in the analysis were suitable for a parametric analysis, we performed one‐way ANOVA followed by Turkey's post hoc test to obtain *P*‐values (Figure 4g). *P*‐values for pairwise comparisons relevant to our biological inquiry are shown in the bar graph. Data is represented as the mean, and error bars show standard deviation.

To evaluate the statistical significance of enhancements in qualitatively categorized photoreceptor phenotypes (i.e., strong, medium, weak, no phenotype) (Figure 7l,m), we performed a Chi‐squared test of independence between phenotype categories and genotypes to obtain *P*‐values.

## 3 RESULTS

### 3.1 *ClC‐a* is expressed in various glial types in the developing brain: Surface‐associated cortex glia, cortex glia and ensheathing glia

**Figure 1:**
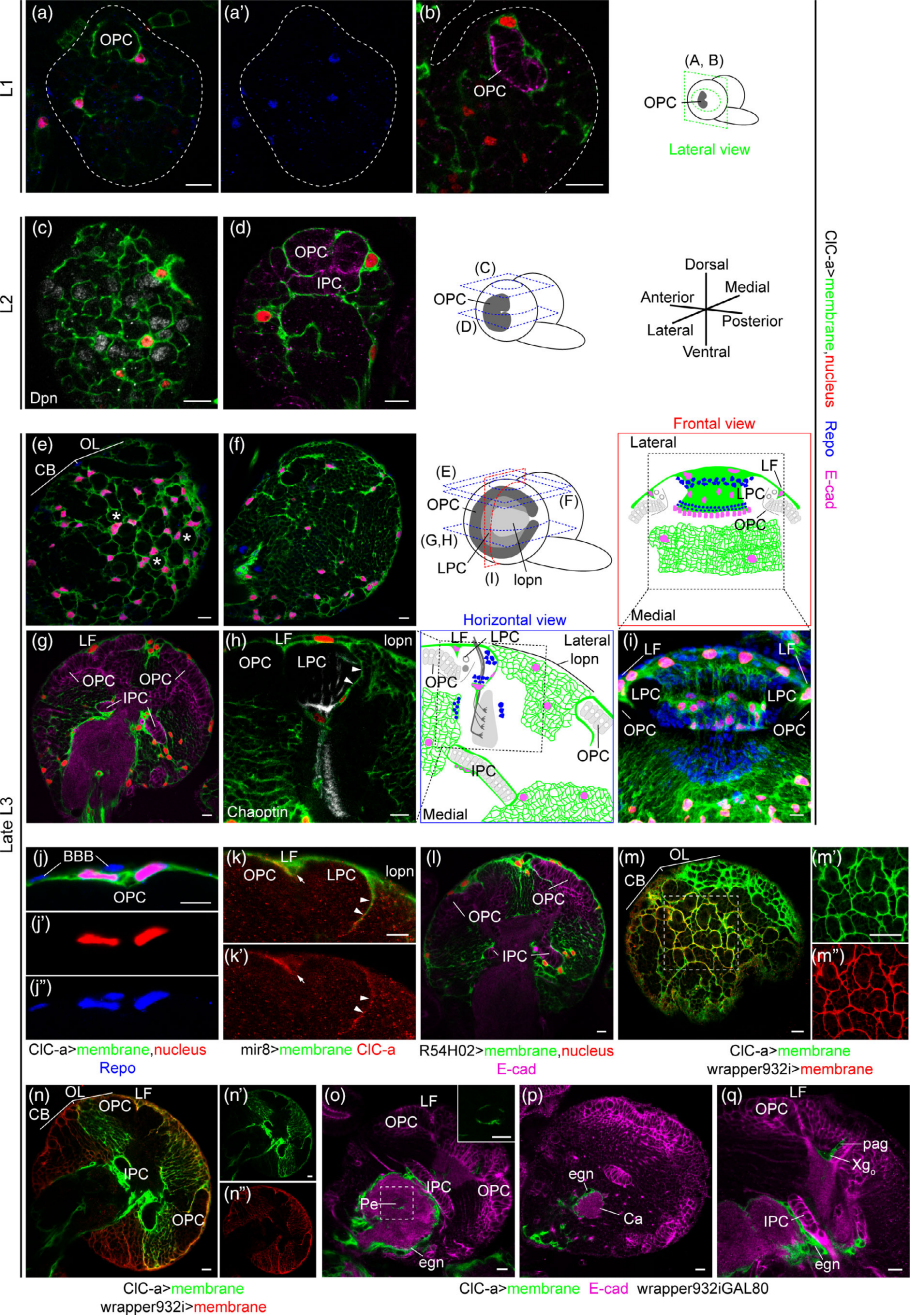
*ClC‐a *is expressed in cortex glia and ensheathing glia during brain development. (a–i) Analysis of *ClC‐a* expression in the developing brain. Brain illustrations show the orientation of imaging planes for the indicated panels at different larval stages. *ClC‐a* specific GAL4 driver (*ClC‐a‐GAL4)* was used to label cellular membranes (green) and nuclei (red) of ClC‐a^+^ cells. Glial nuclei were labeled with anti‐Repo antibody (blue). Anti‐E‐cadherin (E‐cad, magenta) was used to identify neuroepithelial cells. Neuroblasts and photoreceptors were labeled with anti‐Deadpan (Dpn, gray) and anti‐Chaoptin (gray), respectively. (a, b) Lateral view of L1 brain hemispheres outlined with a dashed line. a' shows Repo staining from (a). (c, d) Horizontal views of L2 brain hemispheres. (e, f) Horizontal views at the surface of late L3 brain hemispheres. Asterisks in (e) mark examples of cortex glia chambers. (g) Horizontal view through the middle of the L3 brain hemisphere. The lamina furrow (LF) is the indentation where OPC gives rise to LPCs in the lateral side. (h) Horizontal view at the same level as G, showing the region demarcated by the dashed box in the schematic on the right. Photoreceptors enter the brain through the LPC region. (i) Frontal view of a volume‐rendering 3D reconstruction of the OL corresponding to the region demarcated by the dashed square in the schematic at the top. The membranes of ClC‐a^+^ glial nuclei created a barrier that separated the developing lamina and the lopn. (j–q) Identification of ClC‐a^+^ glial types. (j–k) Characterization of ClC‐a^+^ cells on the OPC. (j) Analysis of ClC‐a^+^ nuclear position with respect to the BBB. (k) Colocalization analysis of *ClC‐a* protein with *mir‐8*. Expression in LF is marked by an arrow and expression between the LPC and the lopn is marked by arrowheads. (l–n) Confirmation of *ClC‐a* expression in cortex glia. (l) Membrane and nuclear patterns obtained with the *wrapper* GAL4 driver (*R54H02‐GAL4)* For similarity to *ClC‐a‐GAL4* generated patterns, compare to panel G. (m–n) Colocalization study of membrane patterns generated by the *ClC‐a‐GAL4* driver and a *LexA* driver version of the cortex glia *wrapper* driver (*wrapper932i‐LexA*) using *UAS* and *lexAop* fluorescent reporters. (m) Horizontal view of the brain surface. To visualize *ClC‐a* expression (green) in the CB (dashed region of interest), gain had to be elevated, with the consequent saturation of expression in the OL. M’ and M” show *ClC‐a* (green) and *wrapper* expression (red), respectively, from the region of interest in (m). (n) Deeper section into the hemisphere imaged with gain conditions to analyze OL colocalization; thus, the *ClC‐a* signal (green) in the CB is very low. (n') and (n'') show *ClC‐a* (green) and *wrapper* (red) membrane signals. (o–q) Identification of noncortex glia ClC‐a^+^ cells as ensheathing glia. (o) *ClC‐a* is expressed in neuropil‐ensheathing glia (eng) surrounding CB neuropils, and tract‐ensheathing glia wrapping the mushroom body peduncle (Pe, inset). (p) *ClC‐a* is expressed in glia surrounding the mushroom body calyx. (q) *ClC‐a* is expressed in palisade glia and in the outer chiasm glia (Xg_o_), which wraps photoreceptor axons in their transition from the lamina to the medulla neuropils. Scale bars represent 10 μm. (See also Supplementary Figures 1 and 2). OPC, outer proliferation center; IPC, inner proliferation center; OL, optic lobe; CB, central brain; LF, lamina furrow; LPC, lamina precursor cells; lopn, lobula plate neurons; BBB, blood brain barrier; Pe, peduncle; egn, neuropil‐ensheathing glia; Ca, calyx; Xg_o_, outer chiasm glia; pag, palisade glia

To characterize *ClC‐a* expression in the developing brain at different larval stages (first instar larva: L1; second instar larva: L2; and third instar larva: L3), we used reporter lines and antibodies. One of these reporter lines expresses GAL4 under the *ClC‐a* endogenous regulatory sequences (see below) and recapitulates the previously reported *ClC‐a* expression pattern in Malpighian tubule stellate cells observed with an antibody against ClC‐a (this study and Cabrero et al., 2014) and a ClC‐a protein trap (ClC‐a‐GFP) (Supplementary Information and Supplementary Figure 1A‐C). To visualize and monitor *ClC‐a* expressing cells throughout development, we combined the *ClC‐a‐GAL4* line with UAS transgenes that outlined the membrane and labeled the nucleus. *ClC‐a* expression was detected in L1 brains on membranes in contact with the developing OPC neuroepithelium (Figure 1a, b). Colocalization of the nuclear RFP signal with the pan glial nuclear marker Repo indicated that *ClC‐a* was expressed in a subset of glial cells (Figure 1a, a'). In L2 brains, glial membranes started encasing CB neuroblasts (Figure 1c), and more membrane processes were observed deeper in the brain (Figure 1d). By late L3, the number of ClC‐a^+^ glial nuclei in the CB had greatly increased and their glial membranes confined neuroblasts and their lineages in chambers (Figure 1e, Supplementary Figure 1J‐K). A slightly deeper section showed ClC‐a^+^ glial processes forming a smaller mesh (Figure 1f) which ensheathed mature neurons. In the OL, neuroblasts and progenitors were still being produced by the OPC and IPC, which continued to be surrounded by ClC‐a^+^ glial membranes (Figure 1g). We also observed a glial process between the developing lamina (i.e., lamina precursor cells or LPC) and the adjacent region containing lobula plate neurons (lopn) (Figure 1h), establishing a boundary between these two regions, which are innervated by neurons of different origin (i.e., photoreceptors generated in the eye disc and innervating the OL through the LPC area, and distal neurons generated from the distal‐IPC, a region of the IPC) (Figure 1i). Similar expression patterns were observed with anti‐ClC‐a antibodies and the ClC‐a protein trap, confirming the specificity of the *ClC‐a‐GAL4* driver line in the brain (Supplementary Figure 1D‐I).

We next aimed to identify which types of glial cells expressed *ClC‐a*, using cell‐type‐specific markers and nuclei position. We found that superficial *ClC‐a* ^+^ nuclei on top of the OPC neuroepithelium corresponded to a subtype of cortex glia called surface‐associated cortex glia (Morante et al., 2013), which lie beneath perineural and subperineural glia (Figure 1j). miRNA *mir‐8* (Karres, Hilgers, Carrera, Treisman, & Cohen, 2007), a marker for this subtype of cortex glia (Morante et al., 2013), colocalized with ClC‐a protein in cells covering the OPC and the process separating the LPC from the lopn (Figure 1k). Additional experiments indicated that ClC‐a^+^ nuclei present on the surface of the CB and in cortical areas belonged to cortex glia. The membrane and nuclear patterns of ClC‐a^+^ cells were consistent with the nuclear patterns and the membrane scaffold, also known as the trophospongium (Hoyle, Williams, & Phillips, 1986), observed with the recently described cortex glia driver *wrapper* (Coutinho‐Budd et al., 2017) (compare Figure 1g with Figure 1l). In fact, there was extensive colocalization between ClC‐a^+^ and wrapper^+^ membranes in the CB and OL (Figure 1m,n), including surface‐associated cortex glia on the OPC (Figure 1n,n'').

In order to assess the presence of glial types other than surface‐associated cortex glia and cortex glia, we used an intersectional strategy whereby only ClC‐a^+^/wrapper^−^ cells (i.e., noncortex glia cells) were labeled. This revealed that *ClC‐a* was also expressed in different subtypes of ensheathing glia such as neuropil‐ and tract‐ensheathing glia. *ClC‐a* was expressed in neuropil‐ensheathing glia surrounding CB neuropils, including the mushroom body calyx (Figure 1o,p). For tract‐ensheathing glia, *ClC‐a* was expressed in glia around the mushroom body peduncle (Figure 1o), and in the OL in the outer (Xg_o_) (Figure 1q) and inner (Xg_i_) (Supplementary Figure 2E‐G) chiasm glia, cell types that wrap axonal tracts between the lamina and medulla, and the medulla and lobula complex, respectively. A detailed developmental analysis revealed expression in other glial cells in the OL, as well as in the VNC and peripheral nervous system (Supplementary Figure 2). Most of the ClC‐a^+^ glial types observed in the late L3 stage persisted in the adult (Supplementary Figure 2G).

With regards to ClC‐a subcellular distribution, similar to the vertebrate channel and as previously described in stellate cells (Cabrero et al., 2014), the channel is localized in the plasma membrane of glia as shown by co‐localization of the antibody signal with a membrane marker (Figure 1k).

Together, these data indicate that *ClC‐a* is already expressed in early development in the plasma membrane of surface‐associated cortex glia and cortex glia cells, which are in direct contact with and wrap proliferative tissues such as the neuroepithelia of the OL (OPC, IPC) and neuroblasts in the CB, forming part of the niche. *ClC‐a* is also expressed in different types of ensheathing glia whose processes contribute to compartmentalization of the brain by demarcating different neuropils and neuronal tracts.

### 3.2 MiMIC insertions in the *ClC‐a* locus generate strong loss‐of‐function alleles

**Figure 2:**
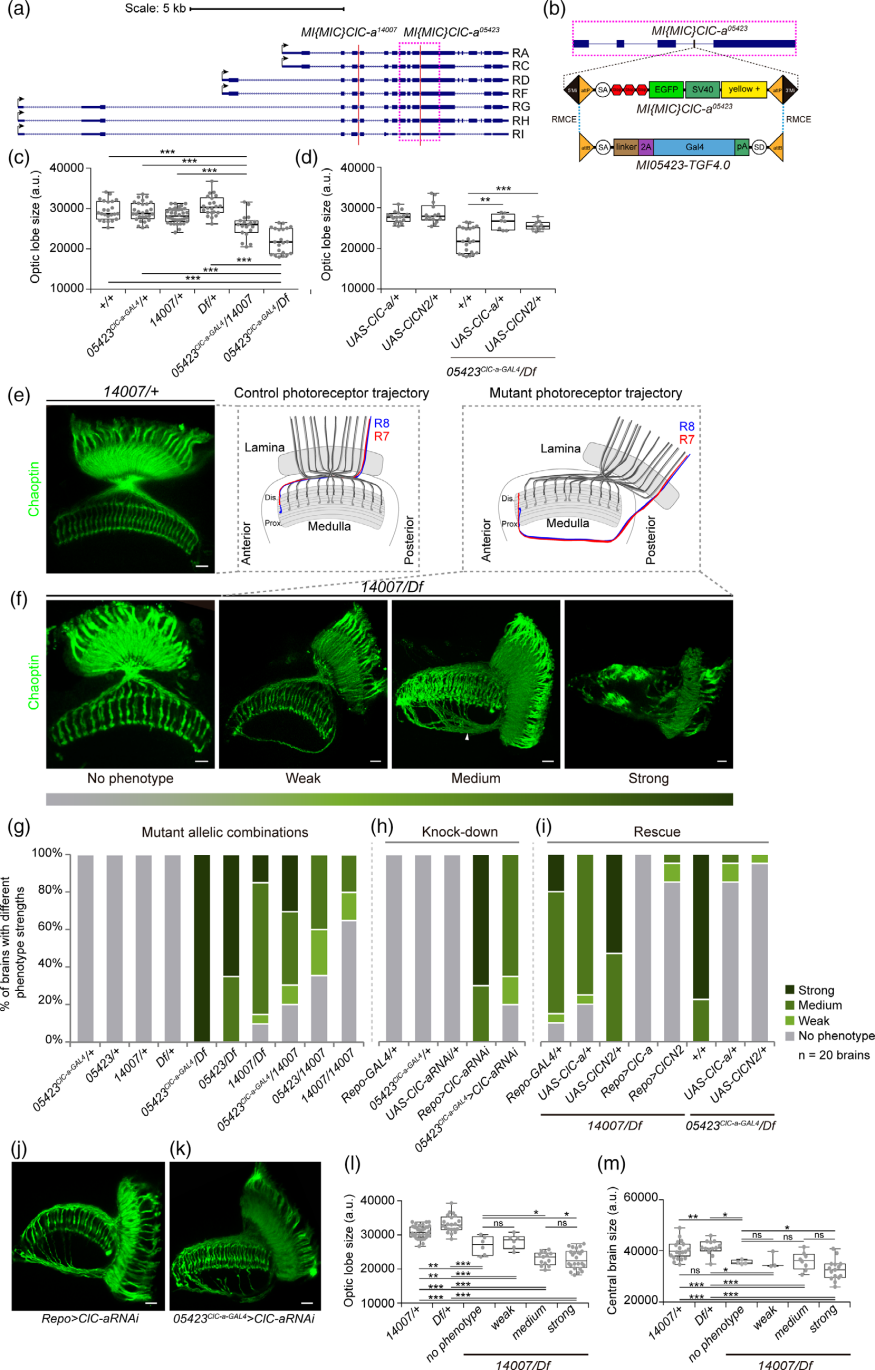
*ClC‐a *mutants have smaller brains and photoreceptor guidance defects. (a) Schematic of *ClC‐a* transcripts in the *ClC‐a* locus and the insertion location of *Mi(MIC)ClC‐a* ^*05423*^ and *Mi(MIC)ClC‐a* ^*14007*^ transposons. (b) Magnification of the pink dashed box around *Mi(MIC)ClC‐a* ^*05423*^ in (a). The original *Mi(MIC)* transposon cassette contains a splice acceptor followed by stop codons in all reading frames, followed by the EGFP coding sequence with a polyadenylation signal. When inserted in an intron between coding exons in the orientation of gene transcription, use of the transposon's splice acceptor generates truncated transcripts. The Trojan‐GAL4 cassette swapped with RMCE to generate *05423* ^*ClC‐a‐GAL4*^ contains a splice acceptor that ensures the T2A‐GAL4 open reading frame is included in the mRNA of the *ClC‐a* gene. The T2A sequence promotes separate translation of GAL4. (c, d) Quantification of OL size in arbitrary units. (c) Comparison of OL size of two *ClC‐a* mutant allelic combinations, *05423* ^*ClC‐a‐GAL4*^ */14007* and *05423* ^*ClC‐a‐GAL4*^ */Df*, and their respective controls. (d) Comparison of OL size of *05423* ^*ClC‐a‐GAL4*^ */Df* and mutant brains in which *ClC‐a (UAS‐ClC‐a)* or rat *CLCN2* (*UAS‐CLCN2*) mRNAs were expressed in glia. (e–i) Characterization of photoreceptor guidance defects. (e) Confocal section of an adult OL of a heterozygous control animal (*14007/+*), showing the wild type photoreceptor array stained with anti‐Chaoptin (24B10, green). The schematic shows the trajectory of R7 and R8 photoreceptor axons. (f) Confocal images of adult OLs from the *14*,*007/Df* mutant allelic combination classified according to phenotype severity. For the sake of simplicity, the schematic depicts the altered trajectory of R7 and R8 axons of a single ommatidium. To show the complete trajectory of misguided photoreceptors, images for the weak and medium phenotypes are Z‐projections of confocal stacks. (g–i) Photoreceptor phenotype analysis for different experiments. Phenotype penetrance and expressivity for each condition is depicted as the percentage of brains with no phenotype, weak, medium, and strong phenotypes (see Material and Methods). Heterozygous controls in (g) and (h) show no phenotype. (g) Classification of *ClC‐a* mutant allelic combinations according to strength of their penetrance and expressivity. (h) Analysis of glia‐specific knock down of *ClC‐a* using RNAi. (i) Glia‐specific rescue experiment using *ClC‐a* and rat *CLCN2* mRNAs in two allelic combinations, *14*,*007/Df* and *05423* ^*ClC‐a‐GAL4*^ */Df*. Examples of medium photoreceptor phenotypes generated by *ClC‐a* RNAi expression under the glial Repo‐GAL4 driver (j) and ClC‐a‐GAL4 (k). (l, m) Brain measurements of control, and mutant animals without photoreceptor defects or different degrees of photoreceptor phenotypes. (l) Comparisons of optic lobe measurements. (m) Comparisons of central brain measurements. Scale bars represent 10 μm. n.s. > .05, * *p* < .05, ** *p* < .01, *** *p* < .001. (See also Supplementary Figures [Link], [Link] and 5)

To explore the role of *ClC‐a* in glia, we characterized a set of *Minos*‐mediated integration cassette [Mi(MIC)] insertions in the *ClC‐a* locus (Figure 2a). This transposon contains a gene trap cassette that leads to the formation of truncated transcripts (Venken et al., 2011) (Figure 2b). We focused on *Mi(MIC)ClC‐a* ^*05423*^ and *Mi(MIC)ClC‐a* ^*14007*^ alleles (from now on referred to as *05423* and *14*,*007*), since their insertion, sites were predicted to interrupt all isoforms of the *ClC‐a* gene. The *ClC‐a‐GAL4* line we used was derived from *Mi(MIC)ClC‐a* ^*05423*^ by the Gene Disruption Project (Nagarkar‐Jaiswal et al., 2015; Nagarkar‐Jaiswal et al., 2015) through recombinase‐mediated cassette exchange (RMCE) replacement of the MiMIC gene trap cassette for a GAL4 cassette (Diao et al., 2015) (Figure 2b). Hence, a mutant allele is generated that expresses GAL4 under the control of *ClC‐a* regulatory sequences. From now on, we will refer to it as *05423* ^*ClC‐a‐GAL4*^. To classify these insertions in an allelic series and identify the strongest allelic combinations, we crossed them to the *Df(3R)PS2* deficiency (*Df*) and among themselves, and used viability as the readout. For example, the very few *05423/Df* animals that emerged from the pupal case did so 48 hrs later than heterozygote controls, and remained immobile on the food before dying shortly after eclosure. The proportion of *05423* ^*ClC‐a‐GAL4*^ */Df* emerging escapers was even lower than the one observed for *05423/Df*. Escapers of the *14*,*007/Df* allelic combination emerged with a delay of around 24 hrs, but compared to *05423/Df* were more abundant and healthier, all emerging from the pupal case. Similar to *14*,*007/Df* animals, *05423* ^*ClC‐a‐GAL4*^ */14007* also had a 24‐hr developmental delay with respect to controls. Thus, based on these viability observations we could order by strength the analyzed allelic combinations in the following sequence: *05423* ^*ClC‐a‐GAL4*^ */Df > 05423/Df > 14*,*007/Df = 05423* ^*ClC‐a‐GAL4*^ */14007 > 05423/14007 > 14*,*007/14007*. This analysis also allowed us to identify the best allelic combination to use for phenotype analysis and to correlate control and mutant developmental time points (Supplementary Information). Since it is difficult to obtain *05423* ^*ClC‐a‐GAL4*^ */Df* or *05423/Df* animals in sufficient numbers, we mainly used *14*,*007/Df* and *05423* ^*ClC‐a‐GAL4*^ */14007* flies in our experiments. These two allelic combinations behave in a very similar fashion and represent a good compromise in terms of phenotypic strength and mutant animal availability. In addition, the *05423* ^*ClC‐a‐GAL4*^ */14007* combination enables visualization in the mutant background of the glial cells that express *ClC‐a* in wild type.

The predicted loss‐of‐function nature of the MiMIC insertions characterized was confirmed by immunostaining and western blot. The *ClC‐a* expression pattern observed with anti‐ClC‐a antibody in wild type L3 stellate cells of the Malpighian tubules and brain was not detected in any of the mutant allelic combinations tested (Supplementary Figure 3A‐D). Western blots revealed that with a very low frequency, the splice machinery used the endogenous splice acceptor instead of the MiMIC one, and that there was a remnant, albeit very low, of wild type protein in mutants that was only detectable in immunoblots (Supplementary Figure 3E, F).

In summary, here we have characterized the first *ClC‐a* mutants, which are strong loss‐of‐function alleles.

### 3.3 Mutations in *ClC‐a* result in smaller brains with photoreceptor guidance defects

To explore the effect of *ClC‐a* mutations on brain development, we started by dissecting adult brains and searching for defects that could have a developmental origin based on *ClC‐a* expression patterns in the larval brain. The observation that *ClC‐a* was expressed in glia on proliferative tissues in the brain (i.e., neuroepithelia and neuroblasts) led us to hypothesize that mutant brains might be smaller than control ones, and to test this idea we measured OLs from control and mutant animals. We did indeed observe a reduction in OL size in mutants, which was particularly evident in *05423* ^*ClC‐a‐GAL4*^ */Df*, the strongest allelic combination, and was also present in *05423* ^*ClC‐a‐GAL4*^ */14007* (Figure 2c) and *14*,*007/Df* (Figure 4h,i).

Given that we detected *ClC‐a* expression in glial processes separating the developing lamina from the lopn and in outer chiasm glial cells (Figure 1h,k,q), we labeled photoreceptors to assess their innervation path. The compound eye of the fly is formed by some 800 units called ommatidia. Each ommatidium contains eight photoreceptors; R1‐6, which terminate in the lamina forming the lamina plexus; and R7 and R8, which extend to the medulla. As rows of ommatidia form in the eye disc, photoreceptors extend axons and sequentially innervate the OL. This forms a retinotopic map and each ommatidium in the eye generates a corresponding processing unit in the lamina and the medulla neuropils. In control adult OLs (Figure 2e, schematic), R‐cell axons from the posterior edge of the eye enter through the posterior lamina where R1‐6 stop. R7 and R8 axons traverse the outer optic chiasm and project into the anterior‐most medulla; similarly, R‐cell axons from the anterior region of the eye project into the posterior medulla. All R7 and R8 axons enter the medulla neuropil from its distal face and their projections align in a stereotyped array forming a retinotopic map (Figure 2e).

Analysis of *ClC‐a* mutant adult OLs using a pan photoreceptor marker revealed photoreceptor guidance defects. The guidance phenotypes observed could be classified into three levels of severity based on the proportion of R‐cell axons affected (Figure 2f). In brains with phenotypes classified as medium, a significant portion of posterior R‐cell axons bypassed the outer chiasm, projected along the posterior edge of the medulla neuropil turning anteriorly, and extended for variable distances before innervating the medulla neuropil from its proximal face. In many cases, this resulted in posterior misplacement of the lamina neuropil. Despite the presence of these discreet bundles of misprojected axons that originate posteriorly, the photoreceptor array was maintained and mostly regular. We classified instances of few misprojected posterior axons as weak phenotypes. Strong phenotypes were characterized by severe disruption of the photoreceptor array and a posteriorly located, disorganized lamina. Despite the difficulty in identifying discreet bundles of photoreceptor axons, distal innervation was evident. Eye disc development was normal (Supplementary Figure 4 A‐I) and these three degrees of severity showed variable penetrance and expressivity depending on the allelic combination analyzed (Figure 2c). This variability could be explained by the fact that *ClC‐a* mutants were not complete nulls. A detailed analysis of mutant photoreceptors also revealed layer selection defects for R8 and R1‐R6 neurons (Supplementary Figure 5).

In order to confirm the requirement of *ClC‐a* in glia, we performed cell‐type‐specific knock down and rescue experiments. In addition to the *ClC‐a* driver, we also used the general glial driver Repo‐GAL4 to directly support the conclusion that the channel is required in glia. Using these two drivers, *ClC‐a* knockdown by RNAi phenocopied the photoreceptor phenotypes seen in the mutant (Figure 2h,j,k). Moreover, expression with both drivers of *ClC‐a* and rat *CLCN2* cDNA rescued the photoreceptor phenotypes in whole mutant animals (Figure 2i). Although it has been suggested that pupal photoreceptors express *ClC‐a* (Ugarte et al., 2005), we did not observe this in larval, pupal, or adult stages with the antibodies (Supplementary Figure 4 J‐M) or reporters used in this study (Supplementary Figure 2D‐G). In addition, the absence of phenotype when knocking down *ClC‐a* in the eye disc or generating a full eye mutant for *ClC‐a*, together with the inability to rescue the guidance phenotype when expressing *ClC‐a* in photoreceptors (Supplementary Figure 4 N‐R), confirmed that *ClC‐a* was required in glia for photoreceptor guidance.

Remarkably, taking advantage of the *05423* ^*ClC‐a‐GAL4*^ allele, we observed a rescue of both OL size and photoreceptor guidance phenotypes in *05423* ^*ClC‐a‐GAL4*^ */Df*, the strongest allelic combination, with *ClC‐a* and *CLCN2* cDNA transgenes alike (Figure 2d,i). Together with the results obtained with Repo‐GAL4, this finding supports the idea that brain size reduction and photoreceptor guidance phenotypes in *ClC‐a* mutants are nonautonomous and dependent on chloride channel expression in glia, and that the fly and rat channels have equivalent functions.

To understand the relationship between the brain size and photoreceptor phenotypes we investigated if there was a correlation between them by measuring the CB and OL of mutant brains with different strengths of the photoreceptor phenotype. Analysis of the *14*,*007/Df* allelic combination revealed that mutant brains are significantly smaller, both at the level of OL (Figure 2l) and CB (Figure 2m), than controls regardless of the photoreceptor phenotype. Remarkably, mutant brains with no phenotype were already smaller than controls. We also observed that for the OL, brains with the medium or strong photoreceptor phenotype tend to be slightly smaller. However, despite the qualitative jump in the severity of guidance defects between the medium and strong photoreceptor phenotype classification, the OL size of these animals is not significantly different. Thus, taken together these results suggest that the brain size phenotype is independent of the photoreceptor phenotype.

### 3.4 Expression of *ClC‐a* in surface‐associated cortex glia and cortex glia is required for neuroepithelial expansion and the generation of neuroblast lineages, and is sufficient to restore brain size

**Figure 3:**
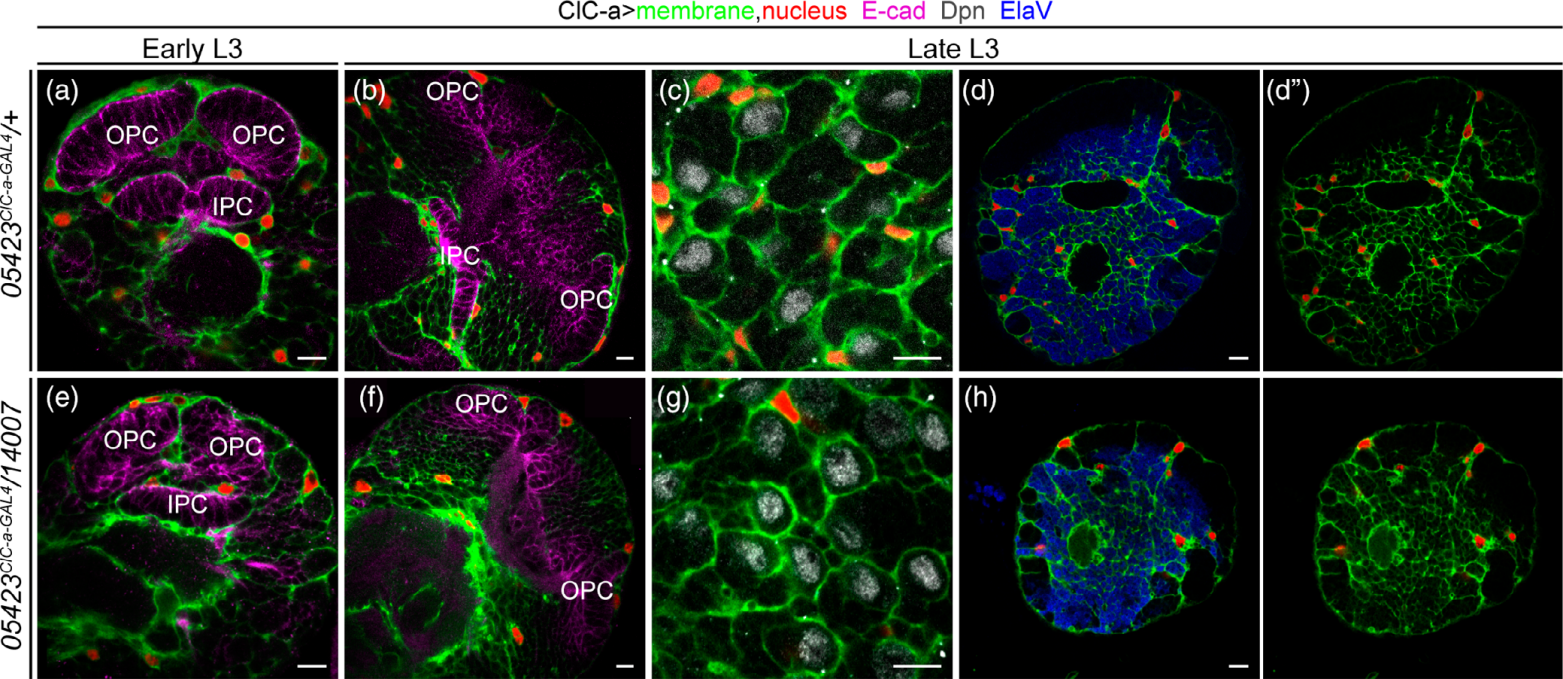
Surface‐associated cortex glia and the cortex glia membrane scaffold remain unaltered in *ClC‐a* mutant animals Analysis of surface‐associated cortex glia and the cortex glia membrane scaffold (green) and nuclear (red) distribution in control (*05423* ^*ClC‐a‐GAL4*^ */+*) and mutant (*05423* ^*ClC‐a‐GAL4*^ */14007*) brain hemispheres. Horizontal views at specified developmental times and depths are shown. View through the middle of the early (a) and late (b) L3 hemisphere of a control animal. Anti‐E‐cadherin (E‐cad, magenta) labels neuroepithelial cells. (c) View of the surface of a control brain stained with anti‐Deadpan (Dpn, gray) to visualize neuroblasts. (d, d') Slightly deeper view of the surface of a control brain stained with anti‐Elav to visualize postmitotic neurons. (e–g) and (h, h') panels are equivalent views and stainings in mutant animals (See also Supplementary Figure 6). Scale bars represent 10 μm. OPC, outer proliferation center; IPC, inner proliferation center

In order to unravel how mutations in *ClC‐a* resulted in smaller brains, we first assessed the status of glia in *ClC‐a* mutants. We used the *05423* ^*ClC‐a‐GAL4*^ */14007* allelic combination to visualize glia membranes and nuclei in the mutant background. Our analysis showed that the distribution pattern of glial cell bodies on the brain surface and deep in the cortex was similar in control and mutant animals. Although the number of nuclei/hemisphere volume ratio in the mutant was slightly reduced compared to control (Supplementary Figure 6A), importantly, the membrane scaffold appeared indistinguishable from the one observed in controls covering the whole hemisphere. As in control animals, *ClC‐a* mutant surface‐associated cortex glia and cortex glia processes were in close contact with the OPC and IPC neuroepithelia respectively (Figure 3a,b,e,f). In addition, in the OL and the CB alike, cortex glia processes formed the trophospongium. Thus, individual neuroblasts were enclosed in chambers that enlarged to adapt to their lineage expansion (Figure 3c,g), and mature neuronal cell bodies were progressively enwrapped by cortex glia processes (Figure 3d,d',h,h'). From these observations, we conclude that mutations in the channel do not result in major morphological changes in the trophospongium formed by cortex glia.

In turn, these results suggested that *ClC‐a* was instead required for the proper physiology of surface‐associated cortex glia and cortex glia. Cortex glia have been shown to be essential for neurogenesis (Dumstrei et al., 2003), and since surface‐associated cortex glia processes are tightly associated with the OPC (Morante et al., 2013) and cortex glia to the IPC, we set out to examine whether the small OLs in mutant adult brains (Figure 2c,l) were a consequence of defects in these neuroepithelia. Neuroepithelia in the OL start as sheets of cells that divide symmetrically and expand until mid L3 (Ngo et al., 2010). As they do so, they bend along the dorso‐ventral axis, creating a crescent shaped structure with the opening pointing posteriorly (Nassif, Noveen, & Hartenstein, 2003). Already in late L2, while the OPC neuroepithelium is still growing to expand the pool of prospective neuroblasts, neuroepithelium to neuroblast transition starts taking place. The lateral edge gives rise to LPC and the medial edge to neuroblasts that will produce medulla neurons and glia (B. Egger, Gold, & Brand, 2010; Boris Egger, Boone, Stevens, Brand, & Doe, 2007; Ngo et al., 2010; Orihara‐Ono, Toriya, Nakao, & Okano, 2011; Reddy, Rauskolb, & Irvine, 2010; Wang et al., 2011; Wang, Li, Zhou, Yue, & Luo, 2011; Weng, Haenfler, & Lee, 2012; Yasugi, Sugie, Umetsu, & Tabata, 2010; Yasugi, Umetsu, Murakami, Sato, & Tabata, 2008). Once neuroepithelium divisions stop and the wave of differentiation continues, the OPC neuroepithelium starts reducing in size and disappears in early pupal stages, when it is all converted into precursors and neuroblasts. A similar process takes place in the IPC, where different domains generate neuroblasts or migrating progenitors (Apitz & Salecker, 2015; Hofbauer & Campos‐Ortega, 1990) until the neuroepithelium disappears.

**Figure 4:**
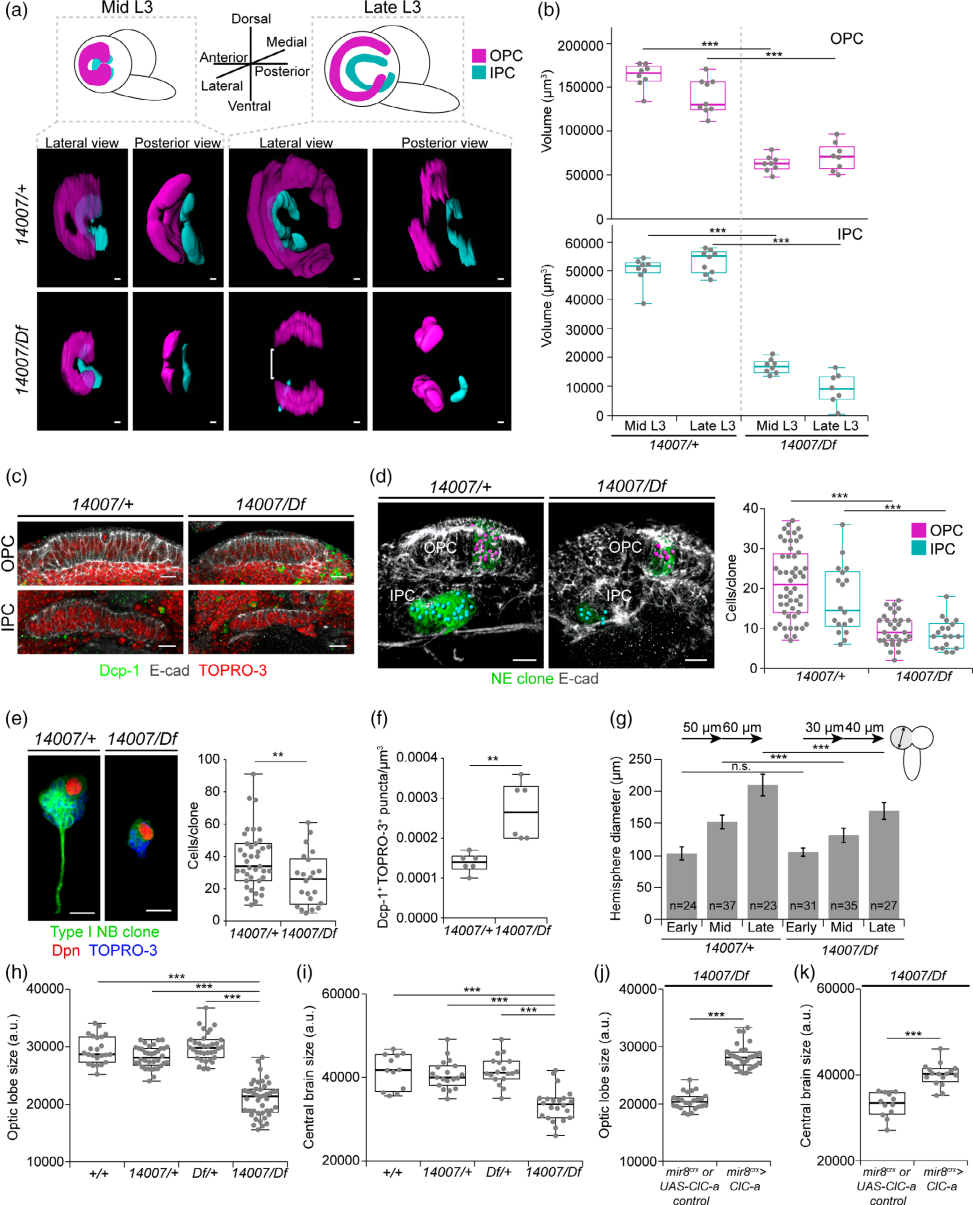
*ClC‐a* is required for neuroepithelial expansion, neuroblast lineages, as well as neuronal viability, and is sufficient to rescue brain size. (a) Images of surface‐rendering 3D reconstructions of the OPC (magenta) and IPC (cyan) shown from lateral and posterior views, in control (*14007/+*) and mutant (*14*,*007/Df)* brains. Bracket indicates the absence of the central domain of the OPC in mutant late L3 reconstructions. (b) Quantification and comparison of the volume in μm^3^ of reconstructed OPC (magenta) and IPC (cyan) of mid and late control and mutant animals. (c) Analysis of cell death in mid L3 OPC and IPC (E‐cad, gray) of control and mutant animals using anti‐Dcp‐1 staining (Dcp‐1, green) to label apoptotic cells. Nuclei (red) are labeled with TOPRO‐3. Confocal sections show that apoptotic cells in control and mutant tissue were found outside the neuroepithelial cells. (d) Images of volume‐rendering 3D reconstructions of control and mutant mid L3 OLs with mitotic clones (green) in the OPC and IPC. Anti‐E‐cadherin (E‐cad, gray) labels neuroepithelial cells. Magenta and blue spheres represent cells in OPC and IPC clones, respectively. Quantification and comparison of the number of cells per OPC and IPC clone in the control and mutant background. (e) Images of volume‐rendering 3D reconstructions of segmented mitotic clones in type I neuroblast in mid L3 control and mutant animals. The clone is labeled in green. Anti‐Dpn staining (Dpn, red) identifies the neuroblast. TOPRO‐3 labels the nuclei of cells in the clone. Quantification and comparison of the number of cells per clone in type I neuroblast clones in control and mutant animals. (f) Quantification and comparison of cell death (Dcp‐1^+^/TOPRO‐3^+^ puncta) in mid L3 brain hemispheres. (g) Graphic showing the diameter of larval hemispheres at different L3 stages in control and mutant animals. Error bars indicate standard deviation. Comparisons between control and mutant diameters at each larval stage are shown. The growth rate between larval stages in controls and mutants is indicated at the top of the graphic. Quantifications and comparisons of adult OL (h) and CB (i) size for *14*,*007/Df* animals and controls. Quantifications and comparisons of adult OL (j) and CB (k) size in cortex glia‐specific rescue experiment brains and the appropriate controls. Control brains represent genotypes for both the GAL4 driver and the UAS transgene in the mutant background since they could not be distinguished in the genetic scheme of the experiment (*mir‐8* ^*glia*^ control and *UAS‐ClC‐a* control). For surface‐associated cortex glia and cortex glia‐specific driver details, see Materials and Methods and Supplementary Figure 9. Scale bars represent 10 μm. n.s. > .05, ** p < .01, *** p < .001. (See also Supplementary Figures 7, 8 and 9). OPC, outer proliferation center; IPC, inner proliferation center

We first checked if there were differences in neuroepithelia between control and mutant animals. For this, we stained brains with the neuroepithelium marker E‐cadherin and manually segmented the tissue to generate a 3D reconstruction of these structures, which yielded information about their morphology (Figure 4a) and size (Figure 4b). In control animals in the mid L3 stage, the ends of the OPC and IPC neuroepithelia crescents were close together. In late L3, with the addition of progeny from neuroblasts, the OL was larger and neuroepithelia crescents were wider and thinner. In comparison, in mid L3 mutant animals, neuroepithelia maintained the same crescent shape as in controls but were already clearly smaller (Figure 4b). By late L3, in most cases the OPC neuroepithelium appeared as two separate dorsal and ventral domains with the central part absent. Similarly, part of the IPC was also missing (Figure 4a).

We next wondered whether the reduction in the size of the neuroepithelial sheets was due to cell death. To test this idea, we stained larval brains with an antibody against the apoptosis marker Dcp‐1 (cleaved death caspase protein‐1). Although developmental cell death was taking place generally in the brain, we did not observe apoptotic cells either in control or mutant neuroepithelial cells in mid or late L3 stages (Figure 4c). Thus, the absence of cell death in this tissue suggested that defects in neuroepithelium expansion could be responsible for the reduction in size of the OPC and IPC neuroepitheliums at mid L3, and also for the morphological defects in late L3. In the latter, the lack of neuroepithelial cells in the OPC central domain could be explained by neuroepithelium to neuroblast transition taking place in the already reduced neuroepithelium, which would result in a premature disappearance of central domain of the OPC neuroepithelium (Supplementary Figure 7) since that is where the transition starts. To examine neuroepithelium expansion defects, we carried out a clonal analysis study. With this technique, once mitotic recombination has been induced in a dividing neuroepithelial cell, its progeny is labeled, and can thus be counted. Clones were generated in the L1/L2 transition and their size was assessed 48 hrs later at mid L3. Neuroepithelia clones generated in the control background (brains where cortex glia expressed *ClC‐a*) presented a median size of 21 cells for OPC clones and 14.5 cells for IPC clones. Conversely, clones generated in the mutant background (brains where cortex glia did not express *ClC‐a*) were significantly reduced, with a median size of nine and eight cells for OPC and IPC clones, respectively (Figure 4d). Differences between control and mutant animals were also observed in an EdU labeling experiment (Supplementary Figure 8). Thus, based on these results we proposed that *ClC‐a* was necessary in surface‐associated cortex glia for neuroepithelial expansion.

Given that neuroblasts originated from the OPC and the central brain are close contact with *ClC‐a* expressing glia, we also used clonal analysis to assess how neuroblasts generated their lineages in *ClC‐a* mutants. For this analysis, we focused on neuroblasts of the CB since it allowed us to address the origin of CB size reduction in mutants. Importantly, both control and mutant animals showed the same number of neuroblasts; thus, CB size reduction in mutants was not due to a decrease in neuroblasts (Supplementary Figure 6B). Using a similar clone induction protocol as for neuroepithelial clones, the median size of type I neuroblast clones in the control background was 34 cells, whereas the median size for clones in the mutant background was reduced to 26 cells (Figure 4e). In addition, at this same mid L3 stage, we also detected more dispersed cell death in mutant than control brains (Figure 4f) in regions other than the neuroepithelia, which we had shown were death free (Figure 4c). This result is consistent with the described trophic role of cortex glia processes that wrap the cell bodies of the more mature neurons of the lineage (Coutinho‐Budd et al., 2017; Dumstrei et al., 2003; Pereanu et al., 2005; Read, 2018; Spéder & Brand, 2018), and suggests that alterations in the physiology of cortex glia in *ClC‐a* mutants affects the viability of mature neurons. Hence, although evenly distributed in the brain, we cannot rule out that some of this cell death contributes to the reduction in size of type I neuroblast clones in the *ClC‐a* mutant background.

Together, these data suggest that the lack of *ClC‐a* in surface‐associated cortex glia and cortex glia in the niche affects neuroepithelial expansion, neuroblast lineages, as well as mature neuron viability outside the niche. Consistent with both these observations, the size and growth rate of larval hemispheres was reduced in the mutant background (Figure 4g). Thus, these results are in accordance with a smaller OL (Figure 4h) and CB (Figure 4i) in adult *ClC‐a* mutant brains than in those of control flies. Importantly, expression of *ClC‐a* exclusively in surface‐associated cortex glia and cortex glia was sufficient to rescue the size of both structures in the adult (Figure 4j,k).

### 3.5 Defects are also observed in the neuroblast lineage that gives rise to ClC‐a^+^ ensheathing glia, which are necessary guideposts for photoreceptor axons innervating the medulla

**Figure 5:**
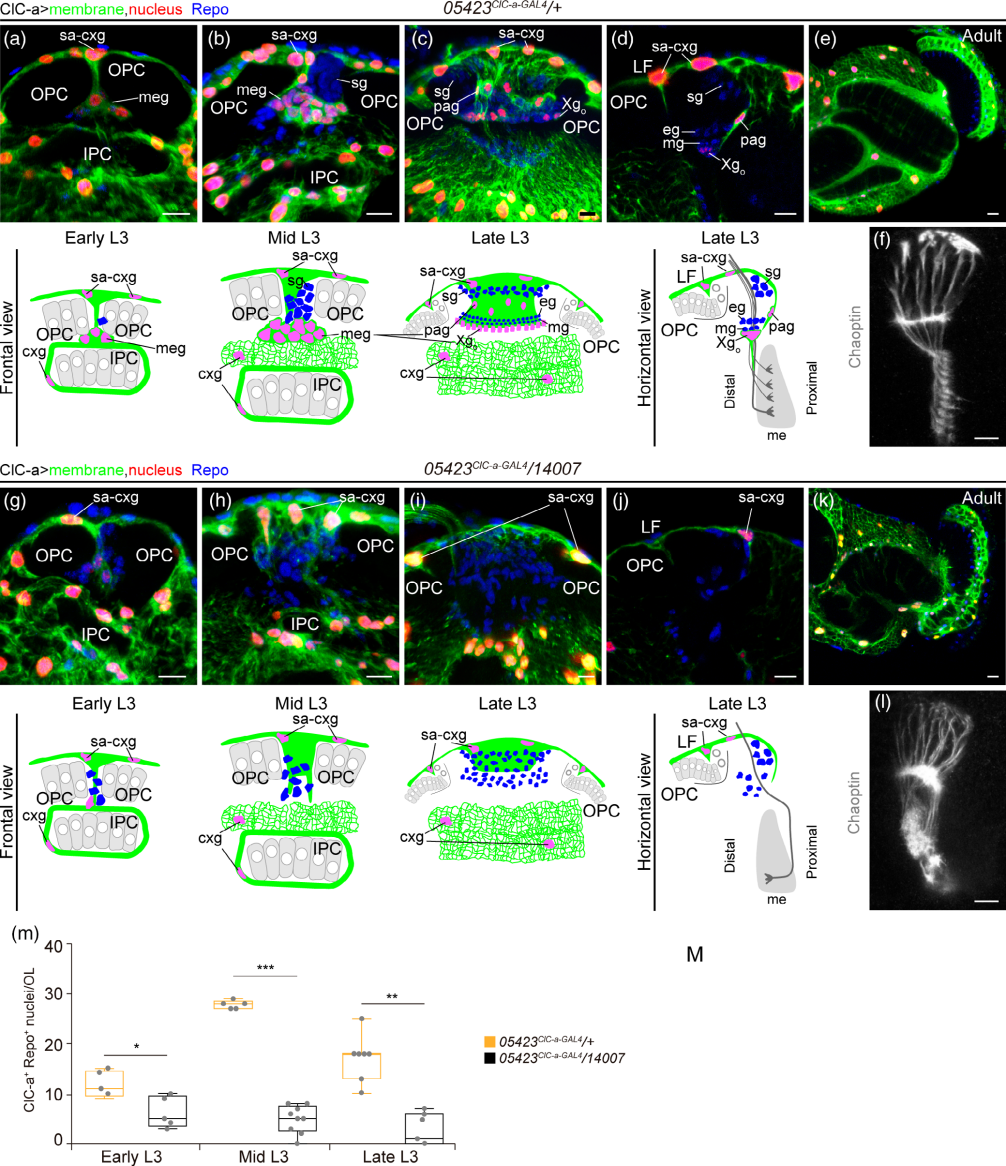
Strong reduction in a subset of ClC‐a^+^ ensheathing glial cells is observed in *ClC‐a* mutants. Developmental analysis of cells that express *ClC‐a* in the OL region in control animals (*05423* ^*ClC‐a‐GAL4*^ */+*) and those same cells in *ClC‐a* mutant animals (*05423* ^*ClC‐a‐GAL4*^ */14007*). Surface‐associated cortex glia and cortex glia membranes are shown in green and nuclei in red. All glial nuclei were labeled with anti‐Repo antibody (blue). (a–d) Images of the ClC‐a^+^ glial barrier from early to late L3 control OLs with the corresponding schematics, in frontal views (a–c) and horizontal view (d). Volume‐rendering 3D reconstructions showing the ClC‐a^+^ medulla glia population in early (a) and mid (b) L3, and its division into pag and Xg_o_ in late L3 (c). (d) Confocal section. The schematic includes photoreceptors, not labeled in (d) but shown in (f). (e) *ClC‐a* expression pattern in the adult OL. The inner and outer chiasms are correctly formed. (f) Photoreceptor axons (Chaoptin, gray) in late L3 OLs. For their position relative to glia, see the horizontal view schematic. Images showing which of the glial cells that would normally express *ClC‐a* in control OLs are still present in *ClC‐a* mutant OLs from early to late L3 larval stages, with the corresponding schematics, in frontal views (g–i) and horizontal view (j). (G–i) Volume‐rendering 3D reconstructions. (j) Confocal section. The schematic shows the aberrant trajectory that some photoreceptor axons can take in (l). (k) *ClC‐a* expression pattern in the mutant adult OL. The inner and outer chiasms are defective. (l) Photoreceptor axons (Chaoptin, gray) in late L3 mutant OLs. For their position relative to glia, see the horizontal view schematic. (m) Quantification and comparison of ClC‐a^+^/Repo^+^ nuclei in the OL region. Scale bars represent 10 μm. * *p* < .05, ** p < .01, *** p < .001 (See also Supplementary Figure 10). OPC, outer proliferation center; IPC, inner proliferation center; sa‐cxg, surface‐associated cortex glia; cxg, cortex glia; sg, satellite glia; meg, medulla glia; pag, palisade glia; Xg_o_, outer chiasm glia; eg, epithelial glia; mg, marginal glia; me, medulla

In an attempt to understand how the nonautonomous photoreceptor guidance phenotype is related to *ClC‐a* expression in the OL, we performed a detailed developmental expression analysis in the region where photoreceptor innervation takes place. In control L2 brains, horizontal views showed that the OPC and IPC were still juxtaposed and that ClC‐a^+^ cell bodies were present on the surface of the brain and in the CB (Figure 1d). In L3 frontal views, we observed that a population of glia, which preceded the arrival of photoreceptor axons in the lamina (Dearborn, 2004; Perez & Steller, 1996), progressively positioned amid the expanding region between the OPC and IPC during the early to mid L3 stages (Figure 5a,b) and ended up forming a barrier between the developing lamina and the lopn (Fan et al., 2005). Taking advantage of the recent availability of markers for different glial cell types we have been able to accurately characterize the ClC‐a^+^ cells forming the barrier. The aforementioned glial population could be divided into two sets of nuclei, the ClC‐a^−^ nuclei of satellite glia (Supplementary Figure 10A, B) and a population of ClC‐a^+^ nuclei, with lower expression than cortex glia, known as medulla glial cells (Chotard & Salecker, 2008) (Figure 5b). From this seemingly homogenous mid L3 medulla glia population (ClC‐a^+^), two cell types could be distinguished in late L3 brains in frontal (Figure 5c) and horizontal views (Figure 5d, Supplementary Figure 10C): the Xg_o_ and a glial type that had been classified before as satellite glia based on position (Fan et al., 2005). Based on cell type specific drivers and co‐localization experiments (Supplementary Figure 10A‐C) we concluded that the latter glial type did not belong to the satellite glia population and we named it palisade glia (pag). They were positioned on the same plane as the cortex glia projection and the Xg_o_, forming a continuous ClC‐a^+^ glial barrier between the developing lamina and the lopn. We do not know if pag persist or which type they are in the adult (Figure 5e). Xg_o_ are considered tract‐ensheathing glia, and one glial cell enwraps an average of 15 lamina‐medulla fiber tracts (Kremer, Jung, Batelli, Rubin, & Gaul, 2017). Two independent studies have shown that Xg_o_ and Xg_i_ originate from the type II DL1 neuroblast lineage and migrate to the OL (Ren, Awasaki, Wang, Huang, & Lee, 2018; Viktorin, Riebli, & Reichert, 2013). We repeated DL1 lineage‐tracing experiments and observed that progeny from the DL1 populated the OL following the same temporal pattern as ClC‐a^+^ medulla glial cells (Supplementary Figure 10D‐F). Hence, our data support the idea that medulla glial cells are DL1 progeny that differentiate into the newly described pag and Xg_o_. Quantification of medulla glia in control brains showed that their numbers increased from early to mid L3 and then dropped at late L3 (Figure 5m, Supplementary Figure 10G, H). In mutant brains, however, we observed a striking reduction in the number of medulla glial cells in mid and late L3 stages (Figure 5g‐j,m). Given that no glial apoptosis was observed in the region (Supplementary Figure 10I, J), this result indicated that only very few medulla glial cells reached the OL in *ClC‐a* mutants.

**Figure 6:**
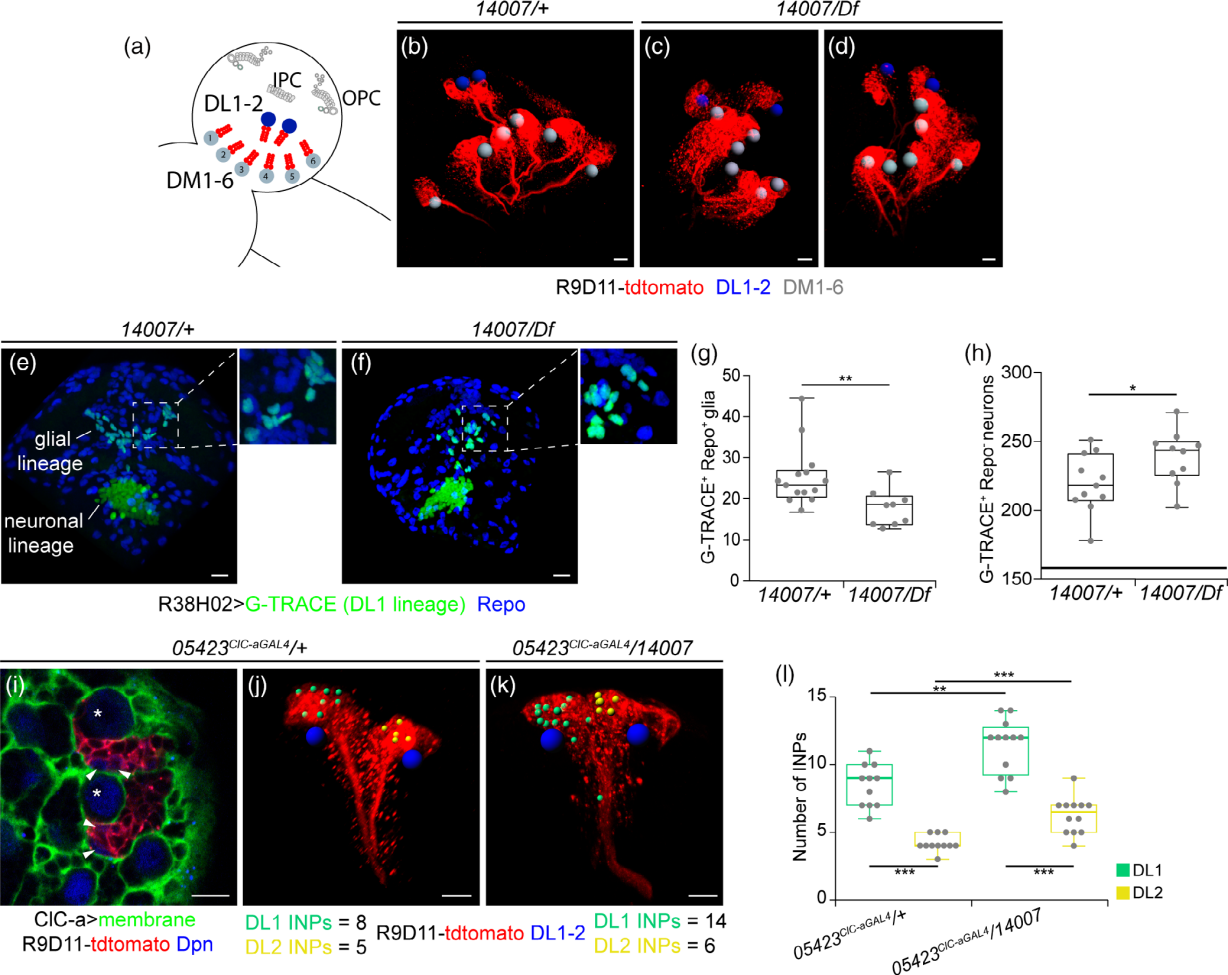
Defects in the type II DL1 neuroblast lineage are observed in *ClC‐a* mutants. (a) Schematic showing the relative position of DM and DL lineages. Volume‐rendering 3D reconstructions of late L3 control (*14*,*007/+*; b) and mutant (*14*,*007/Df*; c, d) brain hemispheres showing type II lineages labeled with the R9D11‐tdtomato (red). Gray and blue spheres mark the position of the DM and DL neuroblasts, respectively. Frontal views of a volume‐rendering 3D reconstruction of G‐TRACE (green) DL1 lineages in control (e) and mutant (f) mid L3 brains. Inserts show a magnification of the glial lineages to highlight colocalization of G‐TRACE with the glial marker Repo (blue). Quantification and comparison of the glial (g) and neuronal (h) sublineages in control and mutant animals. (i) Confocal image showing the DL1/2 cluster lineages (red), the neuroblast (asterisk), and mature INPs (arrowheads) labeled with anti‐Deadpan (Dpn, blue), and cortex glia membranes (green) surrounding the neuroblast and encasing the lineage in a glial chamber. Volume‐rendering 3D reconstructions of DL1/2 cluster lineages (red) from control (j) and mutant (k) brains where blue spheres mark the neuroblasts, smaller yellow spheres mark mature INPs of one of the lineages, and green spheres mark those from the other lineage. (l) Quantification of the number of INPs per DL lineage, showing comparisons between the number of INPs in the two lineages (DL1 green and DL2 yellow box plots) from controls and mutants. Comparison of number of INPs of lineages with the highest INPs (green box plots) between control and mutants is shown. Comparison of number of INPs of lineages with the lowest INPs (yellow box plots) between control and mutants is shown. Scale bars represent 10 μm. * *p* < .05, ** p < .01, *** p < .001. (See also Supplementary Figure 11)

To study the cause of this marked reduction, we first used the *earmuff* R09D11 genomic enhancer‐fragment driven reporter CD4‐tdtomato (Han, Jan, & Jan, 2011) to selectively label all type II neuroblast lineages and assess DL1. Type II neuroblast lineages are characterized by the generation of intermediate neural progenitors (INP) that can undergo several rounds of additional asymmetric divisions before they disappear (Boone & Doe, 2008). Within an INP sublineage, which is temporally patterned, gliogenesis is most likely taking place in progeny of the last INP divisions (Bayraktar & Doe, 2013). In control brains, there are eight type II neuroblasts, six of which are positioned medially (DM1‐6) and two laterally (DL1/2), closer to the OL (Figure 6a,b). In mutants, although we observed some brains with instances of DM mispositioning, the DL1/2 cluster was found together and laterally located with respect to the rest of the DM neuroblasts (Figure 6c,d). However, its position with respect to the OL was sometimes changed. To assess proliferation defects in the lineage, our initial approach was to compare control to mutant DL1 clones. However, even though the clonal analysis protocol used in our study was very similar to those employed in other studies analyzing type II clones, which are identified by the presence of INPs (Dpn positive cells in the lineage), we obtained hardly any type II clones (2 out of 116 analyzed clones) and none in the DL1/2 cluster. We thus opted to perform lineage tracing to compare the DL1 glial progeny in control and mutant animals (Figure 6e,f). This analysis showed that in the mutant background there was strong reduction of Repo^+^/DL1^+^ cells (Figure 6f,g). Interestingly, the DL1 neuronal lineage was slightly increased in mutants compared to controls (Figure 6h). In an attempt to understand how these results came about we reasoned that we could use the number of INPs in the lineage as a readout (Figure 6i). Since DL1 and DL2 secondary axon tracts are extremely similar, we differentiated the two lineages through expression of *gcm‐LacZ* in the DL2 lineage (Viktorin et al., 2013) (Supplementary Figure 11A), which consistently contained fewer INPs than DL1 (Supplementary Figure 11B). A comparison between control and mutant revealed that both DL1 and DL2 lineages contained a higher number of INPs in the mutant condition (Figure 6j‐l). This observation suggests that defects in the INP division rate, which could lead to INP accumulation, and/or defects in temporal specification, which could result in the generation of neurons instead of glia, could be responsible for the strong reduction in marginal glia in the OL. In addition, we cannot discard that migration defects could also be one of the contributing factors to the marked reduction in medulla glia in mutant OLs. Since the DL1/2 cluster was found at different relative positions with respect to the OL, and that the IPC, which is the region where these cells enter the OL in normal conditions, is defective in mutants, medulla glia could be hindered from reaching their final destination. However, during the DL1 G‐TRACE lineage analysis in mutant animals we have not detected glial cells in regions other than the optic lobe, which suggests that migrations defects would not be the major contributing factor to the reduced number of medulla glial cells in *ClC‐a* mutant optic lobes.

**Figure 7:**
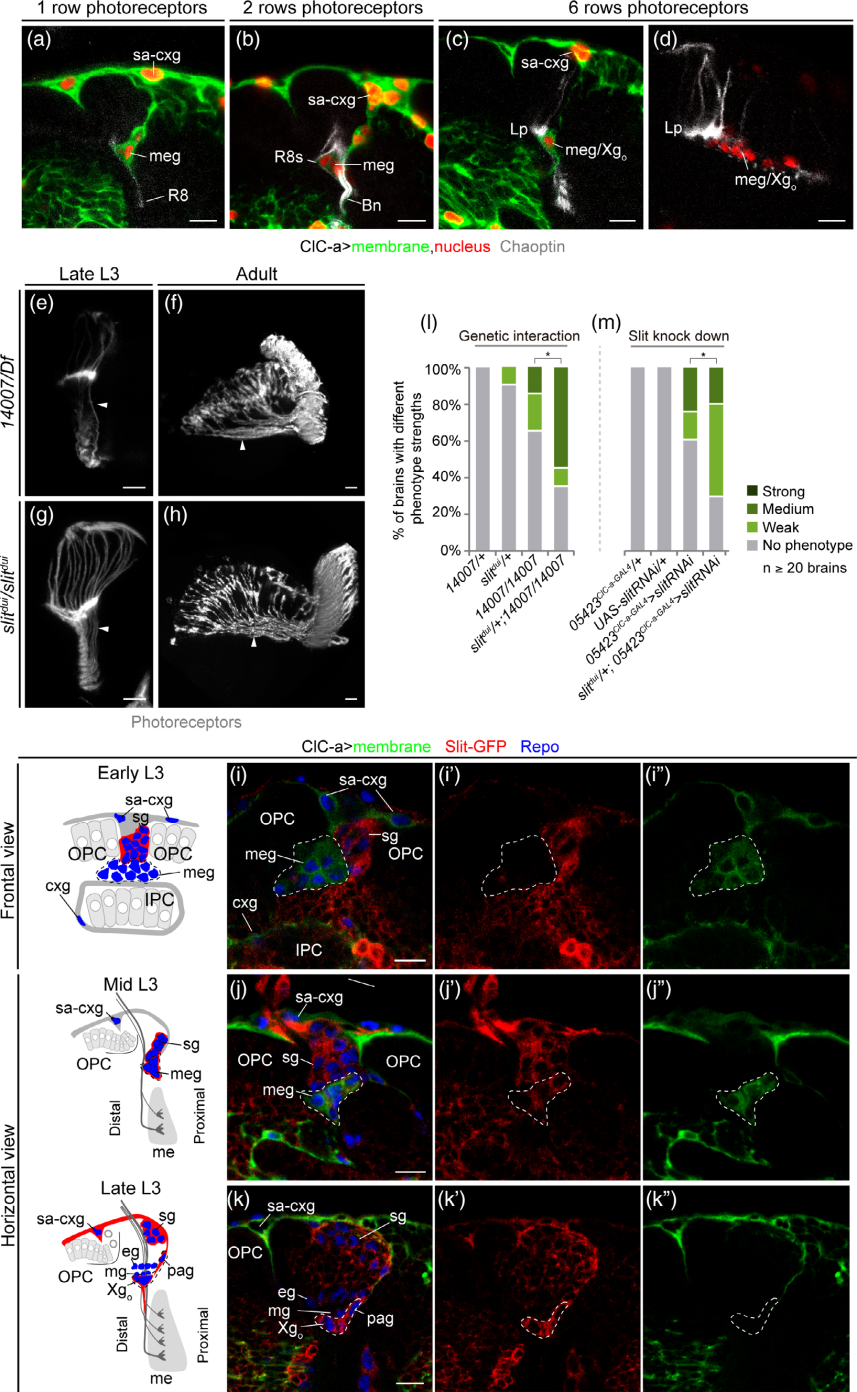
Medulla glia, which express the chemorepellent molecule slit, are in close contact with photoreceptor axons as they innervate the OL. (a–d) Spatiotemporal relationship between photoreceptors and boundary glial cells. Number of photoreceptor rows was inferred from Chaoptin^+^ rows in the eye imaginal disc. Horizontal views of mid L3 optic lobe showing ClC‐a^+^ glia and one (a) and two (b) rows of R8 photoreceptors (Chaoptin, gray). (c) Same view and staining as panels (a) and (b) of a slightly older brain innervated by six rows of photoreceptors. (d) Frontal view showing transversal sections between the line of Xg_o_ cell bodies of photoreceptors on their way to the medulla. Larval (e) and adult (f) examples of photoreceptor (Chaoptin, gray) phenotypes in *ClC‐a* mutants classified as strong. Larval (g) and adult (h) photoreceptor (GMR‐GFP, gray) phenotypes in *slit* ^*dui*^ mutants. Arrowheads show misguided axons innervating the medulla from its proximal face. (i–k) Developmental analysis of Slit expression in glial cells in the barrier. Schematics for the view in each of the stages analyzed are shown. (j) and (k) schematics include photoreceptors for orientation although they are not labeled in the images. Anti‐Repo (blue) was used to label glial nuclei. A Slit‐GFP protein trap (*sli[MI03825‐GFSTF.2]*) that outlines membranes of *slit* expressing cells (Supplementary Figure 12) was used to visualize the *slit* expression pattern (red, i'–k'). ClC‐a^+^ medulla glia (green, i''–k'') are outlined (white dashed line) in (i–i'', j–j''). Xg_o_ and palisade glia are outlined in (k–k''). Although *ClC‐a* expression is downregulated in (k''), we have shown that they express *ClC‐a* in other panels (Figures 1h and 5c,d). (i) Frontal view of an early L3 OL. (j) Horizontal view of a mid L3 OL. (k) Horizontal view of a late L3 OL. Phenotype analysis for *slit/ClC‐a* genetic interaction (m) and *slit* knockdown (l). Phenotype penetrance and expressivity for each condition is depicted as the percentage of brains with no phenotype, weak, medium, and strong phenotypes. Scale bars represent 10 μm. * *p* < .05, (See also Supplementary Figure 12). sa‐cxg, surface‐associated cortex glia; meg, medulla glia; Bn, Bolwig's nerve; Lp, lamina plexus; Xg_o_, outer chiasm glia; sg, satellite glia; OPC, outer proliferation center; IPC, inner proliferation center; eg, epithelial glia; mg, marginal glia; pag, palisade glia; me, medulla

At this point, the question arises of how the marked reduction in medulla glia affects photoreceptor guidance. Since the presence of medulla glia in mid L3 coincides with the beginning of photoreceptor innervation, we next explored the spatiotemporal relationship between these two cell types in control flies. As rows of ommatidia form in the eye disc, photoreceptors extend axons that reach the OL through the optic stalk. In mid L3 stages, R8s from the first rows of ommatidia projected into the posterior part of the LPC field and their axons were located very close to medulla glia as they continued to the medulla (Figure 7a,b). Photoreceptor innervation coincided with cellular rearrangements, when medulla glia started to separate into pag and Xg_o_ glia. Thus, in slightly older brains, R1‐6 axons stopped and formed the lamina plexus above the medulla glia cells that would become Xg_o_, and R8 axons traversed the outer optic chiasm, passing very close to the Xg_o_ (Figure 7c,d) and continued to the medulla, innervating it through its distal face (Figure 5f). Hence, photoreceptors are in close proximity to pag and Xg_o._ Conversely, in mutant brains, the marked reduction in medulla glia, and consequently in Xg_o_, caused posterior R8 axons to skip the outer chiasm and innervate the medulla from its proximal face (Figure 5l). The severity of initial photoreceptor guidance errors determined the strength of the adult guidance phenotypes. Consistent with Xg_o_ and Xg_i_ originating from DL1, in *ClC‐a* mutants chiasms did not properly form, which resulted in altered positioning of OL neuropils in the adult brain (compare Figure 5e with Figure 5k).

Developmental guidance defects and adult outcomes of *ClC‐a* mutants are both extremely similar to OL specific *slit* mutants (Figure 7e–h) and *robo3* mutants (Pappu et al., 2011; Tayler, Robixaux, & Garrity, 2004). The secreted chemorepellent molecule Slit and the Robo family of receptors (Robo, Robo2, Robo3) have been implicated in preventing photoreceptor axons from mixing with distal neuron axons from the lopn during development, hence maintaining compartmentalization of this region of the developing brain (Tayler et al., 2004). While receptors have been shown to be required in neurons, *slit* reporters suggest that Slit protein in the region could be contributed by Xg_o_ (Pappu et al., 2011; Tayler et al., 2004). A detailed developmental analysis of glial barrier assembly allowed us to unequivocally characterize the temporal and cellular expression pattern of *slit* with respect to photoreceptor innervation. To this end, we characterized and used a MiMIC‐based protein trap line for Slit (Supplementary Information, Supplementary Figure 12). Our analysis indicated that Slit was already being expressed in medulla glia in mid L3 (Figure 7i–k), when photoreceptors innervate the brain and their axons come into close proximity with these glial cells. Removal of one copy of *slit* slightly enhanced the *ClC‐a* photoreceptor guidance phenotype when assessed in an allelic combination with few brains showing only medium and weak photoreceptor phenotypes (and presumably with just a slight reduction in medulla glia) (Figure 7l). In addition, knocking down *slit* in ClC‐a^+^ glia in the barrier recapitulated photoreceptor guidance defects (Figure 7m).

Based on our results and previously published studies (Fan et al., 2005; Pappu et al., 2011; Suzuki et al., 2016; Tayler et al., 2004), we propose that the substantial reduction in medulla glia is most probably due to a combination of DL1 lineage proliferation and/or temporal specification defects, which results in a significant reduction in Slit protein in the region. As a consequence, photoreceptors that innervate the OL close to the glial boundary fasciculate with the axons of distal cells (C2, C3, T2 and T3) which derive from the IPC and are known to innervate the medulla from its proximal site (Hofbauer & Campos‐Ortega, 1990; Meinertzhagen & Hansen, 1993).

### 3.6 Expression of *ClC‐a* in cortex glia is sufficient to restore ensheathing glia guidepost cells and rescue photoreceptor guidance defects

**Figure 8:**
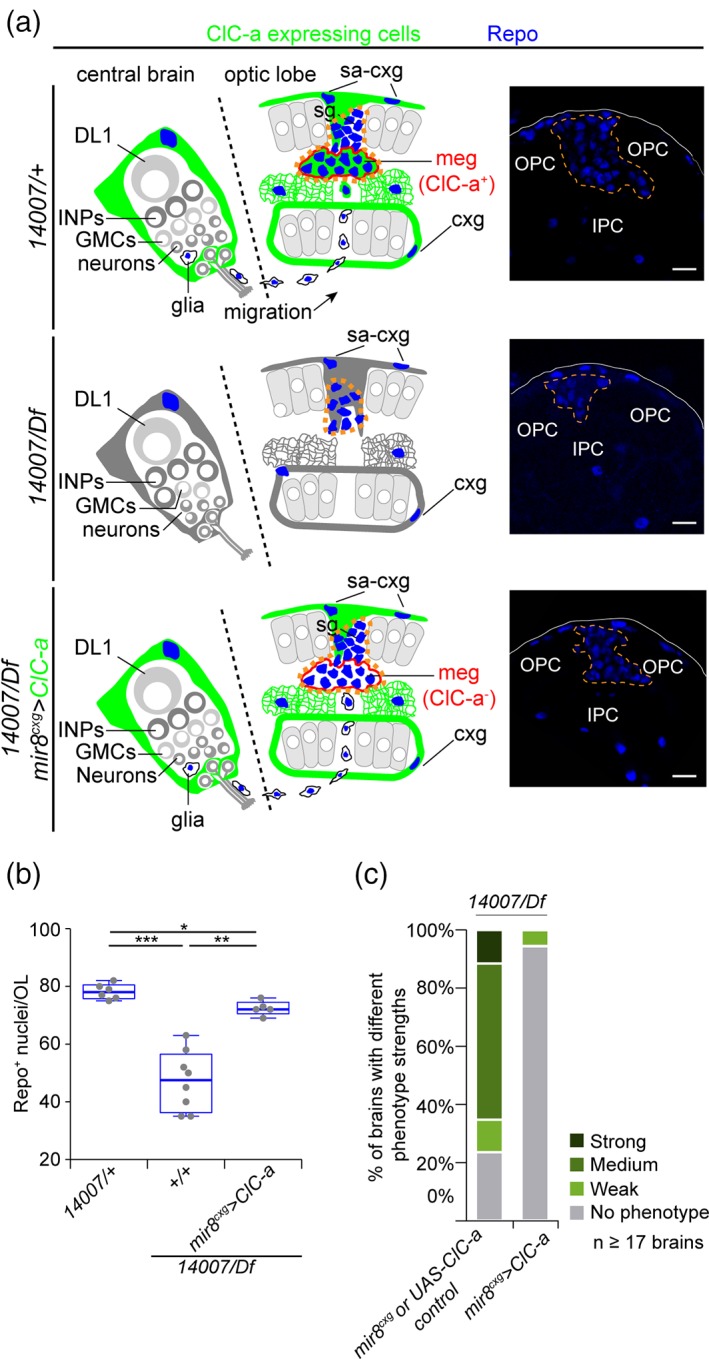
*ClC‐a* expression exclusively in surface‐associated cortex glia and cortex glia rescues the formation of medulla glia and photoreceptor guidance defects. (a) Schematics depicting the cortex glia‐specific rescue experiment and representative confocal sections for each condition. The DL1 lineage and frontal view schematics of mid L3 OLs show *ClC‐a* expression (green) and glial cells (blue). Outlined in a solid red line is the medulla glia population. Outlined in a dashed orange line is the glial population that has been assessed in this experiment. Representative sections of the confocal stacks used for quantification show in a dashed orange line the glial population that was quantified. In controls (*14007/+*) *ClC‐a* is expressed in cortex glia surrounding the DL1 neuroblast and its progeny in the CB, and in surface‐associated cortex glia and cortex glia over the OPC and IPC respectively, and in medulla glial cells in the OL. The *ClC‐a* mutant (*14*,*007/Df*) shows the absence of *ClC‐a* expression and a strong reduction of medulla glial cells; and in an animal where *ClC‐a* expression has been exclusively restored in surface‐associated cortex glia and cortex glia (*mir‐8* ^*cxg*^), medulla glial cells are recovered but do not express *ClC‐a* since they are a subtype of ensheathing glia. (b) Quantification and comparisons of glial nuclei (Repo, blue) in control, mutant, and rescue animals. (c) Quantification of photoreceptor guidance phenotypes in control and rescue brains. Control brains represent genotypes for both the GAL4 driver and the UAS transgene since they could not be distinguished in the genetic scheme of the experiment (*mir‐8* ^*cxg*^ control and *UAS‐ClC‐a* control). Scale bars represent 10 μm. * *p* < .05, ** *p* < .01, *** p < .001. GMC, ganglion mother cell; sa‐cxg, surface‐associated cortex glia; sg, satellite glia; meg, medulla glia; cxg, cortex glia; OPC, outer proliferation center; IPC, inner proliferation center

To test whether *ClC‐a* expression in cortex glia was sufficient to regulate DL1 proliferation, and assess if *ClC‐a* expression in medulla glia (cell type classified as ensheathing glia) played any role in photoreceptor guidance, we performed a cell‐type‐specific rescue experiment. We carried out a surface‐associated cortex glia and cortex glia‐specific rescue. We reasoned that with such a specific driver, we could rescue the generation of medulla glia from DL1 and at the same time avoid *ClC‐a* expression in medulla glia (Figure 8a). Since it was not possible to specifically label medulla glia in this experiment, we used Repo to mark and count glial nuclei in the region in mid L3, when the first photoreceptors begin to innervate the brain. At this time, the glial population is compact and easy to identify, whereas in late L3, additional ClC‐a^−^ glia such as epithelial and marginal glia appear in high numbers and complicate counting. In control animals, mid L3 glia nuclei included ClC‐a^−^ satellite glia and medulla glia (Figure 8a,b). In mutants, the number of glial cells was reduced to half due to the marked reduction in medulla glial cells (Figure 8a,b), but expression of *ClC‐a* exclusively in surface‐associated cortex glia and cortex glia resulted in an almost complete rescue in the number of glial cells present in the barrier region in mid L3 (Figure 8a,b). More importantly, this medulla glia rescue also rescued the photoreceptor guidance phenotype (Figure 8c). Surprisingly, autonomous *ClC‐a* expression in medulla glia was not necessary for their viability, for migration from their point of origin in the CB to position themselves in the OL, or for Slit secretion, since photoreceptor guidance defects were fully rescued when medulla glia were in their position but did not express *ClC‐a*. Thus, we conclude that the strong reduction in medulla glia and the photoreceptor guidance phenotypes are a secondary consequence of the *ClC‐a* requirement in cortex glia and its none autonomous role in neurogenesis.

### 3.7 Mutations in *ClC‐a* result in widespread wiring defects

**Figure 9:**
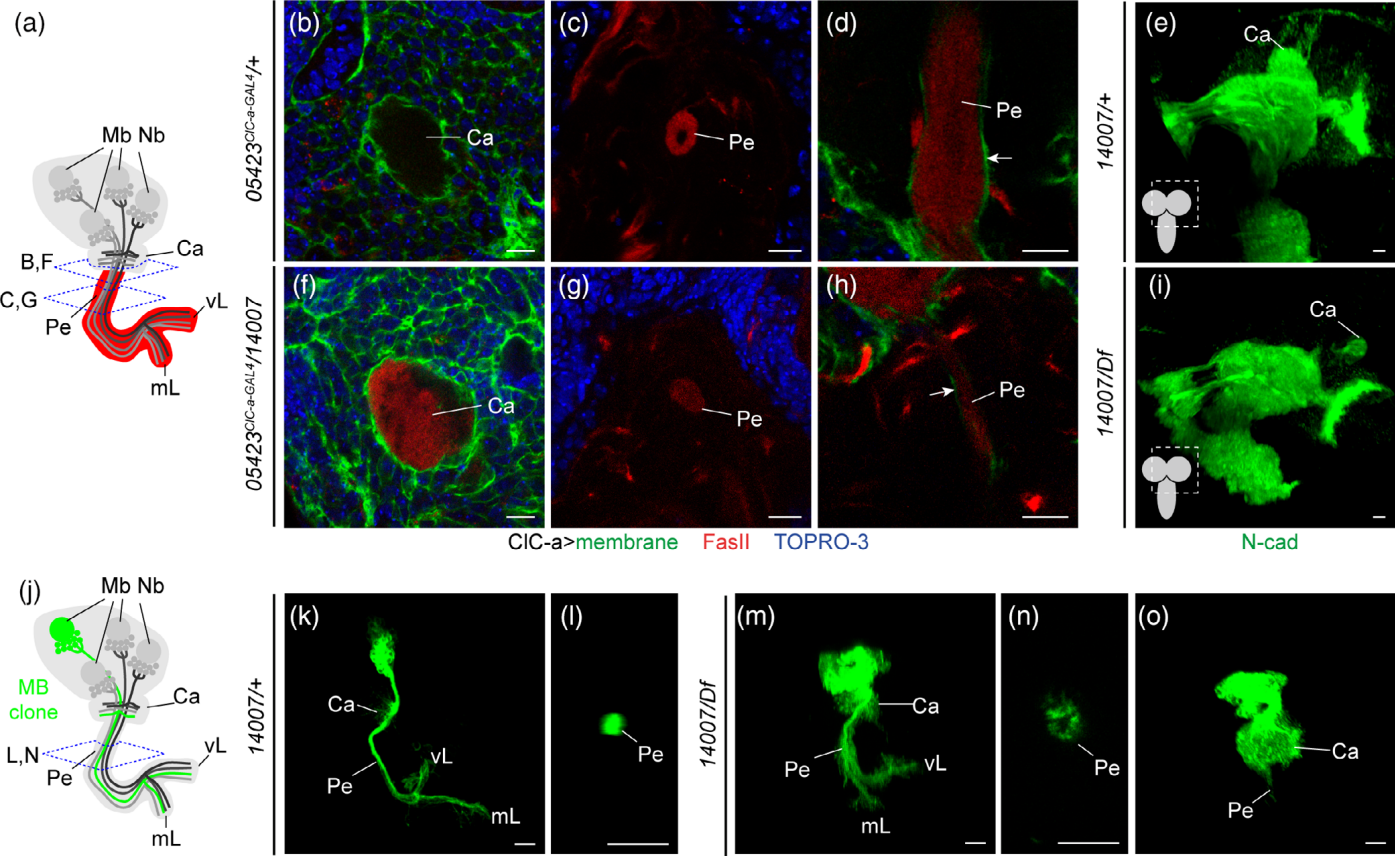
Guidance defects in mushroom body neurons in *ClC‐a* mutants. (a) Schematic of a mushroom body (MB) in one hemisphere. Dashed lines indicate the position of imaging planes and associated letters indicate correspondence to panels. The axonal component of the MB, which consists of the peduncle and lobes, is shown in red, representing anti‐Fasciclin II (FasII) antibody staining. (b–e) Mushroom body analysis in control brains (late L3 *05423* ^*ClC‐a‐GAL4*^ */+* or mid L3 *14*,*007/+*). (b) Confocal section though the calyx region of a control brain showing ClC‐a^+^ glial membranes (green) and all nuclei (blue, TOPRO‐3). (c) Transversal section through the peduncle of a control brain. (d) Longitudinal section of the peduncle showing ClC‐a^+^ tract‐ensheathing glia surrounding it (arrow). (e) Volume‐rendering 3D reconstruction of a control brain hemisphere showing N‐cadherin positive neuropils. (f–i) Mushroom body analysis in *ClC‐a* mutant brains (late L3 *05423* ^*ClC‐a‐GAL4*^ */14007* or mid L3 *14*,*007/Df*) with the same staining as the equivalent control panels. Compare panels (f) to (b), (g) to (c), (h) to (d), and (i) to (e). Schematic (j) and volume‐rendering 3D reconstructions and confocal sections of mushroom body neuroblast clones in control (k, l) and *ClC‐a* mutant (m–o) brains labeled in green. (k) Control clone, (l) cross section of a control clone at the level of the peduncle, (m) mutant clone, (n) cross section at the level of the peduncle of a mutant clone in (m), and (o) Mutant clone with a strong phenotype. Scale bars represent 10 μm. Ca, calyx; Pe, peduncle; mL, medial lobe; vL, vertical lobe

Although we have characterized the origin of the guidance defects seen in photoreceptors, wiring defects are not restricted to this cell type. The position and morphology of neuropils in the visual system of *ClC‐a* animals indicate that the wiring of many other neurons in this system is also probably affected (compare Figure 5e with Figure 5k). Moreover, we also observed defects in CB structures such as mushroom bodies (MBs). Each hemisphere contains one MB, which is formed by the neurons derived from four special type I neuroblasts that never enter quiescence. These neurons extend dendrites forming the calyx, and axons project into a fascicle called the peduncle that splits into two branches called lobes (Figure 9a). Similar to photoreceptors, mushroom bodies are neural structures that are highly dependent on glia–neuron interactions. It has been shown that glia wrap the peduncle and the lobes during development (Spindler, Ortiz, Fung, Takashima, & Hartenstein, 2009) and in the adult (Kremer et al., 2017), and that different type II DM neuroblasts contribute glia that associate with the mushroom body (Ren et al., 2018). In control animals, ClC‐a^+^ glia surrounded the MB calyx (Figure 9b) and the peduncle (Figure 9d). Newly differentiated, FasII^−^ neurons projected their axons through the center of the peduncle, generating a ring‐like FasII^+^ pattern labeling the oldest neurons (Figure 9c). In *ClC‐a* mutant animals, axons often misprojected into the calyx (Figure 9f) and FasII staining filled the center of the peduncle, suggesting that newly generated axons did not project through the center of this structure (Figure 9g). In addition, the peduncle was much thinner (Figure 9g), although it seemed that ClC‐a^+^ glia continued to surround it. Comparison of control and mutant brains stained with antibody against N‐cadherin, which labels neuropils, revealed that the calyx, which in controls appeared deep in the brain (Figure 9e), was more superficial in mutants (Figure 9i). MB clones (Figure 9j) confirmed defects in the calyx and the peduncle (compare Figure 9k‐m with Figure 9o). In MB clones in the control background, axons from the clone stayed together in a bundle and extended into the center of the peduncle (Figure 9l). In instances where MB clones in the mutant background extended axons into the peduncle (Figure 9m), these axons defasciculated and projected into the peduncle through its periphery, leaving older axons in the center (Figure 9n). In clones with strong phenotypes, almost all axons terminated in the calyx and the peduncle was barely visible (Figure 9m). Interestingly, these defects are very similar to those observed when cortex glia and neuropil glia are eliminated: abnormal mushroom body morphologies including splaying of axons and misguidance, and a misshapen superficial calyx due to premature fusion of the four MB lineages in the cortical region (Spindler et al., 2009). Thus, as observed for photoreceptor guidance phenotypes, MB defects in *ClC‐a* mutants may be due to reduced production of glia associated with MB circuitry, whether that glia is ClC‐a^+^ or not. In summary, since guidance defects in the *ClC‐a* mutant seem to be widespread, we propose that the *ClC‐a* requirement for proper circuit assembly is not restricted to the OL but is general to the brain.

## 4 DISCUSSION

In this study, we have shown that the *ClC‐a* chloride channel function in the glial niche has a nonautonomous but profound effect on two key aspects of neural development: the generation of neurons and glia in the appropriate numbers, time, and place, and in consequence, the correct assembly of neural circuits. Importantly, the fact that the fly (ClC‐a) and rat (CLC‐2) chloride channels rescue brain size and guidance defects suggests that both can perform the same physiological function.

The reduced neurogenesis observed in *ClC‐a* mutants could have several origins. Our cell death analysis, clonal study and EdU experiment suggest that in the OL, one of the causes could be defective neuroepithelium expansion. However, another possibility could be that *ClC‐a* function in surface‐associated cortex glia covering the neuroepithelium regulated the proneural wave progression and hence the neurepithelium to neuroblast transition. A premature start of this transition could prevent the completion of neuroepithelial expansion, and hence result in a reduced number of OPC neuroblasts. Alternatively, it is formally possible that the reduced neurogenesis observed is a consequence of both defective neuroepithelium expansion and premature neurepithelium to neuroblast transition. Indeed, glia covering the OPC neuroepithelium has been shown to regulate both processes (Morante et al., 2013; Perez‐Gomez et al., 2013).

Concomitant defects in neuroblast proliferation and photoreceptor targeting have been observed in other studies (González, Romani, Cubas, Modolell, & Campuzano, 1989; Kanai et al., 2018; Zhu et al., 2008), and it has been proposed that the Activin signaling pathway is required to produce the proper number of neurons to enable proper connection of incoming photoreceptor axons to their targets (Zhu et al., 2008). Interestingly, mutations in the proneural gene *asense*, which is expressed in type I neuroblasts, GMCs and INPs, has adult targeting phenotypes that are extremely similar to the ones observed in *ClC‐a* mutants (González et al., 1989). Along the same lines, our study links *ClC‐a* photoreceptor guidance phenotypes to INP defects, and furthermore identifies the INP‐derived glial population required for proper photoreceptor axon guidance. These INP defects could be related to a slower division rate of INPs and/or to an impaired temporal patterning of INPs (Bayraktar & Doe, 2013) affecting the generation of glia. In addition, similarly, defects in neuroblast and/or GMCs divisions or their temporal patterning could explain the reduced size of type I neuroblast clones in *ClC‐a* mutants. Thus, *ClC‐a* role on neurogenesis could be related to the regulation of stem cell/progenitor proliferation and/or precursor differentiation in both the OL and the CB.

In addition to leukoencephalopathy, patients with mutations in *CLCN2* or altered function of the channel also show cognitive impairment. Similarly *CLCN2* mutant mice develop widespread vacuolization that progresses with age, but besides photoreceptor and male germ cell degeneration, they do not display immediately visible behavioral defects (Blanz et al., 2007;Bösl et al., 2001 ; Edwards et al., 2010). However, *CLCN2* is expressed in astrocytes and oligodendrocytes early in development (Makara et al., 2003) and has been detected in Bergman glia (Jeworutzki et al., 2012), which are important for neuronal migration in the formation of cortical structures. Together with our findings, these observations suggest that it would be worth exploring the role of this channel in the vertebrate neural stem cell niche. Interestingly, expression of *CLCN2* has been found outside the brain in an unrelated stem cell niche. It is expressed in Sertoli cells (Bösl et al., 2001), which are the primary somatic cells of the seminiferous epithelium that form the spermatogonial stem cell niche through physical support and expression of paracrine factors (Chen et al., 2005; Oatley, Racicot, & Oatley, 2011). *CLCN2* mutant mice showed disorganized distribution of germ cells in tubules at 3 weeks, germ cells did not pass beyond meiosis I, and were completely lost at later stages (Bösl et al., 2001; Edwards et al., 2010). Hence, similarly to the possible role of ClC‐a regulating neurogenesis in the neural stem cell niche, CLC‐2 could be regulating spermatogenesis in the spermatogonial stem cell niche.

Although the Sertoli *CLCN2* expression/germ cell depletion correlation in mouse is in accordance with our data suggesting an important role of the ClC‐a/CLC‐2 chloride channel in stem cell niches, it remains unclear how a chloride ion channel could nonautonomously modulate neurogenesis. ClC‐a function in Malpighian tubules has been associated with the movement of Cl^−^ ions (Cabrero et al., 2014), but it is possible that its function in glia of the stem cell niche is unrelated to ion exchange. For example, it might recruit signaling molecules to modulate neuroblast proliferation. Conceptually, one way to test whether the channel function is related to the movement of ions would be to perform rescue experiments of *ClC‐a* mutant phenotypes with a channel defective for the pore function. In practice, however, this type of experiment is not that straightforward since CLC‐2 pore gating is quite complex. Channels of the CLC family are thought to function as a homodimers, with each subunit forming a pore and presenting both independent and common pore gating mechanisms (Jentsch & Pusch, 2018). Given the many studies supporting the function of CLC‐2 as a channel, we next discuss different ways in which ionic imbalance caused by mutations in *ClC‐a* could result in the phenotypes described. One of the possibilities we considered was whether ionic imbalance in *ClC‐a* mutants affected secretion. Glial cells secrete different types of factor to the extracellular space, both during development and to maintain morphology in the adult (Coutinho‐Budd et al., 2017; Read, 2018; Spéder & Brand, 2018). In the niche in particular, there are several examples of glia‐secreted molecules that regulate neurogenic proliferation, such as the transforming growth factor a (TGF‐a)‐like ligand (Morante et al., 2013) and insulin‐like peptides (dILPs) (Chell & Brand, 2010; Sousa‐Nunes et al., 2011). In vertebrates, an increase in intracellular Ca^2+^ in astrocytes, which is caused by activation of G protein–coupled receptors and release of calcium from intracellular stores or calcium entry from the extracellular space through different types of channel, has been reported to evoke the release of gliotransmitters (Bazargani & Attwell, 2016; Khakh & McCarthy, 2015; Shigetomi, Patel, & Khakh, 2016). In this scenario, membrane potential changes mediated by Cl^−^ channel activity could modulate activation of GPCR or voltage dependent Ca^2+^ channels, mediating an increase in the Ca^2+^ intracellular concentration and resulting in secretion. In fact, the opening of voltage dependent Ca^2+^ channels has been proposed as the mechanism behind the increase in aldosterone production and secretion (Fernandes‐Rosa et al., 2018) resulting from gain‐of‐function mutations of *CLCN2*, which are behind primary aldosteronism and cause sustained depolarization of glomerulosa adrenal cells (Fernandes‐Rosa et al., 2018; Scholl et al., 2018). To test whether loss of function of ClC‐a/CLC‐2 channels also affected secretion, we performed glia‐specific RNAi knock down of key upstream regulators of intracellular calcium release such as *Drosophila* IP3R and RyR receptors, and downstream effectors of calcium‐regulated secretory vesicle exocytosis, as well as secretion assays in primary glial cultures where *CLCN2* was knocked down with RNAi (data not shown). However, we were unable to consistently recapitulate *ClC‐a* mutant phenotypes or detect secretion defects, suggesting that if the absence or reduction of the channel impairs secretion, it does so only in a very limited way.

Another possibility is that ClC‐a is involved in pH regulation. Under extracellular neutral pH, H^+^ and HCO_3_^−^ combine to form H_2_CO_3_, which in turn is in equilibrium with H_2_O and CO_2_. In acidic conditions, to compensate for the increase in H^+^, the HCO_3_^−^/Cl^−^ exchangers extrude HCO_3_^−^ to the extracellular space to form more H_2_CO_3_ and drive the reaction to the formation of H_2_O and CO_2_. Rat ClC‐2 opens in response to extracellular acidification, allowing Cl^−^ to exit the cell (Jordt & Jentsch, 1997). Since for each molecule of HCO_3_^−^ extruded, one of Cl^−^ is internalized, ClC‐2 activation might be required to regulate HCO_3_^−^ transport and allow the presence of extracellular Cl^−^, thus creating a Cl^−^ recycling pathway for HCO_3_^−^/Cl^−^ exchangers (Bösl et al., 2001). Assays in *Xenopus* oocytes have shown that ClC‐a activity is also sensitive to pH (H. G‐P. and R. E., unpublished results). Thus, it may be that the lack of ClC‐a in cortex glia leads to a more acidic extracellular pH due to deficient Cl^−^ recycling for HCO_3_^−^/Cl^−^ exchangers. Since changes in extracellular and intracellular pH have been shown to affect the proliferative capacity of both wild type and cancer cells (Carswell & Papoutsakis, 2000; Ciapa & Philippe, 2013; Flinck, Kramer, & Pedersen, 2018; Persi et al., 2018; White, Grillo‐Hill, & Barber, 2017), ClC‐a function in pH regulation could explain the proliferation defects observed in the mutant.

Regardless of the molecular mechanism that mediates the effect of ClC‐a on neurogenesis, our findings support the notion that glia‐mediated ionic balance could be important for brain development. Our results are in accordance with those of recent studies suggesting a link between several ion channels and the development of the nervous system, with channels being important both in stem cells (Li, 2011; Liebau, Kleger, Levin, & Yu, 2013) and glia (Olsen et al., 2015). A recent example of a channel function in stem cells is the gene SCN3A, which codes for the NaV1.3 sodium channel. This channel is mainly expressed during development and is highly enriched in basal/outer radial glia progenitors and migrating newborn neurons (Smith et al., 2018). The appearance of this type of progenitor and defined neuronal migration has been associated with the establishment of gyri in the cortex (Fietz et al., 2010; Hansen, Lui, Parker, & Kriegstein, 2010; Reillo, De Juan Romero, García‐Cabezas, & Borrell, 2011). Intriguingly, mutations in the SCN3A gene result in structural malformations of gyri in the cortex (Smith et al., 2018). Another example is the glial‐specific Kir4.1 channel, which is related to neurodevelopmental disorders with associated cognitive defects. Mutations in *KCNJ10*, which codes for the glial‐specific Kir4.1 channel, underlie SeSAME/EAST syndrome (seizures, sensorineural deafness, ataxia, intellectual disability and electrolyte imbalance/epilepsy, ataxia, sensorineural deafness, and tubulopathy) (Bockenhauer et al., 2009; Scholl et al., 2009) and have been detected in patients diagnosed with autism spectrum disorder and epilepsy (Sicca et al., 2011, 2016). Reduced Kir4.1 expression in astrocytes significantly contributes to the etiology of Rett syndrome (Kahanovitch et al., 2018; Lioy et al., 2011), which shares many pathophysiological traits with SeSAME/EAST. Moreover, Kir4.1 protein is detected as early as embryonic day 20 in glial cells in the developing cortex and hippocampus (Moroni, Inverardi, Regondi, Pennacchio, & Frassoni, 2015), suggesting that it could influence neural development in these regions. Together with our findings, these observations suggest that mutations in ion channels could affect neurogenesis and connectivity, resulting in intellectual disabilities. Thus, providing insights into the developmental stages affected by impaired glial‐dependent homeostasis could help improve our understanding of the origin of the cognitive deficiencies detected in patients with channelopathies or conditions where ion channels in glia are not functional.

## AUTHOR CONTRIBUTIONS

M.M. and R.E. conceived the project; M.M., H.P‐S., Q.Z., R.E., and H. G‐P. designed the experiments and data analysis; H. G‐P. and R.E. contributed reagents and analytical tools; M. R. designed the statistical analysis; H.P‐S. and Q.Z. performed the experiments; H.P‐S., Q.Z, M.R., and M.M. analyzed the results; and M.M. wrote the manuscript with contributions from all the other authors.

## CONFLICT OF INTEREST

The authors declare no potential conflict of interest.

## Supporting information


**Appendix S1**: Supplementary Information.

**Supplementary Figure 1. Comparative analysis of *ClC‐a* expression patterns with antibody and various reporters.**
(A‐C) Detection of *ClC‐a* expression (green) in stellate cells of adult Malpighian tubules using anti‐ClC‐a antibody (A), the *ClC‐a‐GFP* protein trap (B), and the *ClC‐a‐GAL4* driver line combined with a membrane reporter (green) (C). Nuclei labeled with TOPRO‐3 where indicated.(D‐I) Detection of *ClC‐a* expression in late L3 brain hemispheres. (D‐F) Horizontal views of the surface of brain hemispheres. Antibody staining (D), protein trap (E), and driver (F) show the same expression patterns. Asterisks mark some neuroblast chambers. (G‐I)Horizontal views deeper in hemispheres, in the optic lobe area. Arrowheads point to *ClC‐a* expression between the LPC and lopn. (G) Antibody staining shows expression on the OPC, in the LF, and in between the LPC and lopn. (H) In addition, the protein trap construct also reveals expression deeper in the brain, around the IPC and forming a mesh‐like structure inside the hemisphere, where the antibody did not penetrate. Inset shows expression between the LPC and lopn. Anti‐E‐cad staining (magenta) was used to identify the neuroepithelial cells and anti‐Chaoptin (gray) label photoreceptors. (I) The *ClC‐a‐GAL4* driver mediated membrane labeling (green) pattern is very similar to the one observed with the antibody and the protein trap construct, including the signal detected between the LPC and lopn. Glial nuclei werel abeled with anti‐Repo antibody (blue). Not all glial nuclei are ClC‐a^+^(red). (J‐K) *ClC‐a‐GFP* protein trap expression surrounding type I (J‐J’’) and type II (K‐K’’) neuroblasts labeled with anti‐Dpn antibody (blue). (J‐J’’) Confocal sections at different levels of a type I neuroblast (arrow) show the presence of ClC‐a‐GFP protein surroundingit. (K‐K’’) Confocal sections at different levels of a type II neuroblast (arrow) show the presence of INPs (asterisks) also labeled with anti‐Dpn.ClC‐a‐GFP is seen surrounding the neuroblast and delineating the chamber encasing the INPs and the neuroblast lineage.
CB, central brain; OL, optic lobe; LF, lamina furrow; LPC, lamina precursor cells; lopn, lobula plate neurons; OPC, outer proliferation center; IPC, inner proliferation center. Scale bars represent 10 µm.

**Supplementary Figure 2. Identification of *ClC‐a* expressing glia.**
Confocal sections showing *ClC‐a* expression pattern in the late L3 nervous system (A‐D), and the optic lobe in pupal stages (E, F) and adult (G). *ClC‐a* specific GAL4 driver was used to label cellular membranes (green) and nuclei (red) of ClC‐a^+^ cells. Glial nuclei were labeled with anti‐Repo antibody (blue) and photoreceptor cells with anti‐Chaoptin (gray). (A) Larval brain where, besides a ClC‐a^+^ signal in cortex glia both in brain hemispheres and the VNC, a ClC‐a^+^ signal is detected in neuropil‐ensheathing glia in the VNC, tract‐ensheathing glia in connectives between the two hemispheres, and in peripheral nerves. (B,C) Cross section (B) and longitudinal section (C) of peripheral nerves containing ClC‐a^+^ glia. Dashed line outlines the nerve. (D) Image of the optic stalk, which connects the eye disc and the optic lobe. ClC‐a^+^ glia wraps this bundle formed by photoreceptor axons on their way to the optic lobe. Photoreceptor cell bodies are seen in the eye disc in gray and their axons in the optic lobe. Photoreceptors do not express *ClC‐a*. (E, F) Based on the ClC‐a^+^ Repo^+ ^nucleus position, we can identify the following as *ClC‐a* expressing glia: cxg, wg/dsg, Xg_o_, Xg_i_,mneg, and lopneg in 20 (E) and 50 (F) hrs After Pupal Formation (APF). (G) *ClC‐a* expression is maintained in the adult. Signal in the medulla and lobula neuropils belongs to mneg and lopneg described projections into these structures.
egt, tract‐ensheathing glia; egn, neuropil‐ensheathing glia; wg, wrapping glia; pn, peripheral nerve; ed, eye disc; os, optic stalk; OPC, outer proliferation center; LPC, lamina precursor cells; BBB, blood brain barrier; cxg, cortex glia; megn, medulla neuropil‐ensheathing glia; ep, epithelial glia; mg, marginal glia; Xg_o_, outer chiasm glia; Xg_i_, inner chiasm glia; psg, proximal satellite glia; wg/dsg, wrapping glia/distal satellite glia; lopegn, lobula plate neuropil‐ensheathing glia. Scale bars represent 10 µm.

**Supplementary Figure 3. Immunohistochemistry and western blot analysis of *ClC‐a* MiMIC alleles.**
(A, B) Anti‐ClC‐a antibody staining of adult Malpighian tubules in control animals (A) and *14007/Df* mutants (B). (C, D) Anti‐ClC‐a antibody staining of late L3 brains in control animals (C) and *14007/Df* mutants (D). Photoreceptors are labeled with anti‐Chaoptin (green).(E) Western blot of protein extraction from HEK293 cells transfected with or without *ClC‐a isoform C 3xFlag* pcDNA3.1. Both anti‐Flag and anti‐ClC‐a antibodies detect a band below 130 kDa, which is possibly the weight of the protein (Uniprot prediction at 118 kDa) plus glycosylation. (F) Western blot of protein extraction from adult heads of controls and different allelic combinations. The signal around the 130 kDa mark reflects the presence of ClC‐a protein in controls, most probably of different isoforms which range from 113 to 132 predicted kDa plus glycosylation. A strong reduction in this signal is observed in mutant animals.
Scale bars represent 10 μm.

**Supplementary Figure 4. Analysis of eye development in *ClC‐a* mutants and *ClC‐a* requirement in the eye.**
(A‐C) Images of adult eyes of controls (A, B) and a *ClC‐a* mutant allelic combination (C). In all cases ommatidia are stereotypically arranged. (D‐I) Confocal images of developing ommatidia in control (D, F, H) and mutant (E, G, I) eye discs. The R7 is marked by anti‐Prospero antibody; the anti‐Boss antibody labels this R8 specific receptor, and the anti‐Cut antibody labels the cone cells. No differences between the control and mutant expression patterns are observed. Together with the wild type external eye morphology this data show that eye development is normal in *ClC‐a* mutants. (J‐M) Analysis of *ClC‐a* expression (red) at different stages of eye development. In the eye disc (J) and 40 hrs APF retina (K, L) photoreceptors are labeled with anti‐Chaoptin (green). In the adult retina (M), photoreceptor rhabdomeres are labeled with Phalloidin. Nuclei are marked with TOPRO‐3 where specified. Anti‐ClC‐a antibody does not label the eye tissue at any of the stages analyzed. (N‐R) Assessment of *ClC‐a* requirement in the eye. (N‐P) Representative confocal sections of photoreceptor arrays (green) of control (N) and *ClC‐a* mutant (O) eyes generated by the EGUF/hid technique and quantification of brains with phenotype (P). (Q, R) Quantification of the percentage of brains with different strengths of guidance phenotypes in photoreceptor‐specific knock down (Q) and rescue experiments (R). Consistent with the absence of *ClC‐a* expressionin photoreceptors, misguidance phenotypes are non‐autonomous. Hence, eye specific *ClC‐a* knockout and knockdown results in proper photoreceptor guidance and eye specific *ClC‐a* expression in mutants does not rescue photoreceptor guidance phenotypes.
Scale bars represent 10 μm.

**Supplementary Figure 5. Analysis of non‐autonomous layer selection defects in misguided photoreceptors of *ClC‐a* mutants.**
Confocal images of adult (A‐G) and pupal (H,I) optic lobes stained with anti‐Chaoptin to label all photoreceptors (green). Photoreceptor subtypes were labeled using cell type specific opsin reporters: R1‐6 (magenta), R7 (blue), and R8 (red). An R8 specific driver (*senseless)* was used to label R8s in pupal brains (red). (A) Control photoreceptor array showing R1‐6 photoreceptors stopping in the lamina. (B, C) Mutant arrays. (B) In mutant animals with weak guidance defects, R1‐6 terminate normally in the lamina. (C) In animals with strong guidance defects, R1‐R6 axons invade the medulla as seen in the inset (C’). (D) Control array showing R7s terminating at the M6 layer. (E) Mutant array shows misguided R7s terminating in the M6 layer like controls. (F) Control array showing R8s terminating in the M3 layer. (G) Mutant array showing misguided R8s terminating in the M1 layer. (H) Control array at 40 hrs APF. R8 cells terminate in the prospective M1 layer at the top of the medulla. This is a temporary stop since in a second stage they actively extend to the M3 layer. (I) Misguided R8s in the mutant animal also terminate in the M1 layer; however, the adult phenotype suggests that these cells are unable to detach from this temporary layer and retract to the M3. (J) Quantification of adult targeting defects in misguided photoreceptors. Most R8s terminate in M1 (red) instead of at M3, while most R7s terminate correctly at M6. The limited number of R7 targeting defects can be explained by the fact that in pupal stages, R7s already extend to a deeper layer with their growth cones very close to their synaptic partners, and that the R7 axons grow by intercalation of ingrowing processes of other neurons. n=number of photoreceptors analyzed.
Scale bars represent 10 µm.

**Supplementary Figure 6. Quantification of ClC‐a^+^ cortex glia nuclei and central brain neuroblasts in control and *ClC‐a* mutant brain hemispheres. **
(A) Ratio of cortex glia nuclei/µm^3^ in late L3 control (*05423^ClC‐a‐GAL4^/+*) and mutant (*05423^ClC‐a‐GAL4^/14007*) brain hemispheres. (B) Quantification of the number of CB neuroblasts present in late L3 control (*14007/+*) and mutant (*14007/Df*) brain hemispheres. 1
n.s.>0.05, *p<0.05.

**Supplementary Figure 7. Study of neuroepithelium to neuroblast transition in *ClC‐a* mutant animals.**
Lateral views of volume‐rendering 3D reconstructions of late L3 larval hemispheres. (A) Control animal (*14007/+*) stained with anti‐E‐cad (gray, A’) labeling the OPC and anti‐Dpn (red, A”) labeling neuroblasts, which are differentiating on the medial side of the OPC. Double arrow marks the width of the OPC in the central region. (B, C) Examples of OPC defects observed in late L3 mutant hemispheres (*14007/Df*). (B) E‐cad staining (B’) reveals a reduction in the width of the OPC (double arrow), especially in the central part. (C) In this severe example, although E‐cad staining is gone (bracket), there are neuroblasts, suggesting that the neuroepithelial to neuroblast transition in this region of the OPC took place prematurely and there is no more OPC tissue. (D) Control animal (*14007/+*) stained with anti‐L'sc (green), which labels the neuroepithelial cell that will transition to neuroblast, and anti‐Dpn (red) to visualize neuroblasts. (E) Mutant animal that lacked *L'sc* expression in the central region of the OPC. The absence of L'sc indicates that there was no more neuroepithelium to differentiate into neuroblasts. The presence of neuroblasts (red) in the region where L'sc is missing indicates that there used to be neuroepithelium. It is worth noting that, in addition to premature neuroepithelium to neuroblast transition, neurons generated by the d‐IPC could also contribute to this phenotype by occupying this space. Invasion of these neurons could be a consequence of the disruptionof the glial barrier, a phenotype described in Figure 5.
OPC, outer proliferation center; LPC, lamina precursos cells; lopn, lobula plate neurons. Scale bars represent 10 µm.

**Supplementary Figure 8. Comparison of EdU labeling in control and *ClC‐a* mutant OPC neuroepitheliums.**
(A‐B) Normalized quantification of OPC neuroepithelial EdU‐labeled cells in control and mutant animals. The S‐phase starts as a wave from one side of the nucleus, and hence, depending on S‐phase progression during the incubation time, EdU labeling appears as a crescent for cells in early S‐phase or it fills the whole nucleus in cells that reach the late S‐phase.(A) Ratio of EdU^+^ OPC cells/total OPC cells. No differences were observed between control and mutant animals. (B) Ratio of late S‐phase EdU^+^ OPC cells /total EdU^+^ OPC cells. This ratio was lower in mutants than in controls. (C, D) Representative confocal sections of EdU‐labelled neuroepithelial OPC cells in control (C) and mutant (D) animals. E‐cad signal (green) labels neuroepithelial cells and TOPRO‐3 (blue) nuclei. Asterisks mark cells that have incorporated EdU (red) in the whole nucleus while arrowheads mark cells were EdU signal is restricted to part of the nucleus. These images and the quantification show that during the incubation time frame (15 minutes), less cells achieve complete EdU incorporation in mutants compared to controls, which would be in accordance with impaired proliferation and smaller clones.
Scale bars represent 10 µm. n.s.>0.05, * p<0.05.

**Supplementary Figure 9. Characterization of a surface‐associated cortex glia and cortex glia‐specific driver.**
Expression patterns of *mir‐8‐GAL4* driver and *mir‐8 ^cxg^*. Membranes were labeled in green, nuclei in red, and all glial nuclei in blue (anti‐Repo). (A,B) Horizontal views at the surface of the central brain showing *mir‐8* (A) and *mir‐8 ^cxg^* expression (B). (A’, B’) Magnifications of dashed region of interest in (A) and (B). (A’) *mir‐8‐GAL4* is expressed in neuroblasts (arrowhead) and neurons (arrow). The gain of the red channel has been increased to visualize nuclear signal in neuroblasts and neurons. (B’) Magnification of dashed region of interest in (B). Using the same gain as in (A’), neuronal and neuroblast labeling is gone using the *mir‐8 ^cxg^* transgenes. (C, D) Horizontal views deep in the brain hemisphere showing *mir‐8‐GAL4* (C) and *mir‐8 ^cxg^* expression (D). (C) Neuronal *mir‐8* expression is seen in the mushroombody calyx. Xg_o_ glia do not express *mir‐8*. (D) No neuronal expression was detected in the calyx or Xg_o_.(E) Frontal view of a volume‐rendering 3D reconstruction of a mid L3 optic lobe. No membrane (green) and/or nuclear (red) signal between the OPC and IPC confirmed that *mir‐8 ^cxg^* was not expressed in boundary glia.
CB, central brain; OL, optic lobe; cxg, cortex glia; LF, lamina furrow; OPC, outer proliferation center; IPC, inner proliferation center; Xg_o_, outer chiasm glia; Ca, calyx. Scale bars represent 10 µm.

**Supplementary Figure 10. Developmental details of the formation of the glial barrier between the LPC and the lopn.**
(A‐C) Characterization of cell types in the barrier. Specific drivers were used to label membranes in green or red. Glial nuclei are labeled with anti‐Repo (blue). (A‐B) Horizontal views of early (A) and late (B) optic lobes showing ClC‐a^−^ satellite glia population membranes labeled with the *R43H01‐LexA* specific driver in red and ClC‐a^+^ membranes (*05423* ^*ClC‐a‐GAL4*^ */+*) in green. (C) Horizontal view of a late L3 optic lobe showing Xg_o_ and palisade glia membranes labeled with the specific driver *R25A01‐GAL4* in green. This driver is not expressed at earlier developmental time points, and thus cannot be used to manipulate these cell types when they group together as boundary glia before photoreceptor innervation in mid L3. (D‐F) DL1 lineage tracing to analyze parallelisms between the timing of visualization of DL1 derived Xg_o_ glia and visualization of ClC‐a^+^ boundary glia (prospective Xg_o_ and pag) in the optic lobe. DL1 lineage (green) is visualized with the DL1 specific driver *R38H02‐GAL4*, which is expressed in this NB early in development in a short time window, and the G‐TRACE system. Optic lobes were stained with anti‐E‐cad (magenta) to identify neuroepithelial cells and anti‐Repo (blue) to identify glial cells. (D) Frontal view of volume‐rendering 3D reconstructions of a wild type early L3 optic lobe showing DL1 progeny (green) in the same region as ClC‐a^+^ cells in Figure 5a. Neuroepithelia were segmented and the rest of the signal masked to avoid background noise and allow better visualization. (E) Horizontal view of a confocal plane showing the neural progeny of the DL1 lineage in the central brain (Repo^−^) and the Xg_o_ and Xg_i_ glial progeny in the optic lobe (Repo^+^). (F) Frontal view of a volume‐rendering 3D reconstruction of a wild type mid/late L3 brain showing DL1 progeny (green) in the same region as *ClC‐a* ^+^ cells in Figure 5b. (G, H) *ClC‐a* lineage tracing, performed with the *05423* ^*ClC‐a‐GAL4*^ driver and the G‐TRACE system, to analyze the drop in ClC‐a^+^ cells from mid to late L3. Anti‐Repo was used to label glial cells. (G) Frontal view of a volume‐rendering 3D reconstruction of a late L3 brain. (G’) G‐TRACE green labeling indicates that cells expressed *ClC‐a* at some point during development. (G”) G‐TRACE red labeling shows cells currently expressing *ClC‐a*. Bracket and arrowhead respectively demarcate Xg_o_ and pag that had already expressed *ClC‐a* in early and mid L3 (K’), but downregulated *ClC‐a* expression in late L3 (K″). (H) Green box plots show the number of nuclei per brain that expressed *ClC‐a* at a developmental time prior to the larval stage analyzed. Red box plots show the number of nuclei per brain that currently express *ClC‐a*. Dots in box plots represent data points. Comparisons between green and red box plots are shown for each developmental time analyzed. Both in mid and late L3, there are more cells that used to express *ClC‐a* than cells that currently express it, indicating that some downregulation of *ClC‐a* expression is occurring at mid and late L3 stages. The number of nuclei currently expressing *ClC‐a* in mid L3 quantified with *05423* ^*ClC‐a‐GAL4*^ mediated G‐TRACE (*UAS‐nsl‐DsRed*) is lower than the number of cells observed when using *05423* ^*ClC‐a‐GAL4*^ and *UAS‐H2B‐RFP*, presumably due to the use of different UAS reporters. Compare mid L3 current G‐TRACE value (red box plot) in (H) to the value in *05423* ^*ClC‐a‐GAL4*^ */+* animals at mid L3 in Figure 5M. (I, J) Assessment of cell death in mutants in the region where boundary glia would normally be positioned. Early L3 (I) and mid L3 (J) mutant brains showing, as expected, few (I) or no (J) ClC‐a^+^ boundary glia nuclei (red). Most of the sporadic Dcp‐1 signal observed (arrowheads, gray) is in non‐glial cells (Repo^−^).
OPC, outer proliferation center; IPC, inner proliferation center; sg, satellite glia; bg, boundary glia; pag, palisade glia; Xg_o_, outer chiasm glia; Xg_i_, inner chiasm glia; cxg, cortex glia. Scale bars represent 10 μm. n.s. > .05, * p < .05, ** p < .01.

**Supplementary Figure 11. DL1/DL2 distinction based on *gcm* expression.**
(A‐A”) Confocal sections showing the progeny of DL1 and DL2 neuroblasts labeled by R9D11‐tdtom expression (red). *gcm‐lacZ* expression (green, A’) labels part of the DL2 lineage (arrow). INPs (arrowheads) are labeled in anti‐Deadpan antibody (gray, A”). (B) Quantification and comparison of the number of INPs per lineage. Scale bars represent 10 μm. ***p < .001

**Supplementary Figure 12. Comparison of *slit‐*LacZ (*sli*** ^***05428***^ **) and Slit‐GFP (*sli[MI03825‐GFSTF.2]*) expression patterns.**
*sli* ^*05428*^ is a commonly used nuclear lacZ reporter of *slit* expression. We characterized Slit‐GFP expression pattern because *sli* ^*05428*^ nuclear LacZ expression at early and mid L3 stages was very low and difficult to distinguish from background. Slit full‐length protein can be cleaved into large N‐terminal (Slit‐N) and short C‐terminal (Slit‐C) fragments. Slit‐FL and Slit‐N are more tightly associated with the cell surface, whereas Slit‐C is mostly shed into the extracellular space (Brose et al., 1999). The GFP tag in this Slit‐GFP reporter line was located between amino acids 398‐399, in the second LRR repeat, so in the Slit‐N terminal fragment. Thus, the GFP signal of the Slit‐GFP reporter stays in the membrane of the *slit* expressing cells. *Slit* signal for both *slit*‐lacZ and Slit‐GFP reporters is shown in red. *ClC‐a* ^+^ membranes are labeled with *05423* ^*ClC‐a‐GAL4*^/*UAS‐mCD8‐mRFP* and shown in green. Glia nuclei are labeled with anti‐Repo antibody (blue). (A, B) Horizontal views through the VNC showing nuclear LacZ signal (red, A, A’) and membrane Slit‐GFP signal (red, B, B′) in midline glia. (C, D) Frontal views of late L3 optic lobes. (C) Nuclear LacZ signal (red) can be seen in Xg_o_ and medulla neurons as previously reported (Suzuki et al., 2016; Tayler et al., 2004), as well as in cortex glia. In early pupal stages, LacZ expression in Xg_o_ is stronger than in late L3 (data not shown). (D) Membrane Slit‐GFP signal (red) is seen in the same cell types as *slit*‐LacZ: Xg_o_, cortex glia, and medulla neurons. Thus, the Slit‐GFP expression pattern is the same as the one observed with *slit*‐lacZ.
midg, midline glia; sa‐cxg, surface‐associated cortex glia; pag, palisade glia; Xg_o_, outer chiasm glia; mn, medulla neuron. Scale bars represent 10 μm
