# Supplementary material for: Drosophila ClC‐a is required in glia of the stem cell niche for proper neurogenesis and wiring of neural circuits
Source: Glia. 2019 Sep 3;67(12):2374–98. doi: 10.1002/glia.23691 (PMC6851788; doi:10.1002/glia.23691)
Supplement: Supplementary file 1 — Appendix S1: Supplementary Information. [file GLIA-67-2374-s002.docx]

**SUPPLEMENTARY INFORMATION**

This file contains

-Supplementary Material and Methods

-Supplementary Figure Legends

-References

-Genotype list

**Supplementary Material and Methods**

**Genetics**

ClC-a-GFP: As with *ClC-a-GAL4*, the ClC-a-GFP line we used was also derived from *Mi(MIC)ClC-a^05423^* by the Gene Disruption Project. The protein trap cassette used to generate ClC-a-GFP contains the GFSTF tag (EGFP-FlAsH-StrepII-TEV-3xFlag) flanked by flexible linkers on both sides in the appropriate phase. The ClC-a tagged protein generated includes the GFP sequence in frame in an extracellular loop of the protein. Homozygous animals are viable and wild type, indicating that the ClC-a-GFP protein is functional.

*ClC-a* mutant developmental delay: To be able to appropriately compare controls and mutants during development we wanted to confirm that the 24-hour delay observed in eclosion time between controls and the *14007/Df* and *05423^ClC-a-GAL4^/14007* allelic combinations was also developmental. To this end we compared the timeline of developmental landmarks such as photoreceptor innervation and pupariation between control and mutants. The third instar larval stage (L3) can be divided into early, mid, and late stages. In wild type and heterozygous control animals, photoreceptor innervation takes place at mid L3 (96 hours After Egg Lay (AEL)) and pupariation at late L3 (120 hrs AEL). However, in *14007/Df* and *05423^ClC-a-GAL4^/14007* mutant animals, photoreceptors did not enter the brain until 120 hrs AEL and pupariation did not take place until 144 hrs AEL. Thus, confirming the 24-hour delay in development between control and mutant. Glia-specific rescue experiments also rescued the developmental delay. To compare control and mutant animals at the same developmental stage, we took developmental delay into account, and for the sake of simplicity, we refer to comparisons in larval stages as opposed to developmental hours (i.e., we refer to comparisons as control versus mutant at mid L3 rather than 96 hrs AEL control versus 120 hrs AEL mutant). In all cases, adult mutant animals were of the same size as heterozygote controls and wild type flies (data not shown).

Slit-GPF: similar to ClC-a-GFP*,* this Slit protein trap was derived from *Mi(MIC)sli****^03825^*** by the Gene Disruption Project.

**Antibody generation**

Immune sera against synthetic peptides from *Drosophila melanogaster* ClC-a (RVIDMSPEDQKQWEL, corresponding to amino acids 874-888, and ESKQSPSADKSNTENGNHA, corresponding to the last 19 amino acids of the protein, 1031-1049) were raised in rabbits using the services provided by Eurogentec. Peptides were coupled to keyhole limpet hemocyanin via a cysteine residue that had been added to the C-terminal of the peptide. After four/five boosts of immunization, the antisera were affinity purified using the peptide covalently coupled to Sulpholink (ThermoScientific). The polyclonal antibody was tested by immunoblotting in HEK293A cells transfected with the pcDNA3.1 vector (Invitrogen) expressing the ClC-a channel with a 3xFLAG-tag fused to the C-terminus.

**Western blots**

Two different protein extraction procedures were used. For the antibody testing, HEK293A cells were grown on DMEM containing 10% (v/v) fetal bovine serum (Sigma) and 1% penicillin/streptomycin at 37ºC in a humidity controlled incubator with 10% CO_2_. Cells were transfected with 2 µg of the *ClC-a 3xFLAG* pcDNA3.1 construct using the Transfectin reagent (BioRad). Forty-eight hours after transfection, cells were harvested and homogenized in lysis buffer containing 1% TX-100, 150 mM NaCl, and phosphate-buffered saline plus protease inhibitors as described elsewhere (Capdevila-Nortes et al., 2013). In the case of fly tissue protein extracts, twenty *Drosophila* heads were homogenized by 20 strokes in an Eppendorf Teflonglass homogenizer in 10 µl/brain RIPA buffer containing 50 mM TRIS/HCl pH8, 150 mM NaCl, 1% NP40, 0.5% deoxycholate, 0.1% SDS, 2 mM EDTA, and protease inhibitors (aprotinin (1 μg/ml), PMSF (174.2 μg/ml), Leupeptin (1 μg/ml), and Pepstatin (1 μg/ml)). For both extracts, the proteins in the supernatant were quantified by the BCA method. Sodium dodecyl sulfate polyacrylamide gel electrophoresis and western blot were performed as described elsewhere (Teijido et al., 2004) loading 50 µg of *Drosophila* head extracts, or 100 µg of HEK293A cell extracts. We used our custom polyclonal antibody to detect ClC-a (1:100 dilution), mouse monoclonal anti-Flag M2 (Sigma-Aldrich) (1:500 dilution), and anti-Tubulin (MMS-435P, Covance Antibody Products, Princeton, NJ) (1:500 dilution). Secondary antibodies were horseradish peroxidase-conjugated anti-rabbit and anti-mouse (Jackson ImmunoResearch).

**EdU labeling**

For EdU labeling dissected mid L3 larval brains were incubated for 15 min in 20μM EdU (Click-iT EdU Imaging Kit, Invitrogen) in Schneider medium at room temperature, and after washes fixed in ice cold 4% PFA with 0.1% Triton for one hour. Brains where then incubated with Click-iT Kit components at room temperature for 40 min, washed and immunostained with anti-DE cadherin to locate the OPC. EdU was visualized by detection with Alexa Fluor Azide 555 and nuclei with TOPRO-3.

**Quantifications**

To quantify photoreceptor targeting defects (Supplementary Figure 5J) single photoreceptor axons were followed to determine their layer selection.

To quantify the number of cortex glia nuclei in late L3 OLs, we manually counted *ClC-a*^+^ nuclei (Supplementary Figure 6A). Cortex glia nuclei present an average size of approximately 44.5 μm^2^ (Morante, Vallejo, Desplan, & Dominguez, 2013) and are clearly distinguishable from ClC-a^+^ ensheathing glia nuclei by their size and position in the OL. The *n* for this experiment was 8 brains.

To count the number of neuroblasts in the CB part of the hemisphere (Supplementary Figure 6B), we manually identified them based on their nuclear size and on Dpn and Mira antibody staining. Neuroblasts were distinguishable from mature INPs, which are also Dpn^+^ and Mira^+^, by their smaller nuclear size and higher intensity of TOPRO-3 nuclear staining. The *n* for this experiment was 8 brains.

To quantify the EdU labeling (Supplementary Figure 8) we imaged the OPC and counted the EdU^+^ nuclei present in 30μm of depth in control and mutant animals (n=5 brains each). Since the OPC is smaller in mutants compared to controls we normalized the number of EdU^+^ cells to the number of cells present in those 30 μm.

To differentiate DL1 from DL2, we used *gcm-lacZ*, which specifically labels the DL2 lineage (Supplementary Figure 10). The *n* for this experiment was between 11 and 12 brains.

**Statistics**

Statistical analysis was carried out following the same guidelines as in the main text. *p*-values for Supplementary Figures 6A, B; Supplementary Figure 8A, B; Supplementary Figure 11B were calculated with the non –parametric Mann-Whitney’s test. *p*-values for Supplementary Figure 10H were calculated with the Wilcoxon matched-pairs squad rank test.

**Supplementary Figure legends**

**Supplementary Figure 1. Comparative analysis of *ClC-a* expression patterns with antibody and various reporters.**

(A-C) Detection of *ClC-a* expression (green) in stellate cells of adult Malpighian tubules using anti-ClC-a antibody (A), the *ClC-a-GFP* protein trap (B), and the *ClC-a-GAL4* driver line combined with a membrane reporter (green) (C). Nuclei labeled with TOPRO-3 where indicated. (D-I) Detection of *ClC-a* expression in late L3 brain hemispheres. (D-F) Horizontal views of the surface of brain hemispheres. Antibody staining (D), protein trap (E), and driver (F) show the same expression patterns. Asterisks mark some neuroblast chambers. (G-I) Horizontal views deeper in hemispheres, in the optic lobe area. Arrowheads point to ClC-a expression between the LPC and lopn. (G) Antibody staining shows expression on the OPC, in the LF, and in between the LPC and lopn. (H) In addition, the protein trap construct also reveals expression deeper in the brain, around the IPC and forming a mesh-like structure inside the hemisphere, where the antibody did not penetrate. Inset shows expression between the LPC and lopn. Anti-E-cad staining (magenta) was used to identify the neuroepithelial cells and anti-Chaoptin (gray) label photoreceptors. (I) The *ClC-a-GAL4* driver mediated membrane labeling (green) pattern is very similar to the one observed with the antibody and the protein trap construct, including the signal detected between the LPC and lopn. Glial nuclei were labeled with anti-Repo antibody (blue). Not all glial nuclei are ClC-a^+^ (red). (J-K) *ClC-a-GFP* protein trap expression surrounding type I (J-J’’) and type II (K-K’’) neuroblasts labeled with anti-Dpn antibody (blue). (J-J’’) Confocal sections at different levels of a type I neuroblast (arrow) show the presence of ClC-a-GFP protein surrounding it. (K-K’’) Confocal sections at different levels of a type II neuroblast (arrow) show the presence of INPs (asterisks) also labeled with anti-Dpn. ClC-a-GFP is seen surrounding the neuroblast and delineating the chamber encasing the INPs and the neuroblast lineage.

CB, central brain; OL, optic lobe; LF, lamina furrow; LPC, lamina precursor cells; lopn, lobula plate neurons; OPC, outer proliferation center; IPC, inner proliferation center. Scale bars represent 10 µm.

**Supplementary Figure 2. Identification of *ClC-a* expressing glia.**

Confocal sections showing *ClC-a* expression pattern in the late L3 nervous system (A-D), and the optic lobe in pupal stages (E, F) and adult (G). *ClC-a* specific GAL4 driver was used to label cellular membranes (green) and nuclei (red) of ClC-a^+^ cells. Glial nuclei were labeled with anti-Repo antibody (blue) and photoreceptor cells with anti-Chaoptin (gray). (A) Larval brain where, besides a ClC-a^+^ signal in cortex glia both in brain hemispheres and the VNC, a ClC-a^+^ signal is detected in neuropil-ensheathing glia in the VNC, tract-ensheathing glia in connectives between the two hemispheres, and in peripheral nerves. (B,C) Cross section (B) and longitudinal section (C) of peripheral nerves containing ClC-a^+^ glia. Dashed line outlines the nerve. (D) Image of the optic stalk, which connects the eye disc and the optic lobe. ClC-a^+^ glia wraps this bundle formed by photoreceptor axons on their way to the optic lobe. Photoreceptor cell bodies are seen in the eye disc in gray and their axons in the optic lobe. Photoreceptors do not express *ClC-a*. (E, F) Based on the ClC-a^+^ Repo^+^ nucleus position, we can identify the following as *ClC-a* expressing glia: cxg, wg/dsg, Xg_o_, Xg_i_, mneg, and lopneg in 20 (E) and 50 (F) hrs After Pupal Formation (APF). (G) *ClC-a* expression is maintained in the adult. Signal in the medulla and lobula neuropils belongs to mneg and lopneg described projections into these structures.

egt, tract-ensheathing glia; egn, neuropil-ensheathing glia; wg, wrapping glia; pn, peripheral nerve; ed, eye disc; os, optic stalk; OPC, outer proliferation center; LPC, lamina precursor cells; BBB, blood brain barrier; ; cxg, cortex glia; megn, medulla neuropil-ensheathing glia; ep, epithelial glia; mg, marginal glia; Xg_o_, outer chiasm glia; Xg_i_, inner chiasm glia; psg, proximal satellite glia; wg/dsg, wrapping glia/distal satellite glia; lopegn, lobula plate neuropil-ensheathing glia. Scale bars represent 10 µm.

**Supplementary Figure 3. Immunohistochemistry and western blot analysis of *ClC-a* MiMIC alleles.**

(A, B) Anti-ClC-a antibody staining of adult Malpighian tubules in control animals (A) and *14007/Df* mutants (B). (C, D) Anti-ClC-a antibody staining of late L3 brains in control animals (C) and *14007/Df* mutants (D). Photoreceptors are labeled with anti-Chaoptin (green). (E) Western blot of protein extraction from HEK293 cells transfected with or without *ClC-a isoform C 3xFlag* pcDNA3.1. Both anti-Flag and anti-ClC-a antibodies detect a band below 130 kDa, which is possibly the weight of the protein (Uniprot prediction at 118 kDa) plus glycosylation. (F) Western blot of protein extraction from adult heads of controls and different allelic combinations. The signal around the 130 kDa mark reflects the presence of ClC-a protein in controls, most probably of different isoforms which range from 113 to 132 predicted kDa plus glycosylation. A strong reduction in this signal is observed in mutant animals.

Scale bars represent 10 µm.

**Supplementary Figure 4. Analysis of eye development in *ClC-a* mutants and *ClC-a* requirement in the eye.**

(A-C) Images of adult eyes of controls (A, B) and a *ClC-a* mutant allelic combination (C). In all cases ommatidia are stereotypically arranged. (D-I) Confocal images of developing ommatidia in control (D, F, H) and mutant (E, G, I) eye discs. The R7 is marked by anti-Prospero antibody; the anti-Boss antibody labels this R8 specific receptor, and the anti-Cut antibody labels the cone cells. No differences between the control and mutant expression patterns are observed. Together with the wild type external eye morphology this data show that eye development is normal in *ClC-a* mutants. (J-M) Analysis of *ClC-a* expression (red) at different stages of eye development. In the eye disc (J) and 40 hrs APF retina (K, L) photoreceptors are labeled with anti-Chaoptin (green). In the adult retina (M), photoreceptor rhabdomeres are labeled with Phalloidin. Nuclei are marked with TOPRO-3 where specified. Anti-ClC-a antibody does not label the eye tissue at any of the stages analyzed. (N-R) Assessment of *ClC-a* requirement in the eye. (N-P) Representative confocal sections of photoreceptor arrays (green) of control (N) and *ClC-a* mutant (O) eyes generated by the EGUF/hid technique and quantification of brains with phenotype (P). (Q, R) Quantification of the percentage of brains with different strengths of guidance phenotypes in photoreceptor-specific knock down (Q) and rescue experiments (R). Consistent with the absence of *ClC-a* expression in photoreceptors, misguidance phenotypes are non-autonomous. Hence, eye specific *ClC-a* knockout and knockdown results in proper photoreceptor guidance and eye specific *ClC-a* expression in mutants does not rescue photoreceptor guidance phenotypes.

Scale bars represent 10 µm.

**Supplementary Figure 5. Analysis of non-autonomous layer selection defects in misguided photoreceptors of *ClC-a* mutants.**

Confocal images of adult (A-G) and pupal (H,I) optic lobes stained with anti-Chaoptin to label all photoreceptors (green). Photoreceptor subtypes were labeled using cell type specific opsin reporters: R1-6 (magenta), R7 (blue), and R8 (red). An R8 specific driver *(senseless)* was used to label R8s in pupal brains (red). (A) Control photoreceptor array showing R1-6 photoreceptors stopping in the lamina. (B, C) Mutant arrays. (B) In mutant animals with weak guidance defects, R1-6 terminate normally in the lamina. (C) In animals with strong guidance defects, R1-R6 axons invade the medulla as seen in the inset (C’). (D) Control array showing R7s terminating at the M6 layer. (E) Mutant array shows misguided R7s terminating in the M6 layer like controls. (F) Control array showing R8s terminating in the M3 layer. (G) Mutant array showing misguided R8s terminating in the M1 layer. (H) Control array at 40 hrs APF. R8 cells terminate in the prospective M1 layer at the top of the medulla. This is a temporary stop since in a second stage they actively extend to the M3 layer. (I) Misguided R8s in the mutant animal also terminate in the M1 layer; however, the adult phenotype suggests that these cells are unable to detach from this temporary layer and retract to the M3. (J) Quantification of adult targeting defects in misguided photoreceptors. Most R8s terminate in M1 (red) instead of at M3, while most R7s terminate correctly at M6. The limited number of R7 targeting defects can be explained by the fact that in pupal stages, R7s already extend to a deeper layer with their growth cones very close to their synaptic partners, and that the R7 axons grow by intercalation of ingrowing processes of other neurons. n=number of photoreceptors analyzed.

Scale bars represent 10 µm.

**Supplementary Figure 6. Quantification of ClC-a^+^ cortex glia nuclei and central brain neuroblasts in control and *ClC-a* mutant brain hemispheres.**

(A) Ratio of cortex glia nuclei/µm^3^ in late L3 control (*05423^ClC-a-GAL4^/+*) and mutant (*05423^ClC-a-GAL4^/14007*) brain hemispheres. (B) Quantification of the number of CB neuroblasts present in late L3 control (*14007/+*) and mutant (*14007/Df*) brain hemispheres.

n.s.>0.05, *p<0.05.

**Supplementary Figure 7. Study of neuroepithelium to neuroblast transition in *ClC-a* mutant animals.**

Lateral views of volume-rendering 3D reconstructions of late L3 larval hemispheres. (A) Control animal (*14007/+*) stained with anti-E-cad (gray, A’) labeling the OPC and anti-Dpn (red, A”) labeling neuroblasts, which are differentiating on the medial side of the OPC. Double arrow marks the width of the OPC in the central region. (B, C) Examples of OPC defects observed in late L3 mutant hemispheres (*14007/Df*). (B) E-cad staining (B’) reveals a reduction in the width of the OPC (double arrow), especially in the central part. (C) In this severe example, although E-cad staining is gone (bracket), there are neuroblasts, suggesting that the neuroepithelial to neuroblast transition in this region of the OPC took place prematurely and there is no more OPC tissue. (D) Control animal (*14007/+*) stained with anti-L’sc (green), which labels the neuroepithelial cell that will transition to neuroblast, and anti-Dpn (red) to visualize neuroblasts. (E) Mutant animal that lacked L’sc expression in the central region of the OPC. The absence of L’sc indicates that there was no more neuroepithelium to differentiate into neuroblasts. The presence of neuroblasts (red) in the region where L’sc is missing indicates that there used to be neuroepithelium. It is worth noting that, in addition to premature neuroepithelium to neuroblast transition, neurons generated by the d-IPC could also contribute to this phenotype by occupying this space. Invasion of these neurons could be a consequence of the disruption of the glial barrier, a phenotype described in Figure 5.

OPC, outer proliferation center; LPC, lamina precursos cells; lopn, lobula plate neurons. Scale bars represent 10 µm.

**Supplementary Figure 8. Comparison of EdU labeling in control and *ClC-a* mutant OPC neuroepitheliums.**

(A-B) Normalized quantification of OPC neuroepithelial EdU-labeled cells in control and mutant animals. The S-phase starts as a wave from one side of the nucleus, and hence, depending on S-phase progression during the incubation time, EdU labeling appears as a crescent for cells in early S-phase or it fills the whole nucleus in cells that reach the late S-phase. (A) Ratio of *EdU^+^ OPC cells*/*total OPC cells*. No differences were observed between control and mutant animals. (B) Ratio of *late S-phase EdU^+^ OPC cells /total EdU^+^ OPC cells*. This ratio was lower in mutants than in controls. (C, D) Representative confocal sections of EdU-labelled neuroepithelial OPC cells in control (C) and mutant (D) animals. E-cad signal (green) labels neuroepithelial cells and TOPRO-3 (blue) nuclei. Asterisks mark cells that have incorporated EdU (red) in the whole nucleus while arrowheads mark cells were EdU signal is restricted to part of the nucleus. This images and the quantification show that during the incubation time frame (15 minutes), less cells achieve complete EdU incorporation in mutants compared to controls, which would be in accordance with impaired proliferation and smaller clones.

Scale bars represent 10 µm. n.s.>0.05, * p<0.05

**Supplementary Figure 9. Characterization of a surface-associated cortex glia and cortex glia-specific driver.**

Expression patterns of *mir-8-GAL4* driver and *mir-8 ^cxg^*. Membranes were labeled in green, nuclei in red, and all glial nuclei in blue (anti-Repo). (A, B) Horizontal views at the surface of the central brain showing *mir-8* (A) and *mir-8 ^cxg^* expression (B). (A’, B’) Magnifications of dashed region of interest in (A) and (B). (A’) *mir-8-GAL4* is expressed in neuroblasts (arrowhead) and neurons (arrow). The gain of the red channel has been increased to visualize nuclear signal in neuroblasts and neurons. (B’) Magnification of dashed region of interest in (B). Using the same gain as in (A’), neuronal and neuroblast labeling is gone using the *mir-8 ^cxg^* transgenes. (C, D) Horizontal views deep in the brain hemisphere showing *mir-8-GAL4* (C) and *mir-8 ^cxg^* expression (D). (C) Neuronal *mir-8* expression is seen in the mushroom body calyx. Xg_o_ glia do not express *mir-8*. (D) No neuronal expression was detected in the calyx or Xg_o_. (E) Frontal view of a volume-rendering 3D reconstruction of a mid L3 optic lobe. No membrane (green) and/or nuclear (red) signal between the OPC and IPC confirmed that *mir-8 ^cxg^* was not expressed in boundary glia.

CB, central brain; OL, optic lobe; cxg, cortex glia; LF, lamina furrow; OPC, outer proliferation center; IPC, inner proliferation center; Xg_o_, outer chiasm glia; Ca, calyx. Scale bars represent 10 µm.

**Supplementary Figure 10. Developmental details of the formation of the glial barrier between the LPC and the lopn.**

(A-C) Characterization of cell types in the barrier. Specific drivers were used to label membranes in green or red. Glial nuclei are labeled with anti-Repo (blue). (A-B) Horizontal views of early (A) and late (B) optic lobes showing ClC-a^-^ satellite glia population membranes labeled with the *R43H01-LexA* specific driver in red and ClC-a^+^ membranes (*05423^ClC-a-GAL4^* */+*) in green. (C) Horizontal view of a late L3 optic lobe showing Xg_o_ and palisade glia membranes labeled with the specific driver *R25A01-GAL4* in green. This driver is not expressed at earlier developmental time points, and thus cannot be used to manipulate these cell types when they group together as boundary glia before photoreceptor innervation in mid L3. (D-F) DL1 lineage tracing to analyze parallelisms between the timing of visualization of DL1 derived Xg_o_ glia and visualization of ClC-a^+^ boundary glia (prospective Xg_o_ and pag) in the optic lobe. DL1 lineage (green) is visualized with the DL1 specific driver *R38H02-GAL4*, which is expressed in this NB early in development in a short time window, and the G-TRACE system. Optic lobes were stained with anti-E-cad (magenta) to identify neuroepithelial cells and anti-Repo (blue) to identify glial cells. (D) Frontal view of volume-rendering 3D reconstructions of a wild type early L3 optic lobe showing DL1 progeny (green) in the same region as ClC-a^+^ cells in Figure 5A. Neuroepithelia were segmented and the rest of the signal masked to avoid background noise and allow better visualization. (E) Horizontal view of a confocal plane showing the neural progeny of the DL1 lineage in the central brain (Repo^-^) and the Xg_o_ and Xg_i_ glial progeny in the optic lobe (Repo^+^). (F) Frontal view of a volume-rendering 3D reconstruction of a wild type mid/late L3 brain showing DL1 progeny (green) in the same region as *ClC-a*^+^ cells in Figure 5B. (G, H) *ClC-a* lineage tracing, performed with the *05423^ClC-a-GAL4^* driver and the G-TRACE system, to analyze the drop in ClC-a^+^ cells from mid to late L3. Anti-Repo was used to label glial cells. (G) Frontal view of a volume-rendering 3D reconstruction of a late L3 brain. (G’) G-TRACE green labeling indicates that cells expressed *ClC-a* at some point during development. (G”) G-TRACE red labeling shows cells currently expressing *ClC-a*. Bracket and arrowhead respectively demarcate Xg_o_ and pag that had already expressed *ClC-a* in early and mid L3 (K’), but downregulated *ClC-a* expression in late L3 (K”). (H) Green box plots show the number of nuclei per brain that expressed *ClC-a* at a developmental time prior to the larval stage analyzed. Red box plots show the number of nuclei per brain that currently express *ClC-a*. Dots in box plots represent data points. Comparisons between green and red box plots are shown for each developmental time analyzed. Both in mid and late L3, there are more cells that used to express *ClC-a* than cells that currently express it, indicating that some downregulation of *ClC-a* expression is occurring at mid and late L3 stages. The number of nuclei currently expressing *ClC-a* in mid L3 quantified with *05423^ClC-a-GAL4^* mediated G-TRACE (*UAS-nsl-DsRed*) is lower than the number of cells observed when using *05423^ClC-a-GAL4^* and *UAS-H2B-RFP*, presumably due to the use of different UAS reporters*.* Compare mid L3 current G-TRACE value (red box plot) in (H) to the value in *05423^ClC-a-GAL4^ /+* animals at mid L3 in Figure 5M. (I, J) Assessment of cell death in mutants in the region where boundary glia would normally be positioned. Early L3 (I) and mid L3 (J) mutant brains showing, as expected, few (I) or no (J) ClC-a^+^ boundary glia nuclei (red). Most of the sporadic Dcp-1 signal observed (arrowheads, gray) is in non-glial cells (repo^-^).

OPC, outer proliferation center; IPC, inner proliferation center; sg, satellite glia; bg, boundary glia; pag, palisade glia; Xg_o_, outer chiasm glia; Xg_i_, inner chiasm glia; cxg, cortex glia. Scale bars represent 10 µm. n.s.>0.05, * p<0.05, ** p<0.01.

**Supplementary Figure 11. DL1/DL2 distinction based on *gcm* expression.**

(A-A”) Confocal sections showing the progeny of DL1 and DL2 neuroblasts labeled by R9D11-tdtom expression (red). *gcm-lacZ* expression (green, A’) labels part of the DL2 lineage (arrow). INPs (arrowheads) are labeled in anti-Deadpan antibody (gray, A”). (B) Quantification and comparison of the number of INPs per lineage. Scale bars represent 10 µm. ***p<0.001.

**Supplementary Figure 12. Comparison of *slit-*LacZ (*sli ^05428^*) and Slit-GFP (*sli[MI03825-GFSTF.2]*) expression patterns.**

*sli ^05428^* is a commonly used nuclear lacZ reporter of *slit* expression. We characterized Slit-GFP expression pattern because *sli ^05428^* nuclear LacZ expression at early and mid L3 stages was very low and difficult to distinguish from background. Slit full-length protein can be cleaved into large N-terminal (Slit-N) and short C-terminal (Slit-C) fragments. Slit-FL and Slit-N are more tightly associated with the cell surface, whereas Slit-C is mostly shed into the extracellular space (Brose et al., 1999). The GFP tag in this Slit-GFP reporter line was located between amino acids 398-399, in the second LRR repeat, so in the Slit-N terminal fragment. Thus, the GFP signal of the Slit-GFP reporter stays in the membrane of the *slit* expressing cells. *slit* signal for both *slit*-lacZ and Slit-GFP reporters is shown in red. *ClC-a*^+^ membranes are labeled with *05423^ClC-a-GAL4^*/*UAS-mCD8-mRFP* and shown in green. Glia nuclei are labeled with anti-Repo antibody (blue). (A, B) Horizontal views through the VNC showing nuclear LacZ signal (red, A, A’) and membrane Slit-GFP signal (red, B, B’) in midline glia. (C, D) Frontal views of late L3 optic lobes. (C) Nuclear LacZ signal (red) can be seen in Xg_o_ and medulla neurons as previously reported (Suzuki et al., 2016; Tayler et al., 2004), as well as in cortex glia. In early pupal stages, LacZ expression in Xg_o_ is stronger than in late L3 (data not shown). (D) Membrane Slit-GFP signal (red) is seen in the same cell types as *slit*-LacZ: Xg_o_, cortex glia, and medulla neurons. Thus, the Slit-GFP expression pattern is the same as the one observed with *slit*-lacZ.

midg, midline glia; sa-cxg, surface-associated cortex glia; pag, palisade glia; Xg_o_, outer chiasm glia; mn, medulla neuron. Scale bars represent 10 µm.

**References**

Brose K, Bland KS, Hong Wang K, Arnott D, Henzel W, Goodman CS, Tessier-Lavigne M, Kidd T. 1999. Slit Proteins Bind Robo Receptors and Have an Evolutionarily Conserved Role in Repulsive Axon Guidance and vertebrates, midline cells also appear to express counterbalancing inhibitory cues that push axons away (reviewed in Tessier-Lavigne and Goodman, 19. *Cell* **96**:795–806.

Capdevila-Nortes X, López-Hernández T, Apaja PM, de Heredia ML, Sirisi S, Callejo G, Arnedo T, Nunes V, Lukacs GL, Gasull X, Estévez R. 2013. Insights into MLC pathogenesis: GlialCAM is an MLC1 chaperone required for proper activation of volume-regulated anion currents. *Hum Mol Genet* **22**:4405–4416. doi:10.1093/hmg/ddt290

Suzuki T, Hasegawa E, Nakai Y, Kaido M, Takayama R, Sato M. 2016. Formation of Neuronal Circuits by Interactions between Neuronal Populations Derived from Different Origins in the Drosophila Visual Center. *Cell Rep* **15**:499–509. doi:10.1016/j.celrep.2016.03.056

Tayler T, Robixaux M, Garrity P. 2004. Compartmentalization of visual centers in the Drosophila brain requires Slit and Robo proteins. *Development* **131**:5935–5945. doi:10.1242/dev.01465

Teijido O, Martínez A, Pusch M, Zorzano A, Soriano E, del Río JA, Palacín M, Estévez R. 2004. Localization and functional analyses of the MLC1 protein involved in megalencephalic leukoencephalopathy with subcortical cysts. *Hum Mol Genet* **13**:2581–2594. doi:10.1093/hmg/ddh291

**GENOTYPE LIST**

**Figure 1:**

**A, B, C, D, E, F, G, H, I, J.** *w; UAS-mCD8-GFP/+; 05423^ClC-a-GAL4^/UAS-H2B-RFP*

**K.** *w; mir8-GAL4/UAS-mCD8-GFP; +/+*

**L.** *w; UAS-mCD8-GFP/+; R54H02-GAL4/UAS-H2B-RFP*

**M, N.** *w; UAS-mCD8-GFP, lexAop-CD2-RFP/+; 05423^ClC-a-GAL4^/wrapper932i-LexA*

**O, P, Q.** *w; UAS-mCD8-GFP/+; 05423^ClC-a-GAL4^/wrapper932i-Gal80*

**Figure 2:**

**C.**

*+/+: w^1118^; +/+; +/+*

*05423^ClC-a-GAL4^/+: w; +/+; 05423^ClC-aGAL4^/+*

*14007/+: w; +/+; 14007/+*

*Df/+: w; +/+; Df(3R)PS2/+*

*05423^ClC-a-GAL4^/14007: w; +/+; 05423^ClC-aGAL4^/14007*

*05423^ClC-a-GAL4^/Df: w; +/+; 05423^ClC-aGAL4^/Df(3R)PS2*

**D.**

*UAS-ClC-a/+: w; UAS-ClC-a/+; +/+*

*UAS-ClCN2/+: w; UAS-ClCN2/+; +/+*

*05423^ClC-a-GAL4^/Df & +/+: w; +/+; 05423^ClC-a-GAL4^/Df(3R)PS2*

*05423^ClC-a-GAL4^/Df & UAS-ClC-a/+: w; UAS-ClC-a/+; 05423^ClC-a-GAL4^/Df(3R)PS2*

*05423^ClC-a-GAL4^/Df & UAS-ClCN2/+: w; UAS-rClC-2/+; 05423^ClC-a-GAL4^/Df(3R)PS2*

**E.**

*14007/+: w; +/+; 14007/+*

**F.**

*14007/Df: w; +/+; 14007/Df(3R)PS2*

**G.**

*05423^ClC-a-GAL4^/+: w; +/+; 05423^ClC-a-GAL4^/+*

*05423/+: w; +/+; 05423/+*

*14007/+: w; +/+; 14007/+*

*Df/+: w; +/+; Df(3R)PS2/+*

*05423^ClC-a-GAL4^/Df: w; +/+; 05423^ClC-a-GAL4^/Df(3R)PS2*

*05423/Df: w; +/+; 05423/Df(3R)PS2*

*14007/Df: w; +/+; 14007/Df(3R)PS2*

*05423^ClC-a-GAL4^/14007: w; +/+; 05423^ClC-a-GAL4^/14007*

*05423/14007: w; +/+; 05423/14007*

*14007/14007: w; +/+; 14007/14007*

**H.**

*Repo-GAL4/+: w; +/+; Repo-Gal4/+*

*05423^ClC-a-GAL4^/+: w; +/+; 05423^ClC-a-GAL4^/+*

*UAS-ClC-aRNAi/+: w; UAS-ClC-aRNAi/+; UAS-Dcr2/+*

*Repo>ClC-aRNAi: w; UAS-ClC-aRNAi /+; Repo-GAL4/UAS-Dcr2*

*05423^ClC-a-GAL4^>ClC-aRNAi: w; UAS-ClC-aRNAi /+; 05423^ClC-a-GAL4^/UAS-Dcr2*

**I.**

*Repo-GAL4/+ & 14007/Df(3R)PS2: w; Repo-GAL4/+; 14007/Df(3R)PS2*

*UAS-ClC-a/+ & 14007/Df(3R)PS2: w; UAS-ClC-a/+; 14007/Df(3R)PS2*

*UAS-ClCN2/+ & 14007/Df(3R)PS2: w; UAS-ClCN2/+; 14007/Df(3R)PS2*

*Repo>ClC-a & 14007/Df(3R)PS2: w; UAS-ClC-a/Repo-GAL4; 14007/Df(3R)PS2*

*Repo>ClCN2 & 14007/Df(3R)PS2: w; UAS-ClCN2/Repo-GAL4; 14007/Df(3R)PS2*

*05423^ClC-a-GAL4^/Df & +/+: w; +/+; 05423^ClC-a-GAL4^/Df(3R)PS2*

*05423^ClC-a-GAL4^/Df & UAS-ClC-a/+: w; UAS-ClC-a/+; 05423^ClC-a-GAL4^/Df(3R)PS2*

*05423^ClC-a-GAL4^/Df & UAS-ClCN2/+: w; UAS-rClC-2/+; 05423^ClC-a-GAL4^/Df(3R)PS2*

**J.**

*Repo>ClC-aRNAi: w; UAS-ClC-aRNAi /+; Repo-GAL4/UAS-Dcr2*

**K.**

*05423^ClC-a-GAL4^>ClC-aRNAi: w; UAS-ClC-aRNAi /+; 05423^ClC-a-GAL4^/UAS-Dcr2*

**L, M.**

*14007/+: w; +/+; 14007/+*

*Df/+: w; +/+; Df(3R)PS2/+*

*14007/Df: w; +/+; 14007/Df(3R)PS2*

**Figure 3:**

**A, B, C, D.**

*05423^ClC-a-GAL4^/+: w; UAS-mCD8-GFP/+; 05423^ClC-a-GAL4^/UAS-H2B-RFP*

**E, F, G, H.**

*05423^ClC-a-GAL4^/14007: w; UAS-mCD8-RFP/UAS-H2B-YFP; 05423^ClC-aGAL4^/14007*

**Figure 4:**

**A, B, C, F, G.**

*14007/+: w; +/+; 14007/+*

*14007/Df: w; +/+; 14007/Df(3R)PS2*

**D, E.**

*14007/+: hs-FLP, tub-Gal80, FRT19A/FRT19A; tub-GAL4,UAS-mCD8-GFP /+; 14007/+*

*14007/Df: hs-FLP, tub-Gal80, FRT19A/FRT19A; tub-GAL4,UAS-mCD8-GFP /+; 14007/Df(3R)PS2*

**H, I.**

*+/+: w; +/+; +/+*

*14007/+: w; +/+; 14007/+*

*Df/+: w; +/+; Df(3R)PS2/+*

*14007/Df: w; +/+; 14007/Df(3R)PS2*

**J, K.**

*mir8^cxg^ or UAS-ClC-a control: tub>Gal80>/+; mir8-GAL4/+; 14007/Df(3R)PS2 or tub>Gal80>/+; +/RepoFLP6.2, UAS-ClC-a; 14007/Df(3R)PS2*

*mir8^cxg^>ClC-a: tub>Gal80>/+; mir8-GAL4/RepoFLP6.2, UAS-ClC-a; 14007/Df(3R)PS2*

**Figure 5:**

**A, B, C, D, E, F.**

*05423^ClC-a-GAL4^/+: w; UAS-mCD8-GFP/+; 05423^ClC-a-GAL4^/UAS-H2B-RFP*

**G, H, I, J, K, L.**

*05423^ClC-a-GAL4^/14007: w; UAS-mCD8-RFP/UAS-H2B-YFP; 05423^ClC-aGAL4^/14007*

**M.**

*05423^ClC-a-GAL4^/+: w; UAS-mCD8-RFP/ UAS-H2B-YFP; 05423^ClC-aGAL4^/+*

*05423^ClC-a-GAL4^/14007: w; UAS-mCD8-RFP/ UAS-H2B-YFP; 05423^ClC-aGAL4^/14007*

**Figure 6:**

**B.**

*14007/+: w; +/+; 14007, R9D11-tdtomato/+*

**C, D.**

*14007/Df: w; +/+; 14007, R9D11-tdtomato/Df(3R)PS2*

**E, G, H.**

*14007/+: w; UAS-G-TRACE/+; R38H02-GAL4, 14007/+*

**F, G, H.**

*14007/Df: w; UAS-G-TRACE/+; R38H02-GAL4, 14007/ Df(3R)PS2*

**I, J, L.**

*05423^ClC-a-GAL4^/+: w; UAS-mCD8-GFP/+; 05423^ClC-aGAL4^/R9D11-tdtomato*

**K,L.**

*05423^ClC-a-GAL4^/14007: w; UAS-mCD8-GFP/+; 05423^ClC-aGAL4^/14007, R9D11-tdtomato*

**Figure 7:**

**A, B, C, D.**

*05423^ClC-a-GAL4^/+: w; UAS-mCD8-GFP/+; 05423^ClC-a-GAL4^/UAS-H2B-RFP*

**E, F.**

*14007/Df: w; +/+; 14007/Df(3R)PS2*

**G, H.**

*slit^dui^ /* *slit^dui^: w; slit^dui^, GMR-GFP/slit^dui^, GMR-GFP; +/+*

**I, J, K.** *w; UAS-mCD8-RFP/Slit-GFP; 05423^ClC-a-GAL4^/+*

**L.**

*14007/+: w; +/+; 14007/+*

*slit^dui^ /+: w; slit^dui^, GMR-GFP/+; 14007/+*

*14007/14007: w; +/+; 14007/14007*

*slit^dui^ /+; 14007/14007: w; slit^dui^, GMR-GFP/+; 14007/14007*

**M.**

*05423^ClC-a-GAL4^/+: w; +/+; 05423^ClC-a-GAL4^/+*

*UAS-slitRNAi/+: w; UAS-slitRNAi/+; UAS-Dcr2/+*

*05423^ClC-a-GAL4^>slitRNAi: w; UAS-slitRNAi/+; UAS-Dcr2/05423^ClC-a-GAL4^*

*slit^dui^/+; 05423^ClC-a-GAL4^>slitRNAi: w; UAS-SlitRNAi/ slit^dui^, GMR-GFP; UAS-Dcr2/05423^ClC-a-GAL4^*

**Figure 8:**

**B.**

*14007/+: w; +/+; 14007/+*

*14007/Df: w; +/+; 14007/Df(3R)PS2*

*14007/Df, mir8^cxg^>ClC-a: tub>Gal80>/+; mir8-GAL4/RepoFLP6:2, UAS-ClC-a;14007/Df(3R)PS2*

**C.**

*mir8^cxg^ or UAS-ClC-a control: tub>Gal80>/+; mir8-GAL4/+; 14007/Df(3R)PS2 or tub>Gal80>/+; +/RepoFLP6.2, UAS-ClC-a; 14007/Df(3R)PS2*

*mir8^cxg^>ClC-a: tub>Gal80>/+; mir8-GAL4/RepoFLP6.2, UASClC-a; 14007/Df(3R)PS2*

**Figure 9:**

**B, D.**

*05423^ClC-a-GAL4^/+: w; UAS-mCD8-GFP/+; 05423^ClC-aGAL4^/+*

**C.**

*05423^ClC-a-GAL4^/+: w; +/+; 05423^ClC-aGAL4^/+*

**E.**

*14007/+: w; +/+; 14007/+*

**F, H.**

*05423^ClC-a-GAL4^/14007: w; UAS-mCD8-GFP/+; 05423^ClC-aGAL4^/14007*

**G.**

*05423^ClC-a-GAL4^/14007: w; +/+; 05423^ClC-aGAL4^/14007*

**I.**

*14007/Df: w; +/+; 14007/Df(3R)PS2*

**K, L.**

*14007/+: hs-FLP, tub-Gal80, FRT19A/FRT19A; tub-GAL4,UAS-mCD8-GFP/+; 14007/+*

**M, N, O.**

*14007/Df: hs-FLP, tub-Gal80, FRT19A/FRT19A; tub-GAL4,UAS-mCD8-GFP/+; 14007/Df(3R)PS2*

**Supplementary figure 1:**

**A, D, G.** *w; +/+; +/+*

**B, E, H.** *w; +/+; ClC-aGFP/ClC-aGFP*

**C, F.** *w; UAS-mCD8-GFP/+; 05423^ClC-aGAL4^/+*

**I.** *w; UAS-mCD8-GFP/+; 05423^ClC-aGAL4^/UAS-H2B-RFP*

**Supplementary figure 2:**

**A, B, C, D, E, F, G.** *w; UAS-mCD8-GFP/+; 05423^ClC-aGAL4^/UAS-H2B-RFP*

**Supplementary figure 3:**

**A, C.**

*+/+:* *w; +/+; +/+*

**B, D.**

*14007/Df: w; +/+; 14007/Df(3R)PS2*

**E.**

*+/+:* *w; +/+; +/+*

*Df/+: w; +/+; Df(3R)PS2/+*

*05423/+: w; +/+; 05423/+*

*14007/+: w; +/+; 14007/+*

*05423/Df: w; +/+; 05423/Df(3R)PS2*

*14007/Df: w; +/+; 14007/Df(3R)PS2*

*14007/05423: w; +/+; 14007/05423*

**Supplementary figure 4:**

**A, D, F, H.**

*14007/+: w; +/+; 14007/+*

**B.**

*Df/+: w; +/+; Df(3R)PS2/+*

**C, E, G, I.**

*14007/Df: w; +/+; 14007/Df(3R)PS2*

**J, K, L, M.** *w; +/+;+/+*

**N, P.** *FRT82B,GMR-hid,cl/FRT82B ry+*

**O, P***. FRT82B,GMR-hid,cl/FRT82B 05423*

**Q.**

*GMR-GAL4/+: w; GMR-GAL4/+; +/+*

*UAS-ClC-aRNAi/+: w; UAS-ClC-aRNAi/+; UAS-Dcr2/+*

*GMR>ClC-aRNAi: w; UAS-ClC-aRNAi/GMR-GAL4; UAS-Dcr2/+*

**R.**

*GMR-GAL4/+, 14007/Df: w; GMR-GAL4/+; 14007/Df(3R)PS2*

*UAS-ClC-a/+, 14007/Df: w; UAS-ClC-a/+; 14007/Df(3R)PS2*

*GMR>ClC-a, 14007/Df: w; GMR-GAL4/UAS-ClC-a; 14007/Df(3R)PS2*

**Supplementary figure 5:**

**A.** *w; Rh1-GAL4/UAS-mCD8-GFP; 14007/+*

**B, C.** *w; Rh1-GAL4/UAS-mCD8-GFP; 14007/Df(3R)PS2*

**D.** *w; Rh4-EGFP/+; 14007/+*

**E.** *w; Rh4-EGFP/+; 14007/Df(3R)PS2*

**F.** *w; Rh6-LacZ/+; 14007/+*

**G.** *w; Rh6-LacZ/+; 14007/Df(3R)PS2*

**H.** *w; sens-GAL4, UAS-utr-GFP/+; 14007/+*

**I.** *w; sens-GAL4, UAS-utr-GFP /+; 14007/Df(3R)PS2*

**J.** *w; Rh6-LacZ/ Rh4-EGFP; 14007/Df(3R)PS2*

**Supplementary figure 6:**

**A.**

*05423^ClC-a-GAL4^/+: w; UAS-mCD8-GFP/ UAS-H2B-YFP; 05423^ClC-a-GAL4^/+*

*05423^ClC-a-GAL4^/14007: w; UAS-mCD-8RFP/ UAS-H2B-YFP; 05423^ClC-a-GAL4^/14007*

**B.**

*14007/+: w; +/+; 14007/+*

*14007/Df: w; +/+; 14007/Df(3R)PS2*

**Supplementary figure 7:**

**A, D.**

*14007/+: w; +/+; 14007/+*

**B, C, E.**

*14007/Df: w; +/+; 14007/Df(3R)PS2*

**Supplementary figure 8:**

**A, B, C.**

*14007/+: w; +/+; 14007/+*

**A, B, D.**

*14007/Df: w; +/+; 14007/Df(3R)PS2*

**Supplementary figure 9:**

**A, C.** *w; mir8-GAL4/UAS-mCD8-GFP; +/+*

**B, D, E.** *tub>GAL80>/+; mir8-GAL4, RepoFLP6.2/+; UAS-mCD8-GFP/+*

**Supplementary figure 10:**

**A, B.** *w; UAS-mCD8-GFP, lexAop-CD2-RFP/R43H01-LexA; 05423^ClC-a-GAL4^/+*

**C.** *w; UAS-mCD8-GFP/+; R25A01-GAL4/+*

**D, E, F.** *w; UAS-G-TRACE/+; R38H02-GAL4/+*

**G, H.** *w; UAS-G-TRACE/+; 05423^ClC-a-GAL4^/+*

**I, J**

*05423^ClC-a-GAL4^/14007: w; UAS-H2B-YFP/+; 05423^ClC-a-GAL4^/14007*

**Supplementary figure 11:**

**A.** *w; gcm-lacZ/+; R9D11-tdtomato/+*

**Supplementary figure 12:**

**A, C.** *w; slit-lacZ/+; +/+*

**B, D.** *w; slit-GFP/UAS-mCD8-RFP; 05423^ClC-a-GAL4^/+*
