# Supplementary figures and images for: Drosophila ClC‐a is required in glia of the stem cell niche for proper neurogenesis and wiring of neural circuits
Source: Glia. 2019 Sep 3;67(12):2374–98. doi: 10.1002/glia.23691 (PMC6851788; doi:10.1002/glia.23691)

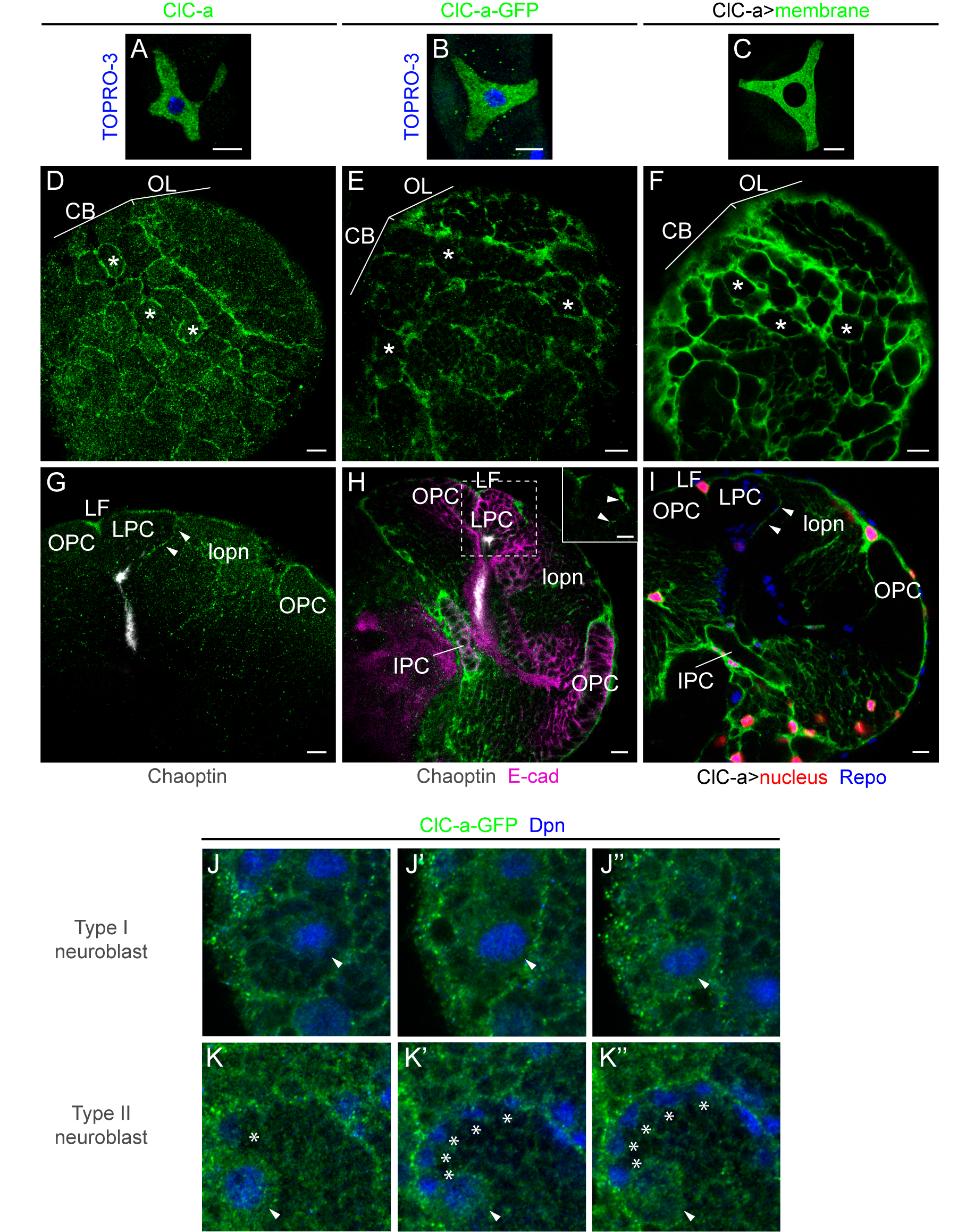

Supplement: Supplementary file 2 — Supplementary Figure 1. Comparative analysis of ClC‐a expression patterns with antibody and various reporters. (A‐C) Detection of ClC‐a expression (green) in stellate cells of adult Malpighian tubules using anti‐ClC‐a antibody (A), the ClC‐a‐GFP protein trap (B), and the ClC‐a‐GAL4 driver line combined with a membrane reporter (green) (C). Nuclei labeled with TOPRO‐3 where indicated.(D‐I) Detection of ClC‐a expression in late L3 brain hemispheres. (D‐F) Horizontal views of the surface of brain hemispheres. Antibody staining (D), protein trap (E), and driver (F) show the same expression patterns. Asterisks mark some neuroblast chambers. (G‐I)Horizontal views deeper in hemispheres, in the optic lobe area. Arrowheads point to ClC‐a expression between the LPC and lopn. (G) Antibody staining shows expression on the OPC, in the LF, and in between the LPC and lopn. (H) In addition, the protein trap construct also reveals expression deeper in the brain, around the IPC and forming a mesh‐like structure inside the hemisphere, where the antibody did not penetrate. Inset shows expression between the LPC and lopn. Anti‐E‐cad staining (magenta) was used to identify the neuroepithelial cells and anti‐Chaoptin (gray) label photoreceptors. (I) The ClC‐a‐GAL4 driver mediated membrane labeling (green) pattern is very similar to the one observed with the antibody and the protein trap construct, including the signal detected between the LPC and lopn. Glial nuclei werel abeled with anti‐Repo antibody (blue). Not all glial nuclei are ClC‐a+(red). (J‐K) ClC‐a‐GFP protein trap expression surrounding type I (J‐J’’) and type II (K‐K’’) neuroblasts labeled with anti‐Dpn antibody (blue). (J‐J’’) Confocal sections at different levels of a type I neuroblast (arrow) show the presence of ClC‐a‐GFP protein surroundingit. (K‐K’’) Confocal sections at different levels of a type II neuroblast (arrow) show the presence of INPs (asterisks) also labeled with anti‐Dpn.ClC‐a‐GFP is seen surrounding the neur [file GLIA-67-2374-s003.tif]

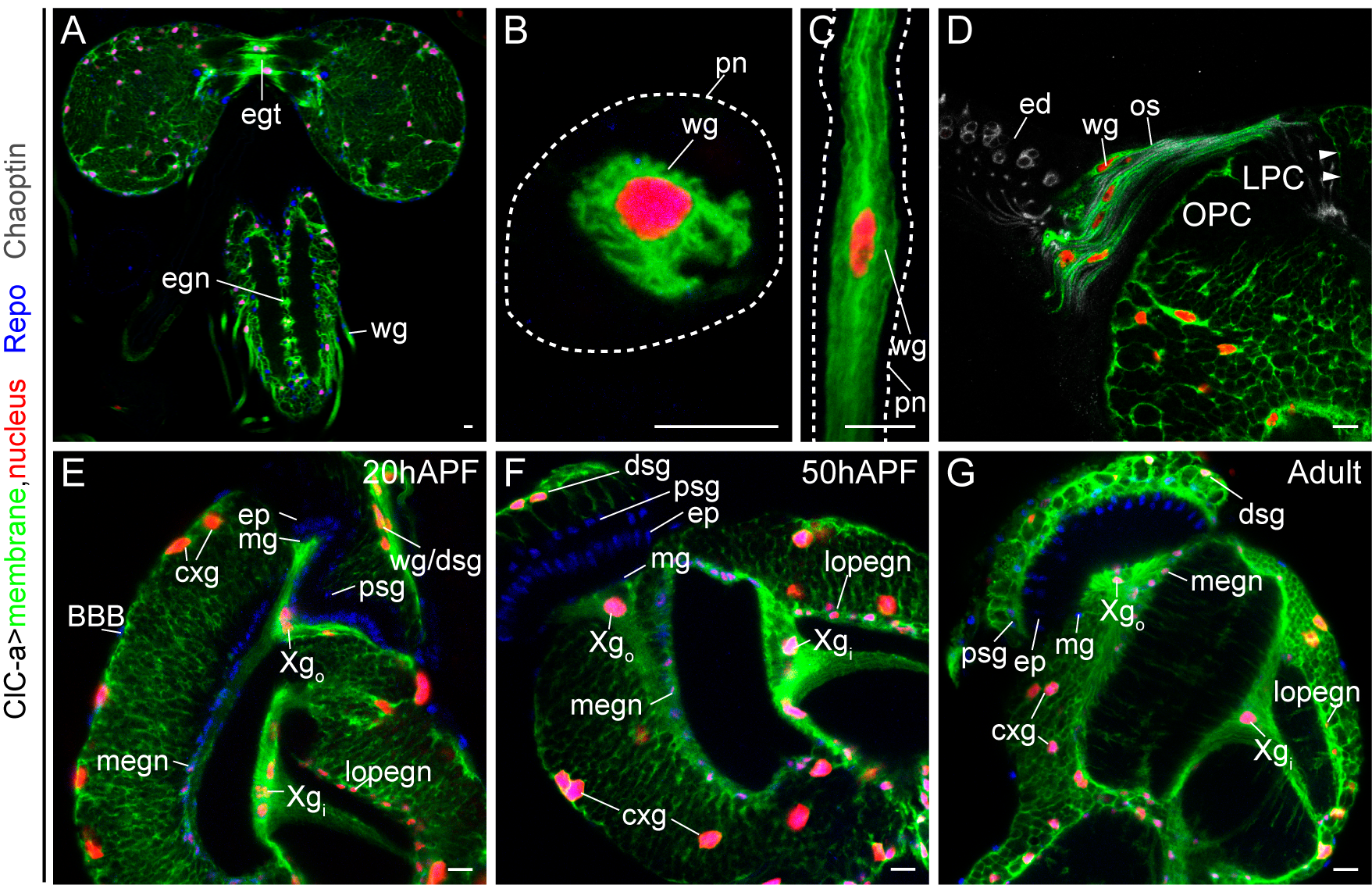

Supplement: Supplementary file 3 — Supplementary Figure 2. Identification of ClC‐a expressing glia. Confocal sections showing ClC‐a expression pattern in the late L3 nervous system (A‐D), and the optic lobe in pupal stages (E, F) and adult (G). ClC‐a specific GAL4 driver was used to label cellular membranes (green) and nuclei (red) of ClC‐a+ cells. Glial nuclei were labeled with anti‐Repo antibody (blue) and photoreceptor cells with anti‐Chaoptin (gray). (A) Larval brain where, besides a ClC‐a+ signal in cortex glia both in brain hemispheres and the VNC, a ClC‐a+ signal is detected in neuropil‐ensheathing glia in the VNC, tract‐ensheathing glia in connectives between the two hemispheres, and in peripheral nerves. (B,C) Cross section (B) and longitudinal section (C) of peripheral nerves containing ClC‐a+ glia. Dashed line outlines the nerve. (D) Image of the optic stalk, which connects the eye disc and the optic lobe. ClC‐a+ glia wraps this bundle formed by photoreceptor axons on their way to the optic lobe. Photoreceptor cell bodies are seen in the eye disc in gray and their axons in the optic lobe. Photoreceptors do not express ClC‐a. (E, F) Based on the ClC‐a+ Repo+ nucleus position, we can identify the following as ClC‐a expressing glia: cxg, wg/dsg, Xgo, Xgi,mneg, and lopneg in 20 (E) and 50 (F) hrs After Pupal Formation (APF). (G) ClC‐a expression is maintained in the adult. Signal in the medulla and lobula neuropils belongs to mneg and lopneg described projections into these structures. egt, tract‐ensheathing glia; egn, neuropil‐ensheathing glia; wg, wrapping glia; pn, peripheral nerve; ed, eye disc; os, optic stalk; OPC, outer proliferation center; LPC, lamina precursor cells; BBB, blood brain barrier; cxg, cortex glia; megn, medulla neuropil‐ensheathing glia; ep, epithelial glia; mg, marginal glia; Xgo, outer chiasm glia; Xgi, inner chiasm glia; psg, proximal satellite glia; wg/dsg, wrapping glia/distal satellite glia; lopegn, lobula plate neuropil‐ensheathing glia. Scale bars represent 10 µm [file GLIA-67-2374-s004.tif]

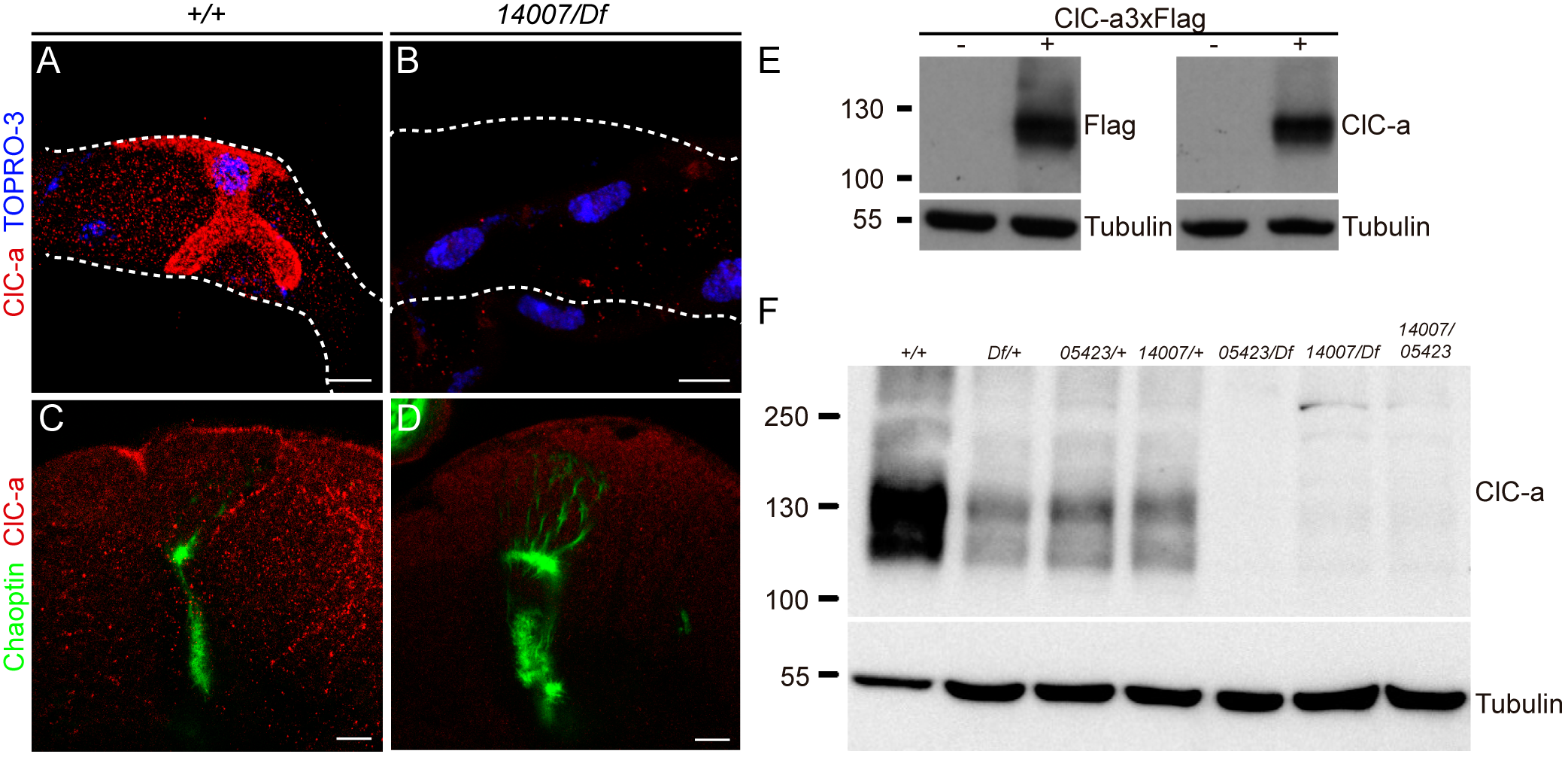

Supplement: Supplementary file 4 — Supplementary Figure 3. Immunohistochemistry and western blot analysis of ClC‐a MiMIC alleles. (A, B) Anti‐ClC‐a antibody staining of adult Malpighian tubules in control animals (A) and 14007/Df mutants (B). (C, D) Anti‐ClC‐a antibody staining of late L3 brains in control animals (C) and 14007/Df mutants (D). Photoreceptors are labeled with anti‐Chaoptin (green).(E) Western blot of protein extraction from HEK293 cells transfected with or without ClC‐a isoform C 3xFlag pcDNA3.1. Both anti‐Flag and anti‐ClC‐a antibodies detect a band below 130 kDa, which is possibly the weight of the protein (Uniprot prediction at 118 kDa) plus glycosylation. (F) Western blot of protein extraction from adult heads of controls and different allelic combinations. The signal around the 130 kDa mark reflects the presence of ClC‐a protein in controls, most probably of different isoforms which range from 113 to 132 predicted kDa plus glycosylation. A strong reduction in this signal is observed in mutant animals. Scale bars represent 10 μm. [file GLIA-67-2374-s005.tif]

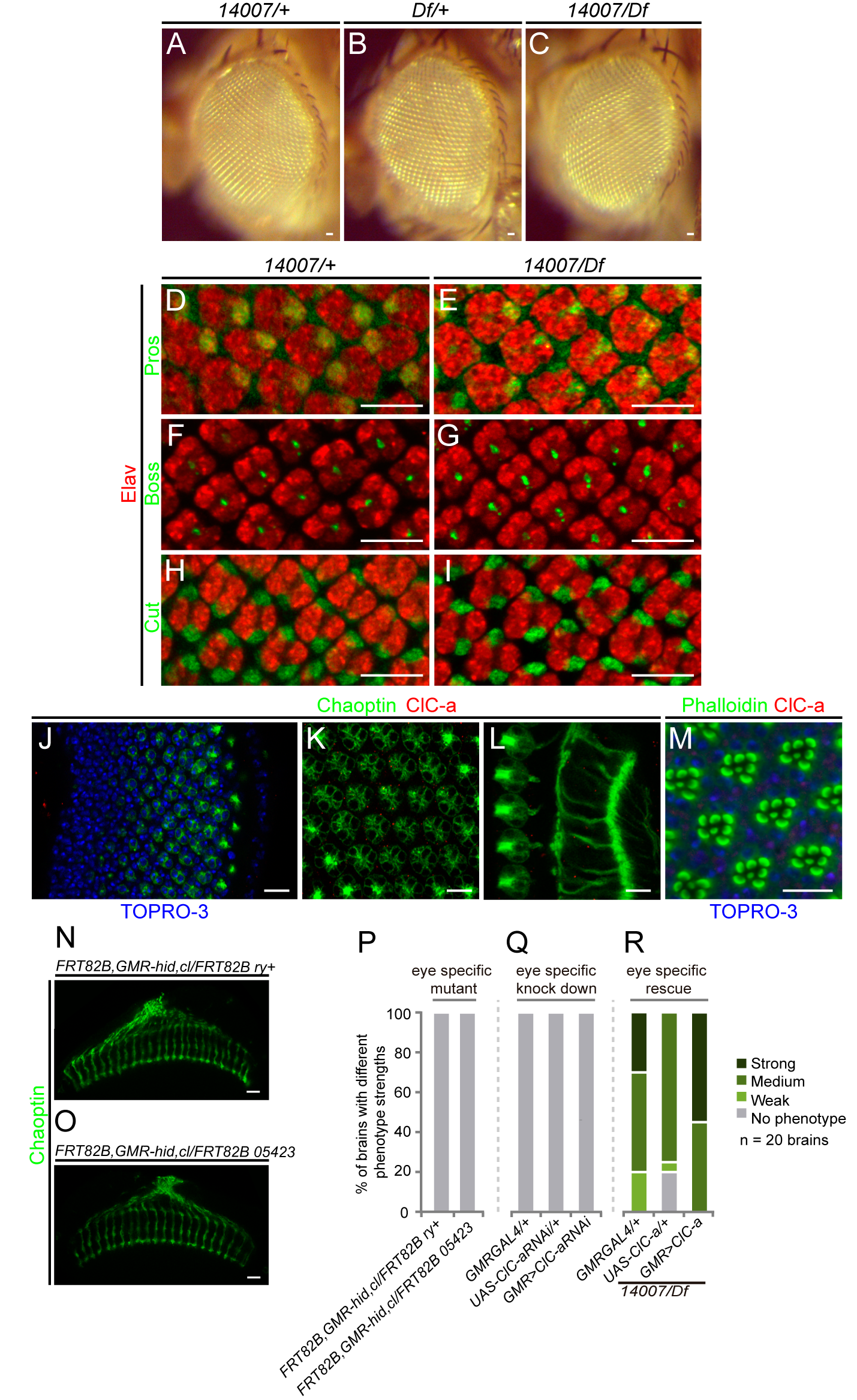

Supplement: Supplementary file 5 — Supplementary Figure 4. Analysis of eye development in ClC‐a mutants and ClC‐a requirement in the eye. (A‐C) Images of adult eyes of controls (A, B) and a ClC‐a mutant allelic combination (C). In all cases ommatidia are stereotypically arranged. (D‐I) Confocal images of developing ommatidia in control (D, F, H) and mutant (E, G, I) eye discs. The R7 is marked by anti‐Prospero antibody; the anti‐Boss antibody labels this R8 specific receptor, and the anti‐Cut antibody labels the cone cells. No differences between the control and mutant expression patterns are observed. Together with the wild type external eye morphology this data show that eye development is normal in ClC‐a mutants. (J‐M) Analysis of ClC‐a expression (red) at different stages of eye development. In the eye disc (J) and 40 hrs APF retina (K, L) photoreceptors are labeled with anti‐Chaoptin (green). In the adult retina (M), photoreceptor rhabdomeres are labeled with Phalloidin. Nuclei are marked with TOPRO‐3 where specified. Anti‐ClC‐a antibody does not label the eye tissue at any of the stages analyzed. (N‐R) Assessment of ClC‐a requirement in the eye. (N‐P) Representative confocal sections of photoreceptor arrays (green) of control (N) and ClC‐a mutant (O) eyes generated by the EGUF/hid technique and quantification of brains with phenotype (P). (Q, R) Quantification of the percentage of brains with different strengths of guidance phenotypes in photoreceptor‐specific knock down (Q) and rescue experiments (R). Consistent with the absence of ClC‐a expressionin photoreceptors, misguidance phenotypes are non‐autonomous. Hence, eye specific ClC‐a knockout and knockdown results in proper photoreceptor guidance and eye specific ClC‐a expression in mutants does not rescue photoreceptor guidance phenotypes. Scale bars represent 10 μm. [file GLIA-67-2374-s006.tif]

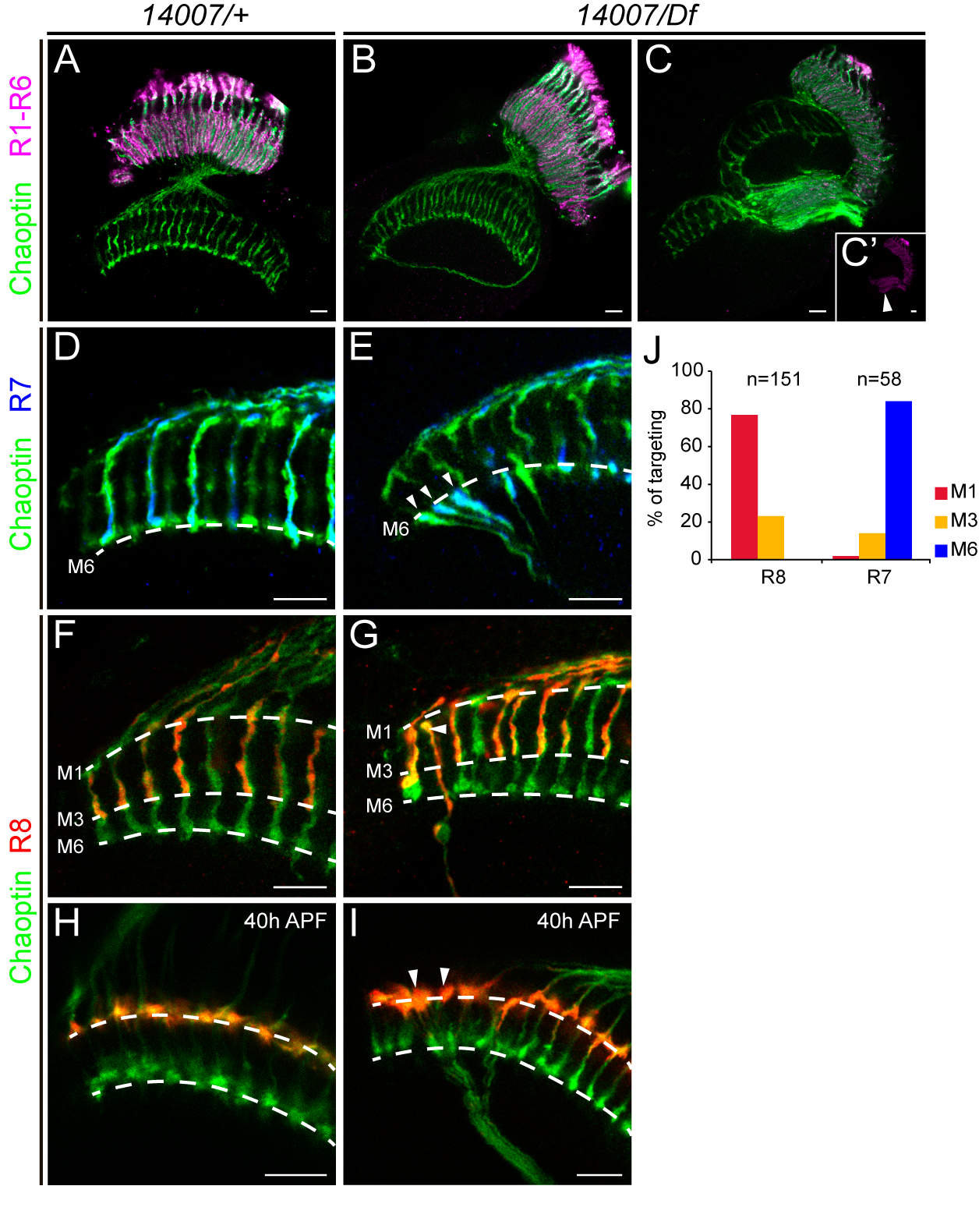

Supplement: Supplementary file 6 — Supplementary Figure 5. Analysis of non‐autonomous layer selection defects in misguided photoreceptors of ClC‐a mutants. Confocal images of adult (A‐G) and pupal (H,I) optic lobes stained with anti‐Chaoptin to label all photoreceptors (green). Photoreceptor subtypes were labeled using cell type specific opsin reporters: R1‐6 (magenta), R7 (blue), and R8 (red). An R8 specific driver (senseless) was used to label R8s in pupal brains (red). (A) Control photoreceptor array showing R1‐6 photoreceptors stopping in the lamina. (B, C) Mutant arrays. (B) In mutant animals with weak guidance defects, R1‐6 terminate normally in the lamina. (C) In animals with strong guidance defects, R1‐R6 axons invade the medulla as seen in the inset (C’). (D) Control array showing R7s terminating at the M6 layer. (E) Mutant array shows misguided R7s terminating in the M6 layer like controls. (F) Control array showing R8s terminating in the M3 layer. (G) Mutant array showing misguided R8s terminating in the M1 layer. (H) Control array at 40 hrs APF. R8 cells terminate in the prospective M1 layer at the top of the medulla. This is a temporary stop since in a second stage they actively extend to the M3 layer. (I) Misguided R8s in the mutant animal also terminate in the M1 layer; however, the adult phenotype suggests that these cells are unable to detach from this temporary layer and retract to the M3. (J) Quantification of adult targeting defects in misguided photoreceptors. Most R8s terminate in M1 (red) instead of at M3, while most R7s terminate correctly at M6. The limited number of R7 targeting defects can be explained by the fact that in pupal stages, R7s already extend to a deeper layer with their growth cones very close to their synaptic partners, and that the R7 axons grow by intercalation of ingrowing processes of other neurons. n=number of photoreceptors analyzed. Scale bars represent 10 µm. [file GLIA-67-2374-s007.tif]

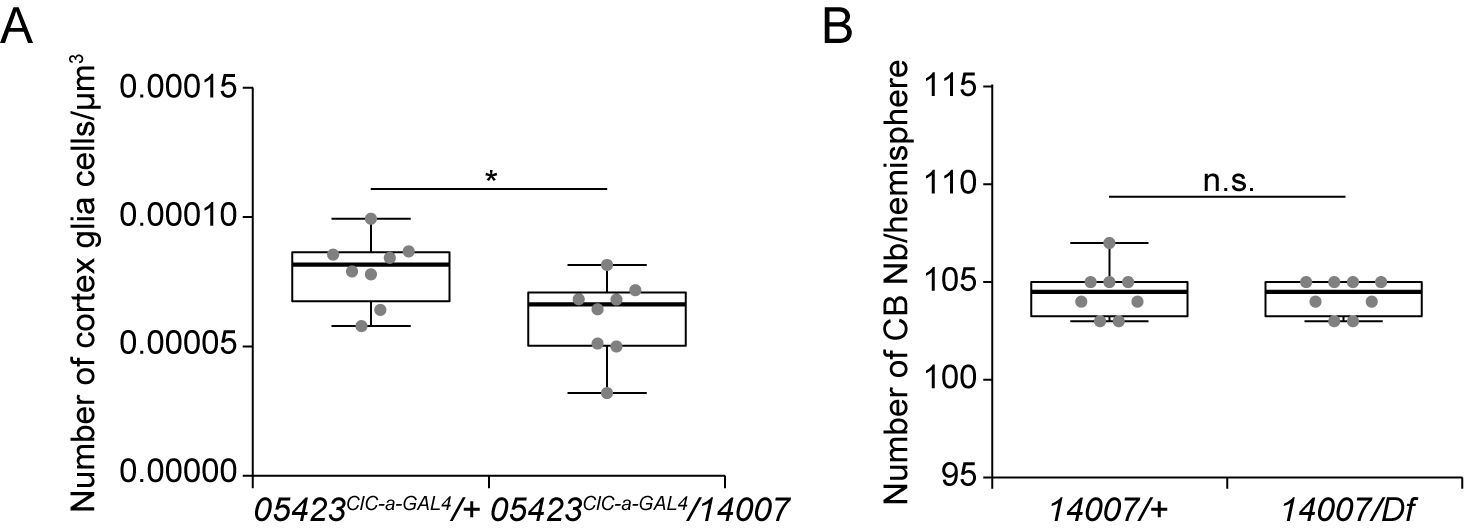

Supplement: Supplementary file 7 — Supplementary Figure 6. Quantification of ClC‐a+ cortex glia nuclei and central brain neuroblasts in control and ClC‐a mutant brain hemispheres. (A) Ratio of cortex glia nuclei/µm3 in late L3 control (05423ClC‐a‐GAL4/+) and mutant (05423ClC‐a‐GAL4/14007) brain hemispheres. (B) Quantification of the number of CB neuroblasts present in late L3 control (14007/+) and mutant (14007/Df) brain hemispheres. 1 n.s.>0.05, *p<0.05. [file GLIA-67-2374-s008.tif]

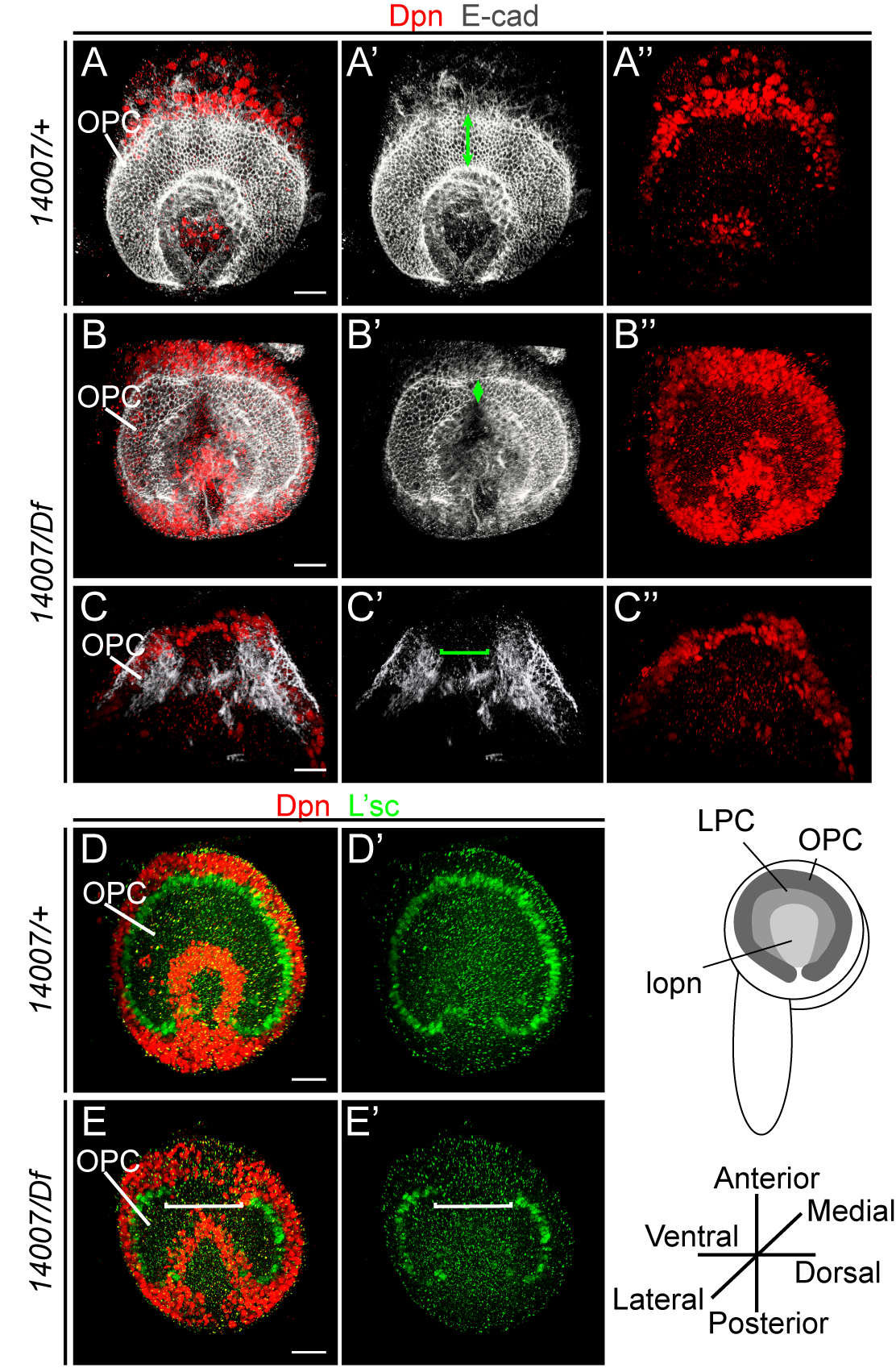

Supplement: Supplementary file 8 — Supplementary Figure 7. Study of neuroepithelium to neuroblast transition in ClC‐a mutant animals. Lateral views of volume‐rendering 3D reconstructions of late L3 larval hemispheres. (A) Control animal (14007/+) stained with anti‐E‐cad (gray, A’) labeling the OPC and anti‐Dpn (red, A”) labeling neuroblasts, which are differentiating on the medial side of the OPC. Double arrow marks the width of the OPC in the central region. (B, C) Examples of OPC defects observed in late L3 mutant hemispheres (14007/Df). (B) E‐cad staining (B’) reveals a reduction in the width of the OPC (double arrow), especially in the central part. (C) In this severe example, although E‐cad staining is gone (bracket), there are neuroblasts, suggesting that the neuroepithelial to neuroblast transition in this region of the OPC took place prematurely and there is no more OPC tissue. (D) Control animal (14007/+) stained with anti‐L'sc (green), which labels the neuroepithelial cell that will transition to neuroblast, and anti‐Dpn (red) to visualize neuroblasts. (E) Mutant animal that lacked L'sc expression in the central region of the OPC. The absence of L'sc indicates that there was no more neuroepithelium to differentiate into neuroblasts. The presence of neuroblasts (red) in the region where L'sc is missing indicates that there used to be neuroepithelium. It is worth noting that, in addition to premature neuroepithelium to neuroblast transition, neurons generated by the d‐IPC could also contribute to this phenotype by occupying this space. Invasion of these neurons could be a consequence of the disruptionof the glial barrier, a phenotype described in Figure 5. OPC, outer proliferation center; LPC, lamina precursos cells; lopn, lobula plate neurons. Scale bars represent 10 µm. [file GLIA-67-2374-s009.tif]

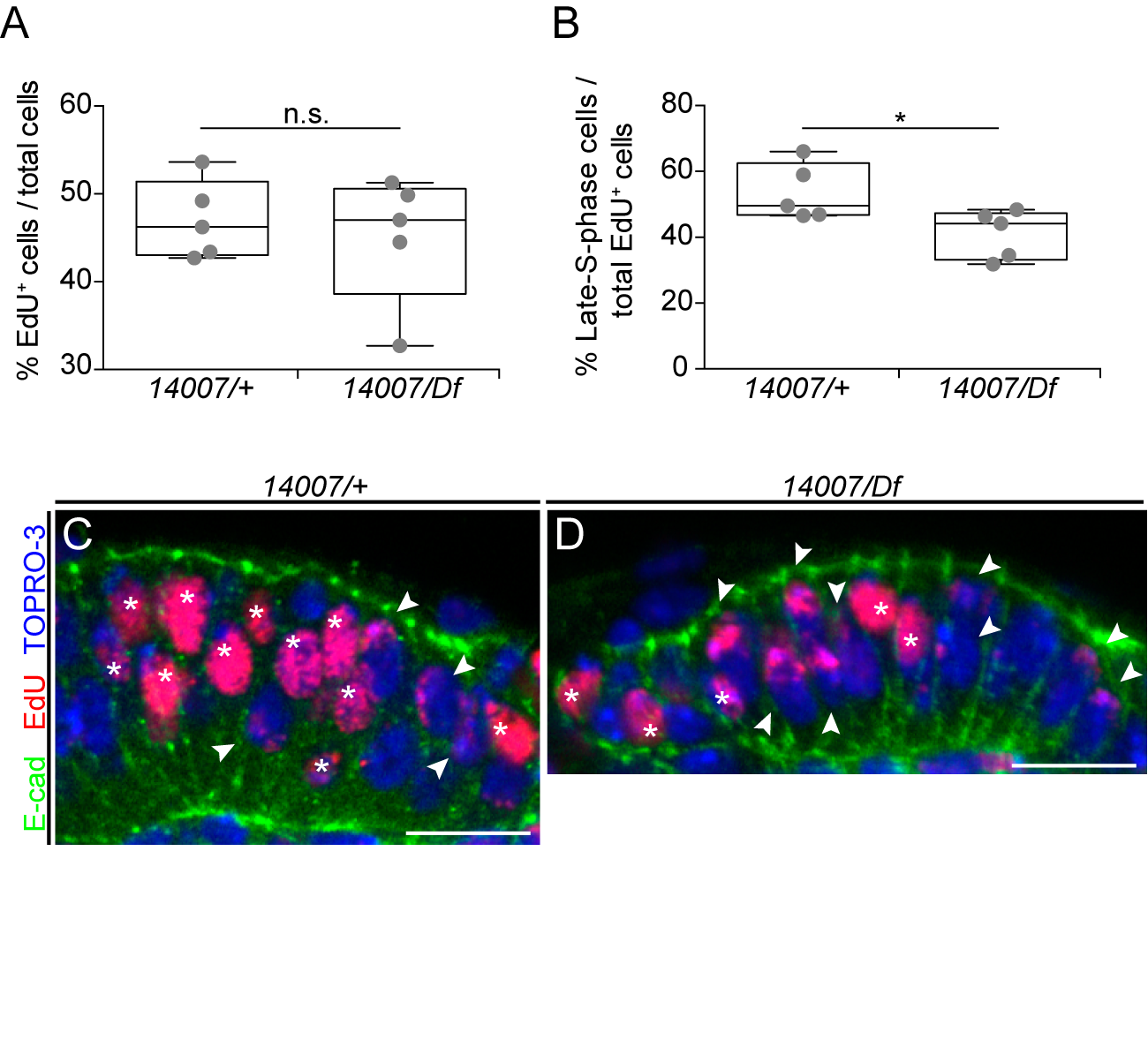

Supplement: Supplementary file 9 — Supplementary Figure 8. Comparison of EdU labeling in control and ClC‐a mutant OPC neuroepitheliums. (A‐B) Normalized quantification of OPC neuroepithelial EdU‐labeled cells in control and mutant animals. The S‐phase starts as a wave from one side of the nucleus, and hence, depending on S‐phase progression during the incubation time, EdU labeling appears as a crescent for cells in early S‐phase or it fills the whole nucleus in cells that reach the late S‐phase.(A) Ratio of EdU+ OPC cells/total OPC cells. No differences were observed between control and mutant animals. (B) Ratio of late S‐phase EdU+ OPC cells /total EdU+ OPC cells. This ratio was lower in mutants than in controls. (C, D) Representative confocal sections of EdU‐labelled neuroepithelial OPC cells in control (C) and mutant (D) animals. E‐cad signal (green) labels neuroepithelial cells and TOPRO‐3 (blue) nuclei. Asterisks mark cells that have incorporated EdU (red) in the whole nucleus while arrowheads mark cells were EdU signal is restricted to part of the nucleus. These images and the quantification show that during the incubation time frame (15 minutes), less cells achieve complete EdU incorporation in mutants compared to controls, which would be in accordance with impaired proliferation and smaller clones. Scale bars represent 10 µm. n.s.>0.05, * p<0.05. [file GLIA-67-2374-s010.tif]

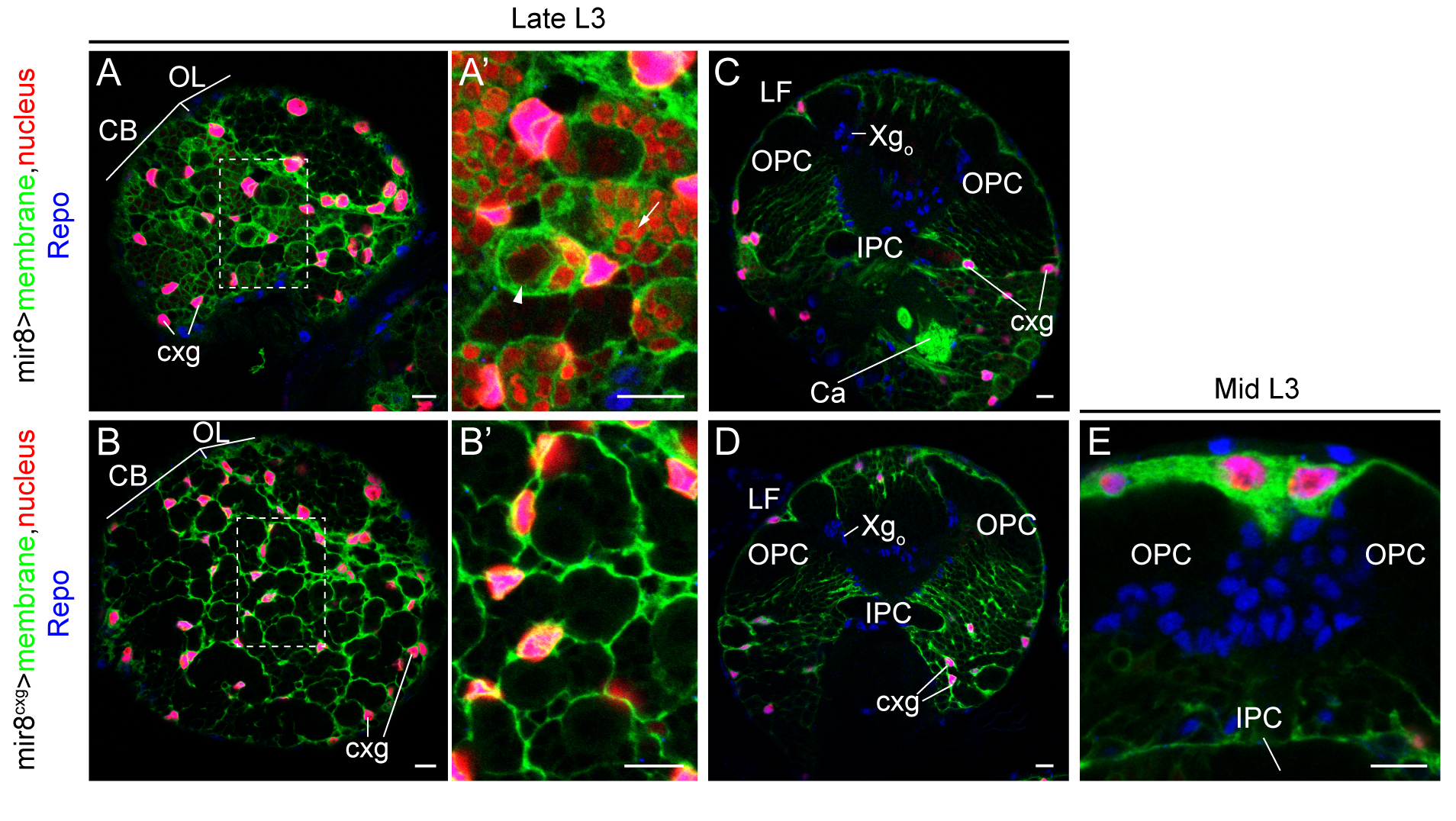

Supplement: Supplementary file 10 — Supplementary Figure 9. Characterization of a surface‐associated cortex glia and cortex glia‐specific driver. Expression patterns of mir‐8‐GAL4 driver and mir‐8 cxg. Membranes were labeled in green, nuclei in red, and all glial nuclei in blue (anti‐Repo). (A,B) Horizontal views at the surface of the central brain showing mir‐8 (A) and mir‐8 cxg expression (B). (A’, B’) Magnifications of dashed region of interest in (A) and (B). (A’) mir‐8‐GAL4 is expressed in neuroblasts (arrowhead) and neurons (arrow). The gain of the red channel has been increased to visualize nuclear signal in neuroblasts and neurons. (B’) Magnification of dashed region of interest in (B). Using the same gain as in (A’), neuronal and neuroblast labeling is gone using the mir‐8 cxg transgenes. (C, D) Horizontal views deep in the brain hemisphere showing mir‐8‐GAL4 (C) and mir‐8 cxg expression (D). (C) Neuronal mir‐8 expression is seen in the mushroombody calyx. Xgo glia do not express mir‐8. (D) No neuronal expression was detected in the calyx or Xgo.(E) Frontal view of a volume‐rendering 3D reconstruction of a mid L3 optic lobe. No membrane (green) and/or nuclear (red) signal between the OPC and IPC confirmed that mir‐8 cxg was not expressed in boundary glia. CB, central brain; OL, optic lobe; cxg, cortex glia; LF, lamina furrow; OPC, outer proliferation center; IPC, inner proliferation center; Xgo, outer chiasm glia; Ca, calyx. Scale bars represent 10 µm. [file GLIA-67-2374-s001.tif]

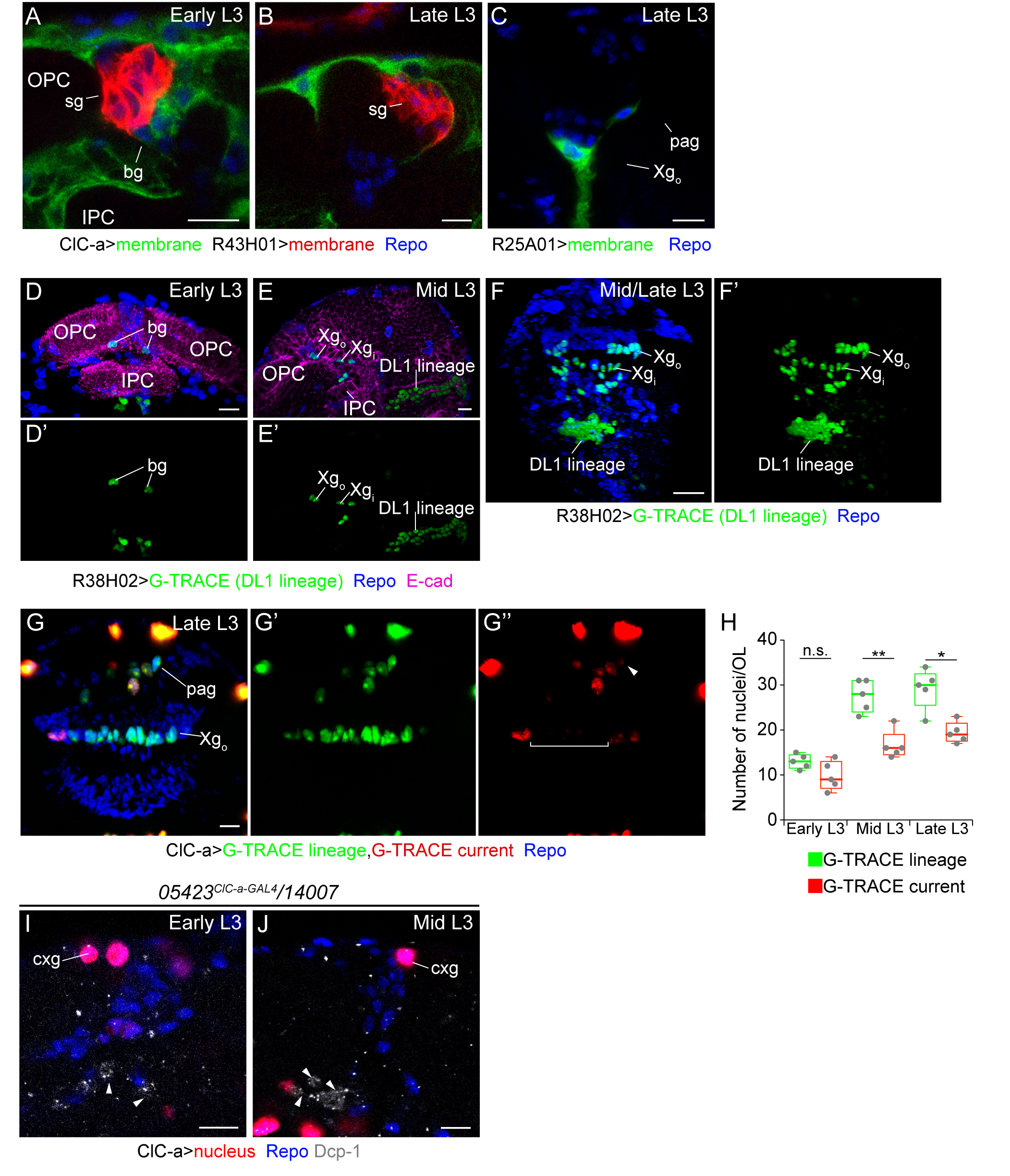

Supplement: Supplementary file 11 — Supplementary Figure 10. Developmental details of the formation of the glial barrier between the LPC and the lopn. (A‐C) Characterization of cell types in the barrier. Specific drivers were used to label membranes in green or red. Glial nuclei are labeled with anti‐Repo (blue). (A‐B) Horizontal views of early (A) and late (B) optic lobes showing ClC‐a− satellite glia population membranes labeled with the R43H01‐LexA specific driver in red and ClC‐a+ membranes (05423 ClC‐a‐GAL4 /+) in green. (C) Horizontal view of a late L3 optic lobe showing Xgo and palisade glia membranes labeled with the specific driver R25A01‐GAL4 in green. This driver is not expressed at earlier developmental time points, and thus cannot be used to manipulate these cell types when they group together as boundary glia before photoreceptor innervation in mid L3. (D‐F) DL1 lineage tracing to analyze parallelisms between the timing of visualization of DL1 derived Xgo glia and visualization of ClC‐a+ boundary glia (prospective Xgo and pag) in the optic lobe. DL1 lineage (green) is visualized with the DL1 specific driver R38H02‐GAL4, which is expressed in this NB early in development in a short time window, and the G‐TRACE system. Optic lobes were stained with anti‐E‐cad (magenta) to identify neuroepithelial cells and anti‐Repo (blue) to identify glial cells. (D) Frontal view of volume‐rendering 3D reconstructions of a wild type early L3 optic lobe showing DL1 progeny (green) in the same region as ClC‐a+ cells in Figure 5a. Neuroepithelia were segmented and the rest of the signal masked to avoid background noise and allow better visualization. (E) Horizontal view of a confocal plane showing the neural progeny of the DL1 lineage in the central brain (Repo−) and the Xgo and Xgi glial progeny in the optic lobe (Repo+). (F) Frontal view of a volume‐rendering 3D reconstruction of a wild type mid/late L3 brain showing DL1 progeny (green) in the same region as ClC‐a + cells in Figure 5b. (G, H) ClC‐a lineage [file GLIA-67-2374-s011.tif]

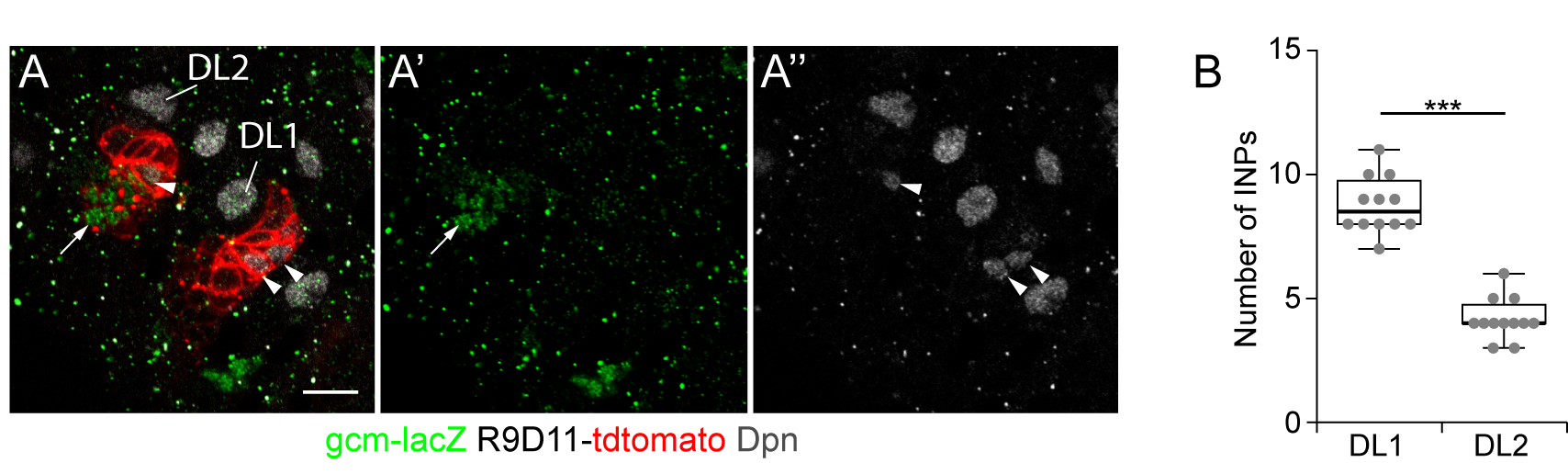

Supplement: Supplementary file 12 — Supplementary Figure 11. DL1/DL2 distinction based on gcm expression. (A‐A”) Confocal sections showing the progeny of DL1 and DL2 neuroblasts labeled by R9D11‐tdtom expression (red). gcm‐lacZ expression (green, A’) labels part of the DL2 lineage (arrow). INPs (arrowheads) are labeled in anti‐Deadpan antibody (gray, A”). (B) Quantification and comparison of the number of INPs per lineage. Scale bars represent 10 μm. ***p < .001 [file GLIA-67-2374-s012.tif]

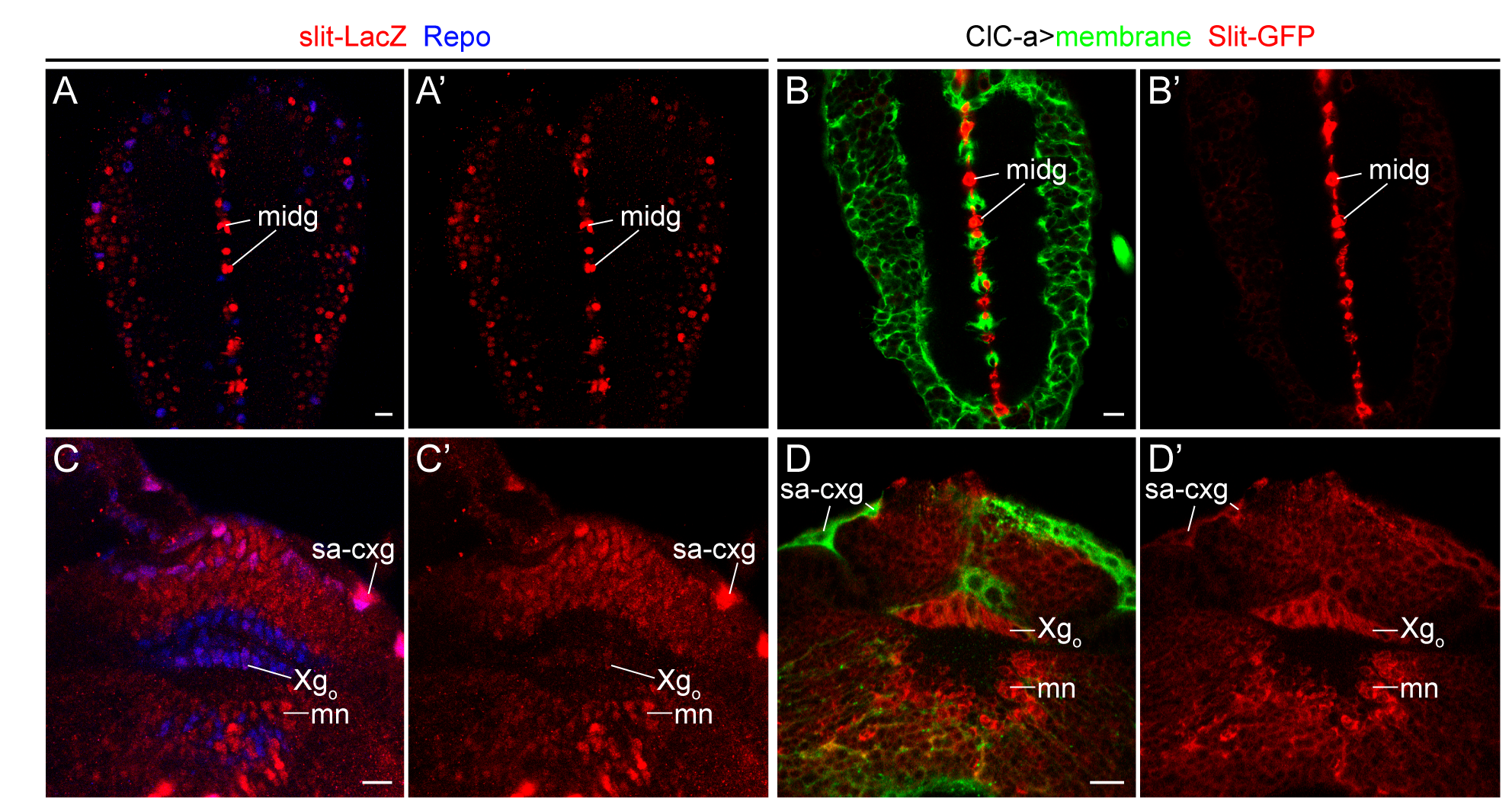

Supplement: Supplementary file 13 — Supplementary Figure 12. Comparison of slit‐LacZ (sli 05428 ) and Slit‐GFP (sli[MI03825‐GFSTF.2]) expression patterns. sli 05428 is a commonly used nuclear lacZ reporter of slit expression. We characterized Slit‐GFP expression pattern because sli 05428 nuclear LacZ expression at early and mid L3 stages was very low and difficult to distinguish from background. Slit full‐length protein can be cleaved into large N‐terminal (Slit‐N) and short C‐terminal (Slit‐C) fragments. Slit‐FL and Slit‐N are more tightly associated with the cell surface, whereas Slit‐C is mostly shed into the extracellular space (Brose et al., 1999). The GFP tag in this Slit‐GFP reporter line was located between amino acids 398‐399, in the second LRR repeat, so in the Slit‐N terminal fragment. Thus, the GFP signal of the Slit‐GFP reporter stays in the membrane of the slit expressing cells. Slit signal for both slit‐lacZ and Slit‐GFP reporters is shown in red. ClC‐a + membranes are labeled with 05423 ClC‐a‐GAL4/UAS‐mCD8‐mRFP and shown in green. Glia nuclei are labeled with anti‐Repo antibody (blue). (A, B) Horizontal views through the VNC showing nuclear LacZ signal (red, A, A’) and membrane Slit‐GFP signal (red, B, B′) in midline glia. (C, D) Frontal views of late L3 optic lobes. (C) Nuclear LacZ signal (red) can be seen in Xgo and medulla neurons as previously reported (Suzuki et al., 2016; Tayler et al., 2004), as well as in cortex glia. In early pupal stages, LacZ expression in Xgo is stronger than in late L3 (data not shown). (D) Membrane Slit‐GFP signal (red) is seen in the same cell types as slit‐LacZ: Xgo, cortex glia, and medulla neurons. Thus, the Slit‐GFP expression pattern is the same as the one observed with slit‐lacZ. midg, midline glia; sa‐cxg, surface‐associated cortex glia; pag, palisade glia; Xgo, outer chiasm glia; mn, medulla neuron. Scale bars represent 10 μm [file GLIA-67-2374-s013.tif]
